# Chelation-Driven Self-Assembly of Luminescent Magnesium Coordination Cages

**DOI:** 10.1021/jacs.6c02366

**Published:** 2026-05-14

**Authors:** Nianfeng Ouyang, Tanya K. Ronson, Huangtianzhi Zhu, Xiang Sun, Jesús Mosquera, Jonathan R. Nitschke

**Affiliations:** † Yusuf Hamied Department of Chemistry, 2152 University of Cambridge, Cambridge, CB2 1EW, U.K.; ‡ Cavendish Laboratory, 2152 University of Cambridge, Cambridge, CB3 0HE, U.K.; § CICA−Centro Interdisciplinar de Química e Bioloxía, Facultade de Ciencias, 16737 Universidade da Coruña, Campus de Elviña, 15071, A Coruña, Spain

## Abstract

The construction of discrete coordination cages using s-block metal ions is challenging due to the weak and electrostatic nature of their coordination bonds, which can lead to the formation of mixtures of products that include intractable coordination polymers, rather than well-defined structures. The alkali and alkaline earth elements are also weaker templates for imines, as they are more oxophilic than transition metals. Here we describe a strategy to overcome these challenges by employing a chelating tris­(pyridyl)­aldehyde subcomponent to define the vertices of magnesium-templated cages. This subcomponent constrains the flexible coordination sphere of magnesium, enabling the assembly of three distinct coordination cage structure types: edge-bridged and face-capped tetrahedra, and a heteroleptic trigonal prism. These hosts displayed diverse binding properties for a range of guests. The two magnesium-based tetrahedral cages also luminesce upon illumination, a feature absent in their transition-metal counterparts. Our work thus provides a general strategy for accessing discrete s-block coordination cages and introduces magnesium coordination cages as a new class of luminescent supramolecular materials.

## Graphical abstract

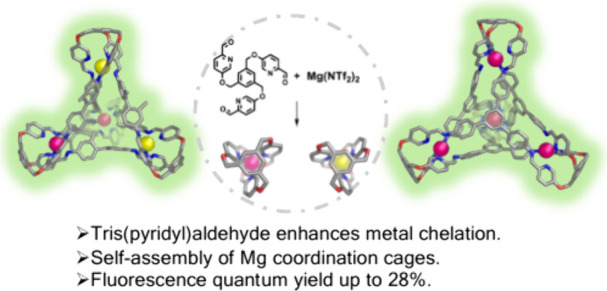

The serendipitous synthesis of a tetra-magnesium complex by Saalfrank in 1988 ^1^ marked the dawn of rationally designed coordination cages as a research field.^2^ These discrete, hollow architectures, formed by the self-assembly of metal ions and organic ligands, have since been developed for a wide range of applications, including separations,^3–6^ catalysis,^7–9^ and sensing.^10–12^ Most cages have been built using transition metal ions,^13–22^ whose directional d-orbital interactions with ligands help to govern the geometry of the final structure. The construction of metal–organic cages from s-block metal ions, while highly desirable due to their abundance and biocompatibility, remains a formidable synthetic challenge.^23–26^ This difficulty arises because coordination bonds to s-block cations are predominantly nondirectional and electrostatic.^27^ The highly flexible coordination spheres of these cations thus can bind to ligands in many different ways, which can lead to the formation of intractable, extended coordination polymers^28^ rather than discrete, soluble cages.

This challenge is relevant to the production of novel luminescent materials. While coordination cages provide a robust platform for creating solution-phase emitters, the cations they incorporate are almost exclusively transition metals or lanthanides.^29–34^ These components often introduce issues of cost, cytotoxicity,^35–37^ and luminescence quenching.^29,38–40^ Magnesium, as an abundant and nontoxic element, is an ideal candidate for creating biocompatible luminescent coordination cages, but its use in such structures has been hampered by the aforementioned challenges.

Here we report a strategy that overcomes the difficulty in using magnesium to template cages by using a tris­(formylpyridine) subcomponent. The enhanced chelation ability and higher density of connections of the resulting imine-based ligand constrains the coordination sphere of Mg^2+^, enabling the self-assembly of three distinct coordination cages: two tetrahedra, **1** and **2**, and a trigonal prism **3**. The host–guest properties of these cages were investigated, revealing that **2** displayed binding affinities comparable to a known Fe_4_L_4_ analogue,^41^ and **3** effectively encapsulated adamantane dimers. Both magnesium-based tetrahedral cages exhibited distinctive fluorescence upon irradiation at 375 nm, a property absent from their zinc congeners. Cage **1** proved to be a particularly strong emitter with a quantum yield of 28%. Its formation thus demonstrated a robust and general pathway toward biocompatible luminescent coordination cages based on s-block metals.

Tris­(formylpyridine) subcomponent **A**, consisting of a central benzene panel and three flexible arms, was synthesized from commercially available 5-hydroxypicolinaldehyde and 1,3,5-tris­(bromomethyl)­benzene in one step (Scheme S1). Compared with other subcomponents reported to form good ligands for s-block metal cations,^23^ the benzene core creates a metal-binding pocket, which strikes a balance between rigidity (from the benzene core) and flexibility (from the −CH_2_–O– side chains). This property enables Mg to form cages.

The reaction of tris­(formylpyridine) subcomponent **A**, 2,2′-dimethylbenzidine **B**, and Mg­(NTf_2_)_2_ in acetonitrile produced cage **1** as the exclusively observed product (Scheme S4). Despite the complex ^1^H NMR spectrum under ambient conditions (Figure S5), the composition of **1** was confirmed by high-resolution electrospray mass spectrometry (HR-ESI-MS) (Figures S6 and S7). The spectral complexity at room temperature may reflect both restricted rotation about the single bond connecting the two phenyl rings in subcomponent **B**, resulting from steric hindrance imposed by the ortho-methyl substituents, and the possible coexistence of multiple diastereomeric cage forms. Variable-temperature NMR (VT-NMR) spectra recorded from 298 to 363 K revealed the coalescence at elevated temperatures of multiple sharp resonances arising from different biphenyl rotamers, at high temperature giving a single set of signals matching *T*-symmetric cage **1** (Figure S8), which were fully assigned by other 2D NMR measurements under 343 K (Figures S9–11). This behavior suggests configurational interconversion enabled by the lability of the magnesium coordination environment at elevated temperature. Diffusion-ordered ^1^H NMR spectroscopy (DOSY) showed that all signals share the same diffusion coefficient of 4.11 × 10^–10^ m^2^·s^–1^ (Figure S12), which corresponds to a solvodynamic radius of 15.9 Å.

Single crystal X-ray diffraction (SC-XRD) revealed an *S*_4_-symmetric framework for **1** (Figure 1b), with two Δ and two Λ Mg^2+^ centers and an average Mg–Mg distance of 12.9 Å. The two phenyl rings defining each edge lie almost perpendicular to one another due to steric clash from the attached methyl groups, with an average torsional angle of 82°. The orientations of these methyls break the *S*_4_ symmetry of the framework, with some pointing inward to occupy the cavity, others pointing toward the centers of the cage faces, and still others pointing outward.

**Figure 1:**
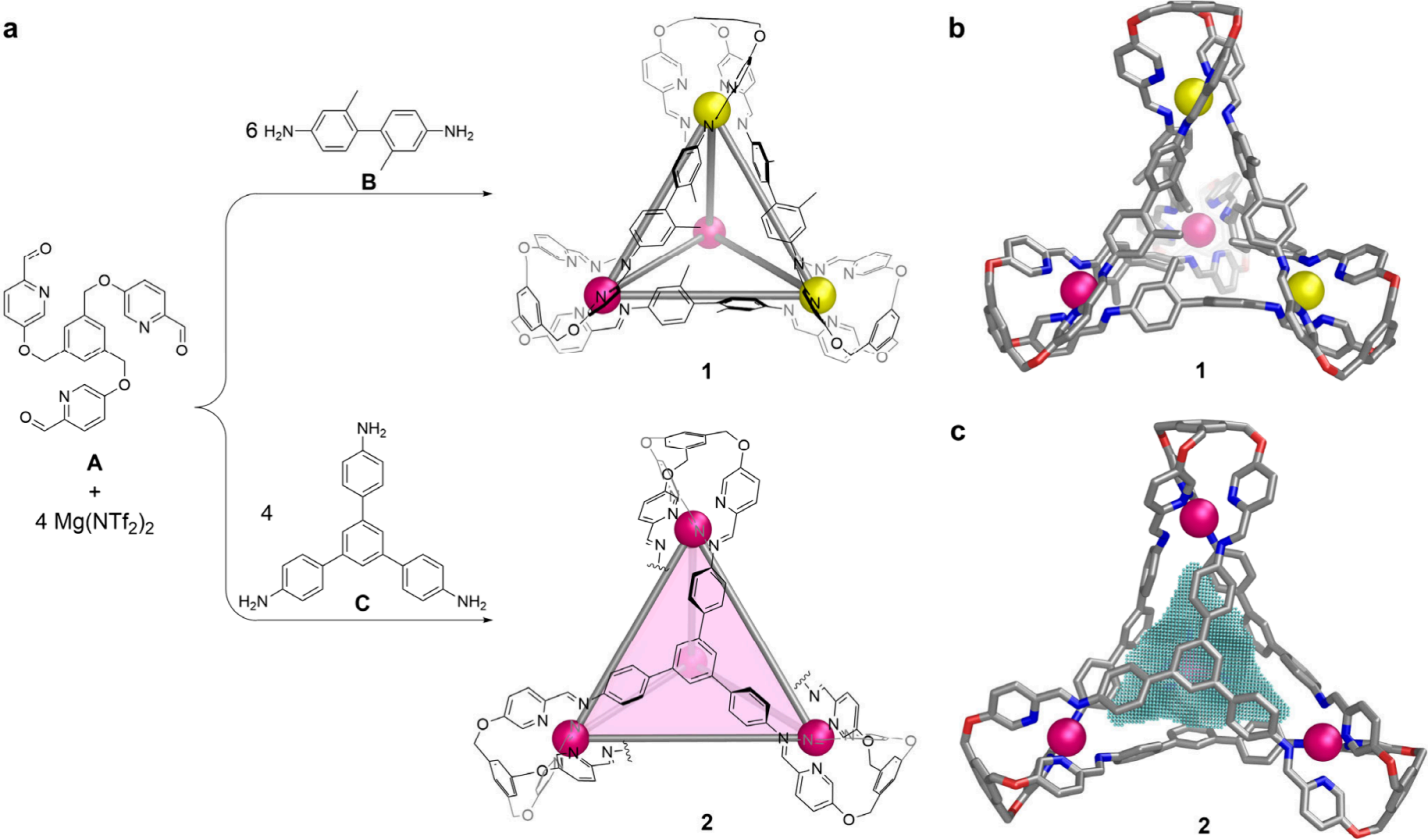
(a) Synthesis of edge-bridged cage **1** and face-capped cage **2**. (b) Single-crystal X-ray structure of **1**. (c) Single-crystal X-ray structure of **2**, with the cavity shown in mesh. Magnesium cations with Λ stereochemistry are shown as magenta spheres, and Δ as yellow.

The presence of the methyl groups limits the cavity volume of cage **1**, thus preventing it from binding any guests but the small anions I^–^, Br^–^, and ClO_4_^–^ (Figure S56), a behavior also observed for an analogous edge-bridged Fe_4_L_6_ cage.^42^ The limited guest binding may be a consequence of the large portals of the edge-bridged cage, which allow small guest molecules to escape more readily than from face-capped analogues. Interactions with guests are also limited by the presence of the cavity-filling methyl groups and by the paucity of protons capable of CH–anion interactions.

The reaction of subcomponents **A** and **C** with Mg­(NTf_2_)_2_ produced cage **2** as the uniquely observed product (Figure 1, Scheme S5). ^1^H NMR signals for **2** (Figure S23) all showed the same DOSY diffusion coefficient, corresponding to a solvodynamic radius of 14.8 Å (Figure S24). Two-dimensional NMR spectra enabled the assignment of all proton signals (Figures S26–S28). The ^19^F NMR spectrum showed one peak corresponding to free triflimide (Figure S29), suggesting that the anion did not bind in the cage cavity. Notably, the reaction of **C** and Mg­(NTf_2_)_2_ with 2-formylpyridine (in place of **A**) under otherwise identical conditions did not produce any discrete product, underscoring the critical role of enhanced chelation in magnesium cage formation (Figure S30).

SC-XRD analysis of crystals obtained via slow diffusion of diethyl ether into an acetonitrile solution of the cage confirmed the composition of cage **2**. All four Mg^2+^ centers within a single cage adopt the same Δ- or Λ-handedness (Figure 1c), indicating *T* point symmetry, consistent with the single set of signals observed in the ^1^H NMR spectrum. No triflimide was found in the internal cavity, in agreement with the single ^19^F NMR signal observed in solution. The cavity volume was calculated to be 228 Å^3^ (SI section 6).

Given the structural similarity between cage **2** and a previously reported Fe_4_L_4_ cage—both incorporating subcomponent **C** and having nearly identical cavity volumes (228 vs 229 Å^3^)—we examined the same set of guests for **2** that were observed to bind in the Fe_4_L_4_ cage.^41^ Cage **2** bound C5–C7 (hetero)­cycles, and chloroform and carbon tetrachloride, but no binding was observed toward C5–C8 linear hydrocarbons or aromatics (Supporting Information section 4.3). While the two cages behaved similarly toward these neutral guests, their interactions with anions differed markedly. Cage **2** bound I^–^ and ClO_4_^–^ within its cavity, as evidenced by upfield shifts and slight peak broadening of cage protons in the ^1^H NMR spectra, whereas the addition of Br^–^ and Cl^–^ prompted cage decomposition, likely due to magnesium halide precipitation. In contrast, the Fe_4_L_4_ cage did not bind anions.^41^ These observations highlight that cavity volume alone does not determine binding ability. Our work thus highlights the necessity of further studies to gauge the influence of factors such as cage rigidity, framework density of connections, and metal center identity on guest binding.

Subcomponents **A** and **C**, along with tetraaniline subcomponent **D**, were observed to self-assemble into a heteroleptic trigonal prismatic structure **3** (Figure 2a). Due to the tendency of the subcomponents to form insoluble polymeric products,^43^ the formation of **3** as the exclusively observed product required the presence of excess subcomponents **A**, **D**, and Mg­(NTf_2_)_2_, guest templation, and extended heating (see SI section 3.3 for detailed information). DOSY analysis showed a single diffusion coefficient for all signals, indicating a solvodynamic radius of 21.3 Å (Figure S47). Two-dimensional NMR spectra allowed the assignment of all proton signals (Figures S48–S51). The composition of trigonal prism **3** was confirmed by HR-ESI-MS (Figures S52 and S53). In contrast, the reaction of only subcomponents **A** and **D** with Mg­(NTf_2_)_2_ in acetonitrile led to the formation of insoluble polymeric material rather than a discrete cage species, as evidenced by ^1^H NMR spectroscopy (Figure S54).

**Figure 2:**
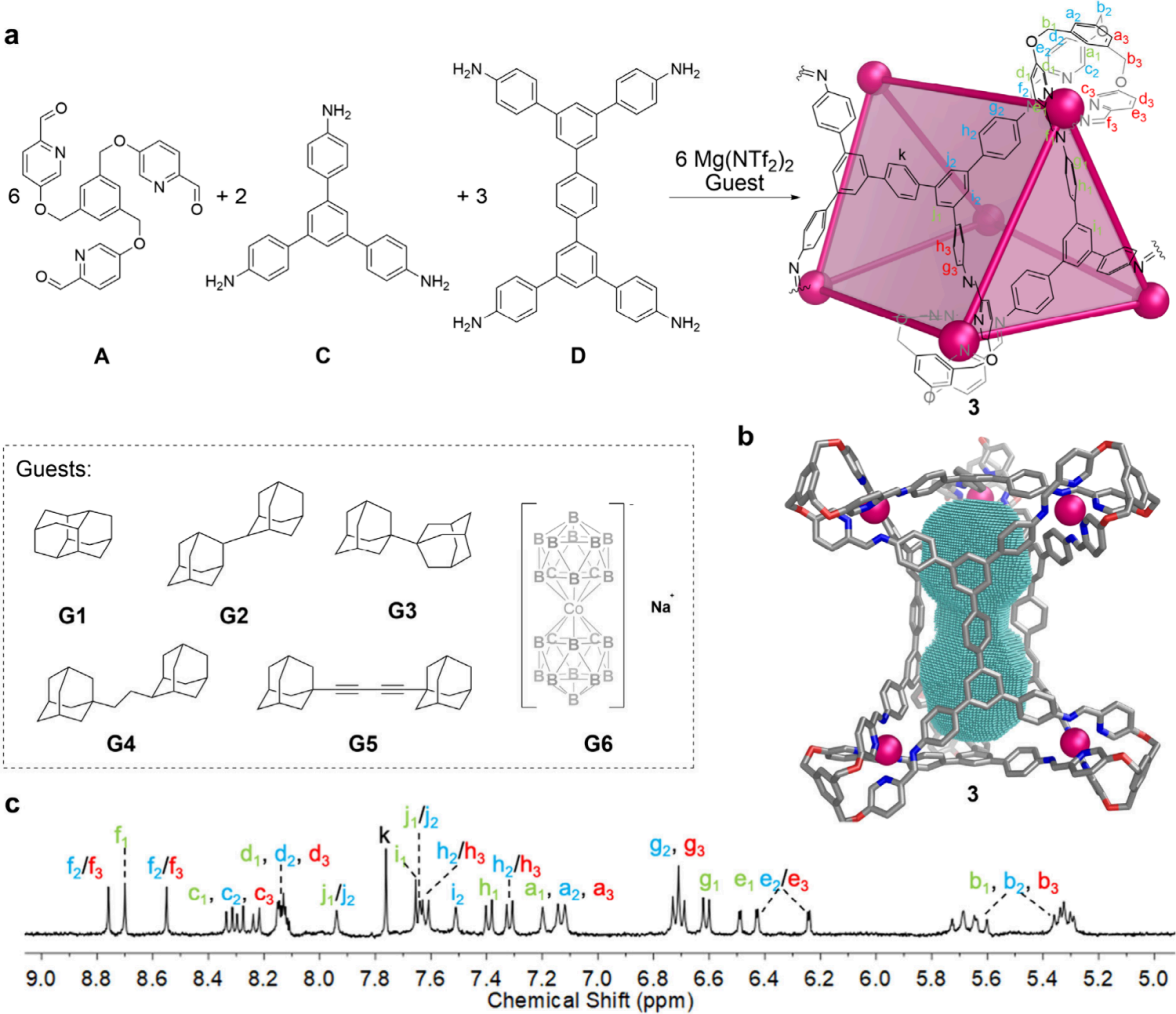
Preparation, characterization, and guest molecules for trigonal prism **3**. (a) Self-assembly of trigonal prism **3** and the library of guests. (b) Calculated structure of **3** at the MM3 level of theory, with the cavity shown in mesh. (c) ^1^H NMR spectrum of **3** encapsulated guest **G5**.

The cavity size of cage **3** was calculated to be 683 Å^3^ based on the modeled structure (SI section 6). The dumbbell-shaped cavity inspired us to explore the binding of linear guest molecules that are narrower in the center but bulkier at the ends, to effect 1:1 binding, or small bulky molecules that occupy roughly half of the cavity (1:2 binding mode).^44^ Accordingly, the guests shown in Figure 2a were screened, and observed to bind in fast exchange (Figure 2a and SI section 4.4).

The magnesium cages were found to luminesce under UV illumination, prompting us to study the photoluminescent properties of **1** and **2**. To provide a point of comparison, we prepared versions of **1** and **2** in which Zn^2+^ was used in place of Mg^2+^. Zinc was chosen due to its similarity to magnesium: both are commonly observed in only the +2 oxidation state, and both divalent cations are of similar size.^45^ The reaction between subcomponents **A**, **B**, and Zn­(NTf_2_)_2_ yielded tetrahedral cage **1** **′**, with analytical data (SI section 3.1.2) consistent with a product isostructural to **1**. In contrast, the reaction between subcomponents **A**, **C**, and Zn­(NTf_2_)_2_ produced a mixture (denoted as **2** **′** for simplification) of a tetrahedron and a helicate in a ratio of 1.2:1 following equilibration (SI section 3.2.2).

Compared with cage **1** **′**, the emission from cage **1** was notably more intense (Figure 3a). The photoluminescence quantum yield (PLQY) of cage **1** was measured to be 28%, six times greater than the 4.7% PLQY measured for **1** **′**. Cage **2** (PLQY: 1.2%) was likewise 12 times more emissive than its zinc counterpart **2** **′** (PLQY: 0.10%). Control experiments of acetonitrile solutions with only subcomponents **A**, **B**, and **C**, or mixtures of these subcomponents in the absence of metals, showed negligible luminescence (Figure S65).

**Figure 3:**
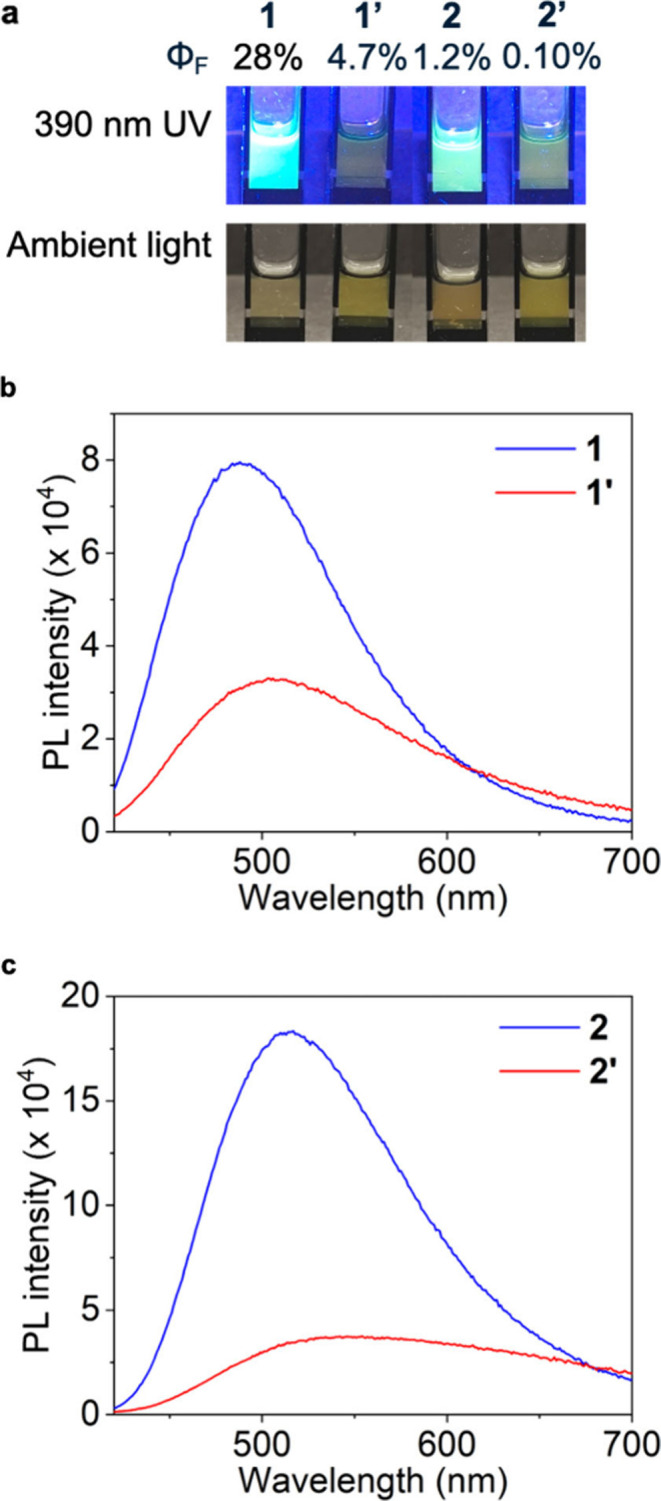
Photoluminescence characterization. (a) Photos of cages under UV and ambient lighting, with the corresponding PLQY values. (b) Steady-state photoluminescence (PL) spectra of cages **1** and **1** **′**. (c) PL spectra of the cages **2** and **2** **′**. The concentration of each sample was 1 mM; excitation was at 405 nm. *Φ*_F_ represents PLQY of the cage.

UV–vis spectra (Figures S67 and S68) indicated that the absorptions of the zinc cages are red-shifted relative to their magnesium congeners. This red-shift indicates enhanced charge-transfer character in the zinc systems, which promotes nonradiative decay pathways and consequently leads to reduced emission intensity. Due to the instrument-limited time resolution, the extracted lifetime values carry uncertainties; however, the decay clearly falls in the subnanosecond regime, characteristic of fluorescence rather than phosphorescence (Figures S74–76).

Compared with many previously reported fluorescent cages,^46–50^ which are mostly constructed from transition-metal or lanthanide ions, our magnesium cages offer several advantages, including lower cost and reduced toxicity. In addition, fluorescence is induced upon cage formation: the subcomponents are essentially nonemissive under identical experimental conditions, whereas the magnesium cage exhibits a quantum yield of up to 28%. Many earlier examples rely on strongly fluorescent chromophores in building blocks, whereas our system demonstrates that even weakly emissive subcomponents can produce bright emission when organized in a rigid cage framework.

Tris­(formylpyridine) subcomponent **A** thus enabled the incorporation of magnesium cations into metal–organic cages featuring N-donor ligands. This strategy proved effective with di-, tri-, and tetra-topic amine subcomponents, and the crystal structures of both tetrahedral cages **1** and **2** indicated close structural similarities to congeners prepared using the same polyamines, formylpyridines, and first-row d-block metals. Comparative studies between magnesium and iron-based cages suggest that variations in the metal cation and vertex identity had a subtle influence on guest binding behavior. Photoluminescence experiments highlighted the utility of integrating magnesium into these cages, which had much higher luminescence than their zinc congeners. Future work will probe the biocompatibility of these new cages, to gauge their utility in drug delivery.^51^ Their luminescence properties will also be examined as a function of guest presence and identity, to see if these materials might be built into useful sensors.

## Supplementary Material



## Notes

Experimental procedures; NMR characterizations; mass spectrometry data; UV–vis data; photoluminescence data (PDF)
