# Supplementary material for: Chelation-Driven Self-Assembly of Luminescent Magnesium Coordination Cages
Source: J Am Chem Soc. 2026 May 14;148(20):20328–33. doi: 10.1021/jacs.6c02366 (PMC13220303; doi:10.1021/jacs.6c02366)
Supplement: Supplementary file 1 [file ja6c02366_si_001.pdf]

Supporting Information for

Chelation-Driven Self-assembly of Luminescent Magnesium Coordination Cages

Nianfeng Ouyang,[†] Tanya K. Ronson,[†] Huangtianzhi Zhu,[‡] Xiang Sun,[†] Jesus Mosquera,[^] and Jonathan R. Nitschke[*,†]*

[†]Yusuf Hamied Department of Chemistry, University of Cambridge, CB2 1EW, U.K.

[‡]Cavendish Laboratory, University of Cambridge, Cambridge, CB3 0HE, U.K.

^CICA−Centro Interdisciplinar de Química e Bioloxía, Facultade de Ciencias, Universidade da Coruña, Campus de Elviña, 15071, A Coruña, Spain

*email: jrn34@cam.ac.uk

Table of Contents

S1 General Information 3

S2 Synthesis of subcomponents 4

S2.1 Synthesis of subcomponent A 4

S3 Synthesis and characterization of metal-organic cages 9

S3.1 Synthesis and characterization of 1 and 1’ 9

S3.2 Synthesis and characterization of 2 and 2’ 23

S3.3 Synthesis and characterization of 3 41

S4 Host-guest studies 51

S4.1 General procedure 51

S4.2 Host-guest study of cage 1 51

S4.3 Host-guest study of cage 2 52

S4.4 Host-guest studies of cage 3 54

S5 X-ray crystallography 59

S6 Volume calculations 62

S7 Study of photoluminescent properties 63

S7.1 General Procedure 63

S7.2 Other luminescent photos and spectra 63

S8 Stability Tests in Various Solvents 74

S8.1 General Procedure 74

S8.2 Stability in Water 74

S8.3 Stability in Methanol 75

S8.4 Stability in Dimethyl Sulfoxide 77

S8.5 Stability in Chloroform 78

S9 References 81

S1 General Information

Unless otherwise stated, all chemicals were purchased from commercial suppliers and used without further purification. Self-assembly reactions were conducted in distilled or deuterated acetonitrile.

NMR experiments were conducted with the following NMR spectrometers: Bruker 400 MHz Avance III HD Smart Probe (for ^{1}H NMR, ^{19}F NMR, and DOSY measurements), Bruker 500 MHz Avance III HD Smart Probe (for variable temperature ^{1}H NMR), Bruker 500 TCI-ATM Cryo and Bruker 700 TCI-ATM Cryo (other 2D NMR measurements). Chemical shifts (δ) were reported in parts per million (ppm) for ^{1}H, ^{13}C and ^{19}F NMR spectra. Chemical shifts were referenced using the residual CD3CN solvent signal (^{1}H = 1.94 ppm, ^{13}C = 118.26 ppm). A C_6F_6 internal standard was added for ^{19}F NMR spectra, with the signal referenced at -164.38 ppm. Coupling constants (*J*) were reported in Hz to 1 decimal place. Signal multiplicity in ^{1}H and ^{13}C NMR spectra was described using the following abbreviations: singlet (s), doublet (d), doublet of doublets (dd), multiplet (m), and broad (br). For DOSY experiments: maximum gradient strength was 6.57 G/cm A. The standard Bruker pulse program, ledbpgp2s, employing a stimulated echo and longitudinal eddy-current delay (LED) using bipolar gradient pulses for diffusion using 2 spoil gradients was utilised. Rectangular gradients were used with a total duration of 1.5 ms. Gradient recovery delays were 1250-1500 μs.

High-resolution electrospray mass spectroscopy (HR-ESI-MS) experiments were conducted with Waters Synapt G2-Si spectrometer (capillary voltage 3.0 kV, cone voltage 10 eV; desolvation temp. 373 K; ionisation temp. 333 K) infused from a Harvard syringe pump at a rate of 4–10 μL min^{-1}.

S2 Synthesis of subcomponents

S2.1 Synthesis of subcomponent **A**

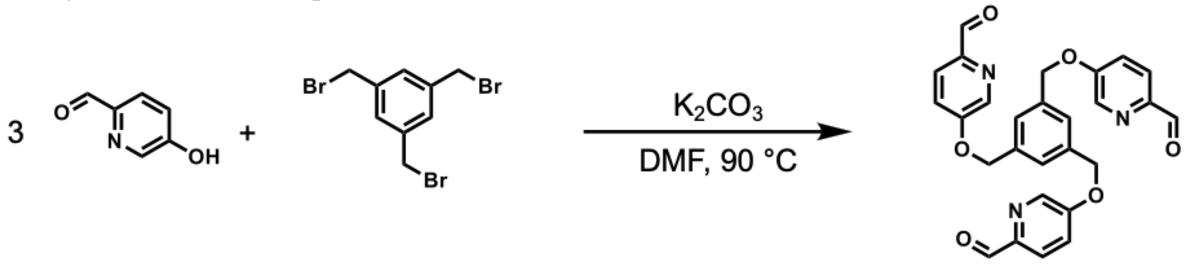

Scheme S1. Synthetic route for subcomponent **A**.

A mixture of 5-hydroxypicolinaldehyde (862 mg, 7.00 mmol, 5.0 equiv.) and potassium carbonate (968 mg, 7.00 mmol, 5.0 equiv.) was dissolved in DMF (20 mL), and then preheated at 90 °C for two hours. Afterwards, 1,3,5-tris(bromomethyl)benzene (500 mg, 1.40 mmol, 1.0 equiv.) was added to the reaction mixture. The mixture was further heated while stirring overnight. The resulting mixture was then allowed to cool to room temperature, then partitioned between dichloromethane and water. The crude product was subject to column chromatography and eluted with 2% methanol in dichloromethane. The product **A** was obtained as a white powder (431 mg, 0.89 mmol, 63.6%). The residual DMF was removed by suction under reduced pressure at 100 °C in an oil bath for two days.

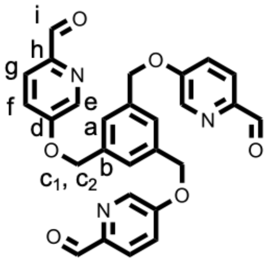

^{1}H NMR (400 MHz, $CDCl_3$, 298 K) δ 10.00 (s, 3H, H_i), 8.51 (d, J = 4 Hz, 3H, H_e), 7.97 (d, J = 8 Hz, 3H, H_f), 7.53 (s, 3H, H_a), 7.37 (dd, J = 8, 4 Hz, 3H, H_g), 5.26 (s, 6H, H_c).

^{13}C NMR (101 MHz, $CDCl_3$, 298 K) δ 191.8 (C_i), 151.7 (C_d), 146.7 (C_h), 138.8 (C_e) , 136.9 (C_b), 126.5 (C_a), 123.3 (C_f), 121.1 (C_g), 69.9 (C_c).

HR-ESI-MS Found m/z = 484.1403 $[M+H]^+$, $C_{27}H_{21}N_3O_6$ requires m/z = 483.4800.

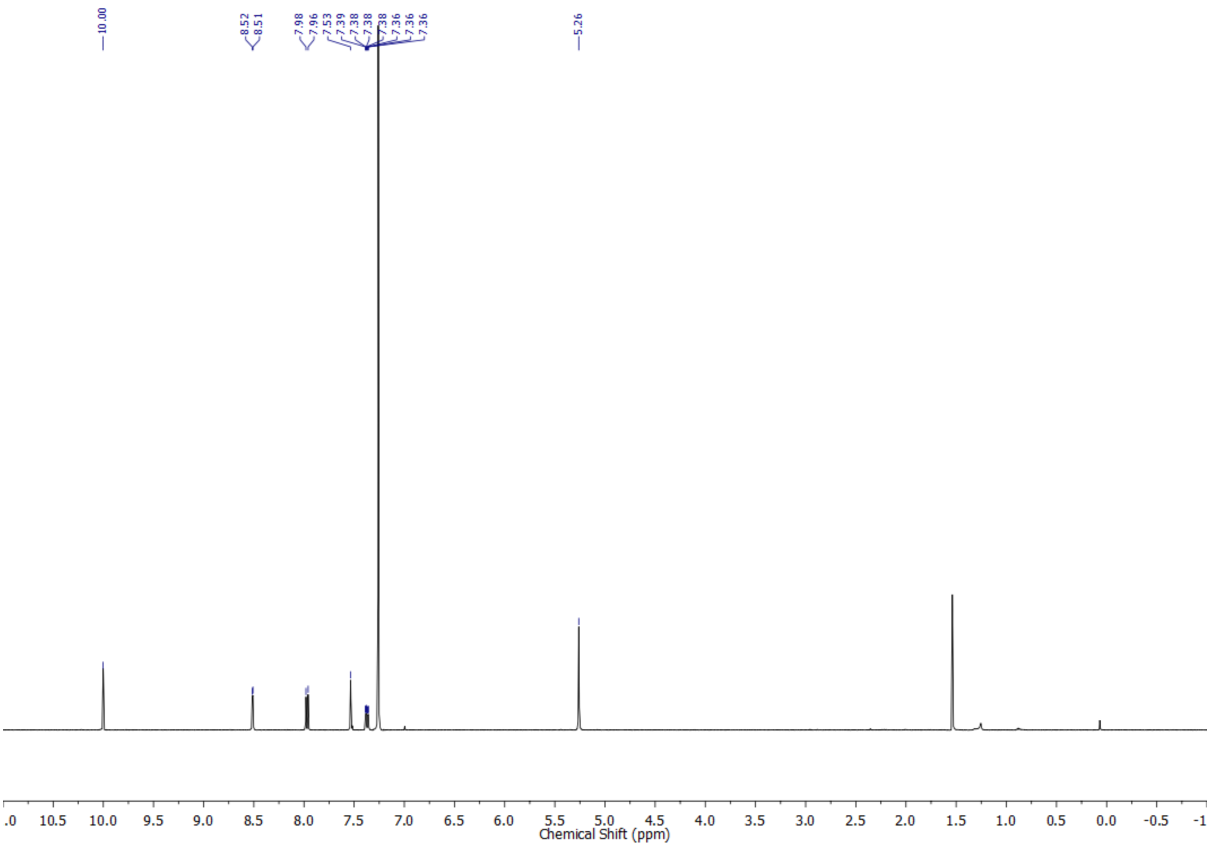

Figure S1. ^{1}H NMR spectrum (400 MHz, $CDCl_3$, 298 K) of **A**.

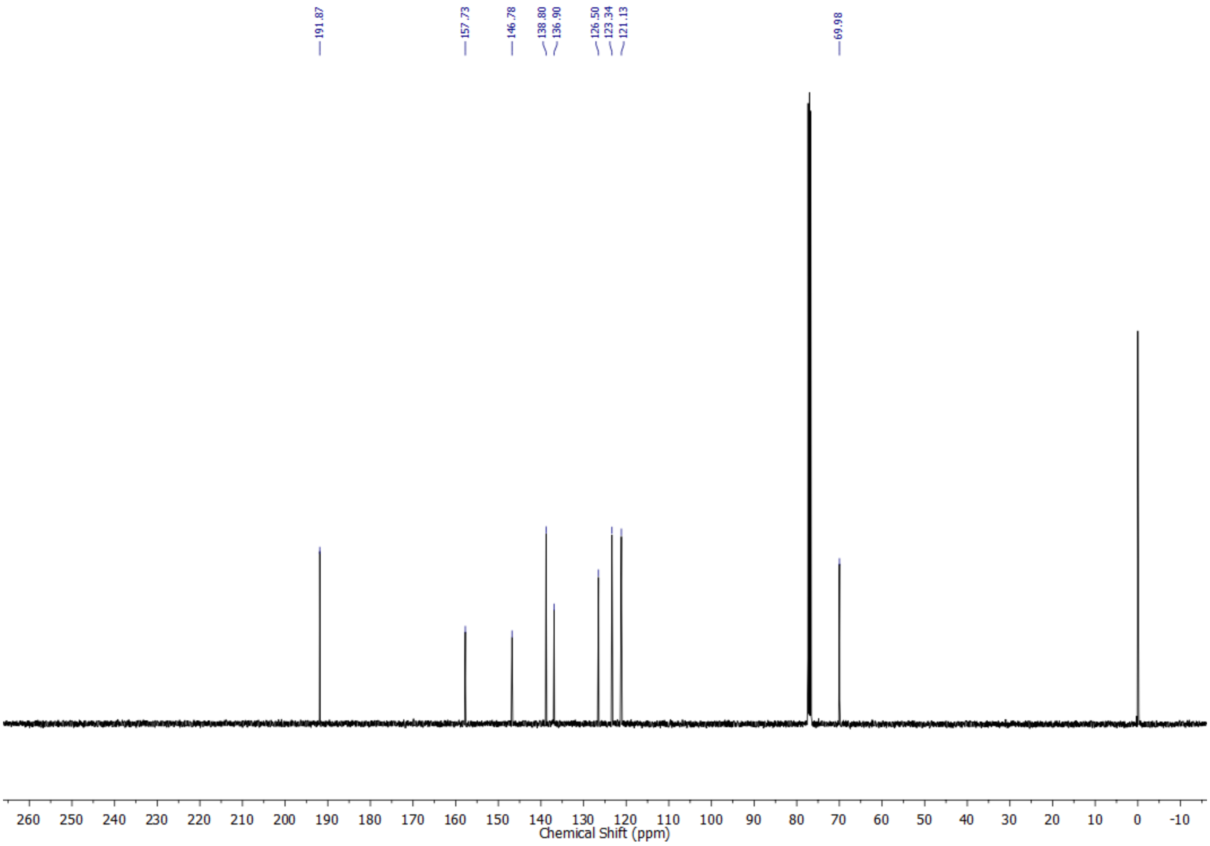

Figure S2. ^{13}C NMR spectrum (101 MHz, $CDCl_3$, 298 K) of **A**.

S2.2 Synthesis of subcomponent D

Reported procedures were followed for the synthesis of subcomponent **D**.[1]

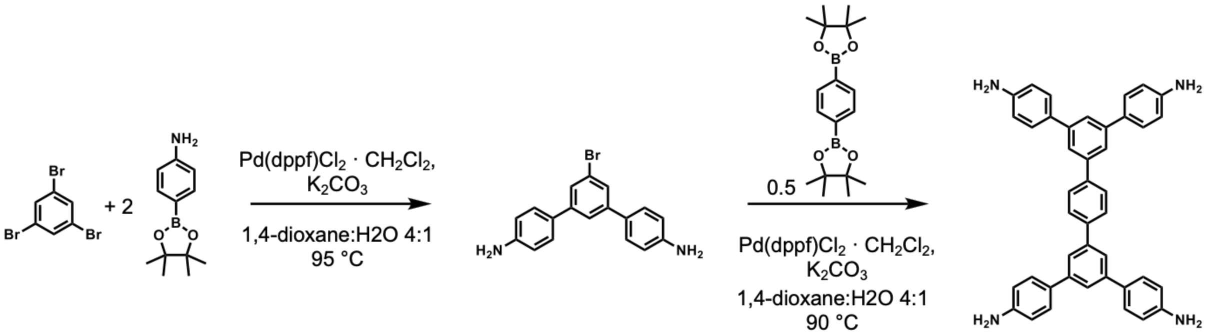

Scheme S2. Synthetic route for subcomponent **D**. All characterization data matched those previously reported.

^{1}H NMR (400 MHz, DMSO-d$_6$, 298 K) δ 7.89 (s, *J* = 1.6 Hz, 4H), 7.68–7.66 (m, 6H), 7.53 (d, *J* = 8 Hz, 4H), 6.70 (d, *J* = 8 Hz, 4H), 5.24 (s, 8H).

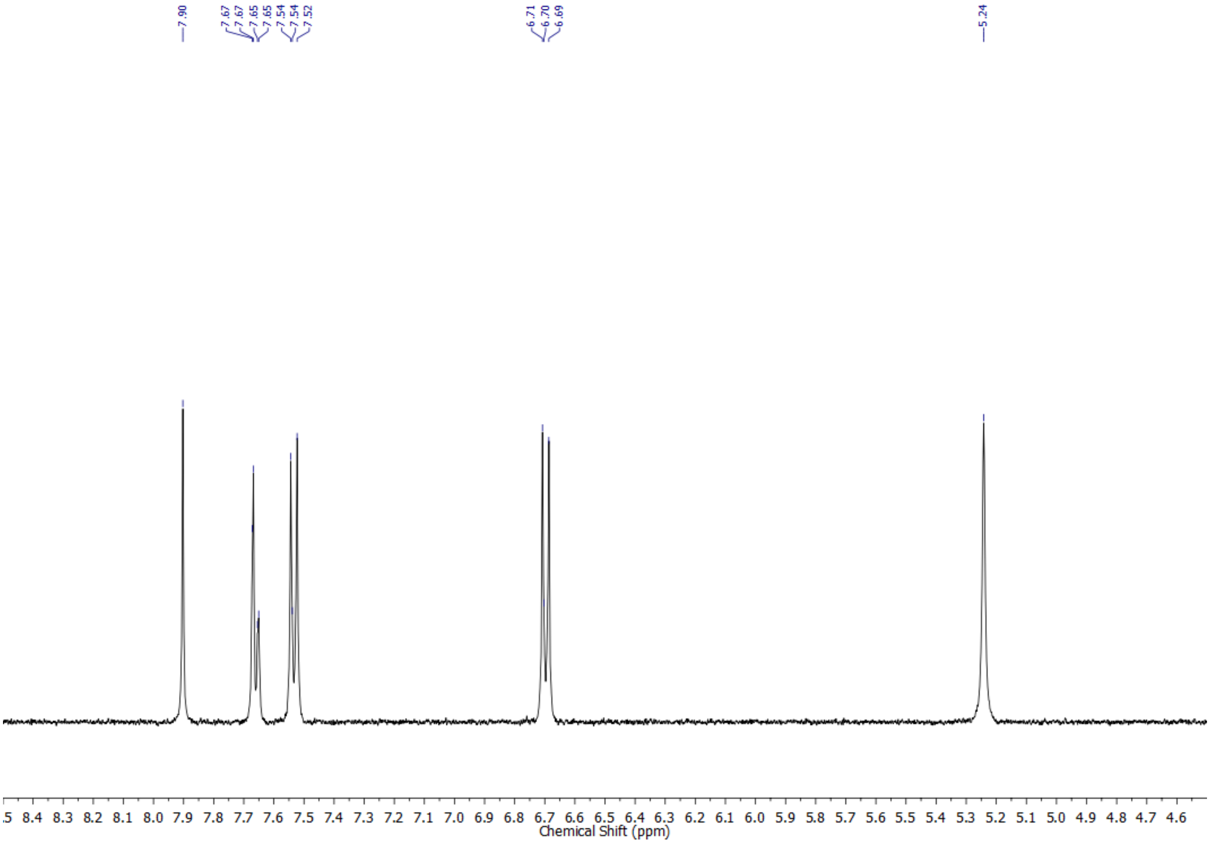

Figure S3. Partial ^{1}H NMR spectrum (400 MHz, DMSO-d$_6$, 298 K) of **D**.

S2.3 Synthesis of guest G5

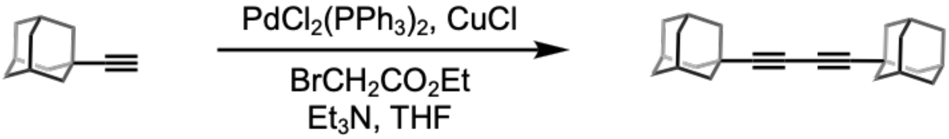

Scheme S3. Synthetic route for guest molecule **G5**.

G5 was synthesized via a modification of a reported procedure.[2] To a solution of 1-ethynyladamantane (140 mg, 0.87 mmol) in THF (24 mL) was added the reactants in the following order: $PdCl_2(PPh_3)_2$ (30 mg, 0.042 mmol), CuI (8 mg, 0.042 mmol), ethyl bromoacetate (123 µL, 1.11 mmol) and Et_3N (246 µL, 1.76 mmol). The reaction mixture was stirrer at room temperature overnight under a N_2 atmosphere. The reaction was quenched with H_2O (20 mL) and then extracted with Et_2O (3 × 20 mL). The organic layers were combined, washed with brine (20 mL), and then dried over Mg_2SO_4. The solvent was removed *in vacuo* and the solid was re-dissolved in dichloromethane. The solution was treated with activated charcoal, filtered through a celite pad, and the filtrate was concentrated to dryness. The product was obtained by recrystallisation from 1,4-dioxane as a crystalline solid (20 mg, 14%). All characterization data matched those previously reported.

^{1}H NMR (400 MHz, $CDCl_3$, 298 K) δ 1.92–1.97 (m, 6H), 1.84–1.88 (m, 12H), 1.64–1.69 (m, 12H).

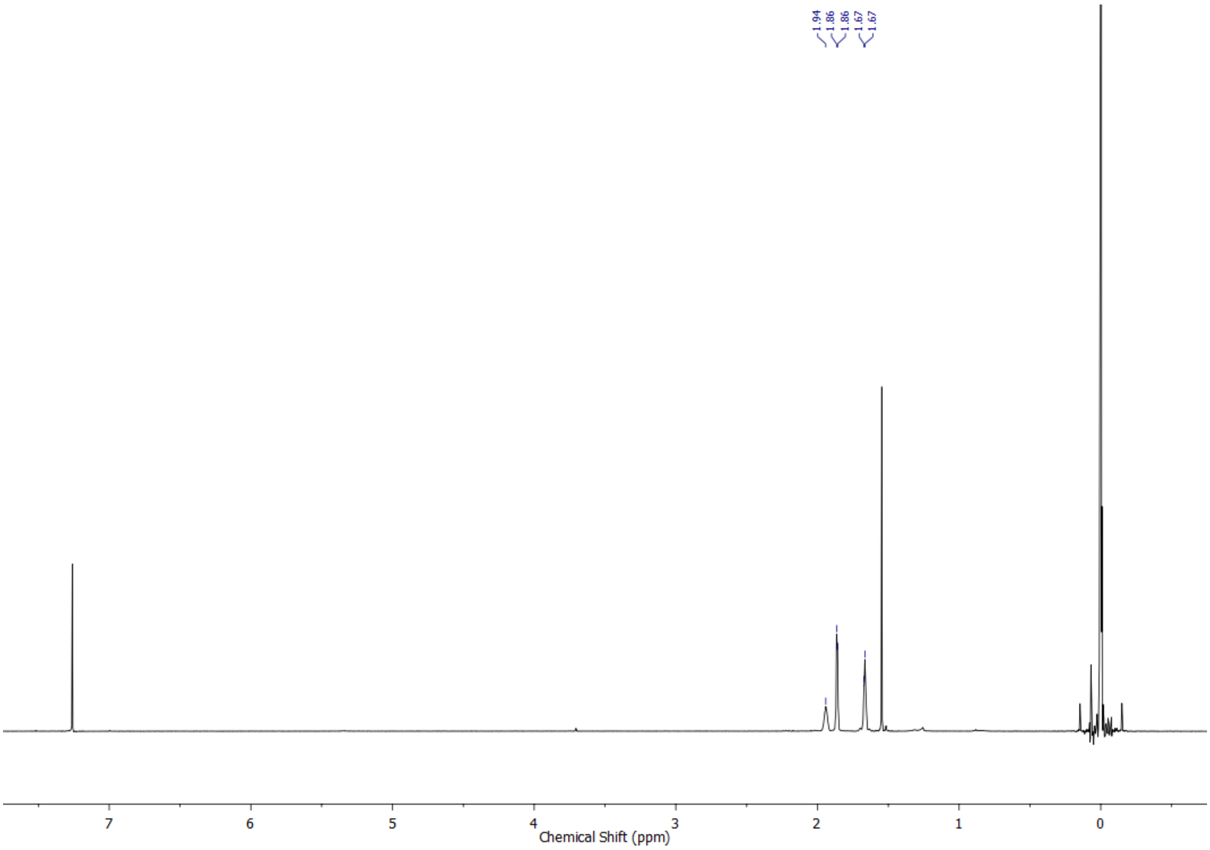

Figure S4. ^{1}H NMR spectrum (400 MHz, $CDCl_3$, 298 K) of guest molecule **G5**.

S3 Synthesis and characterization of metal-organic cages

S3.1 Synthesis and characterization of **1** and **1’**

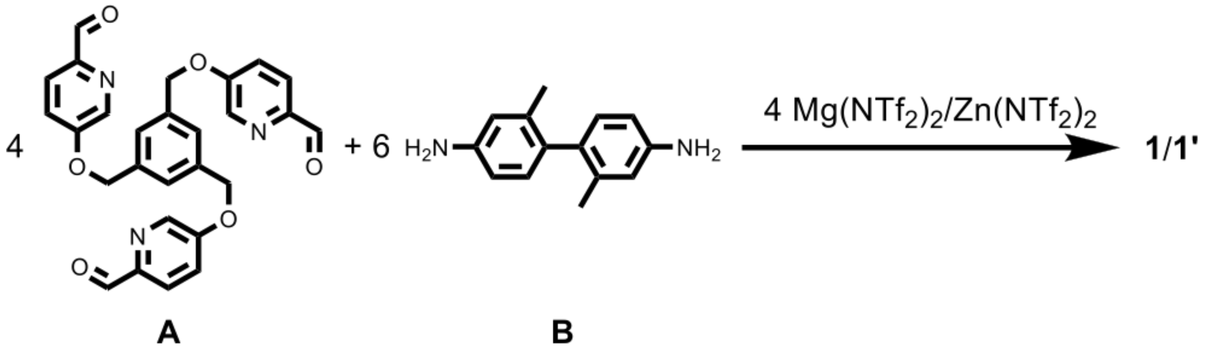

Scheme S4. Subcomponent self-assembly of **1** and **1’**.

The mixture of subcomponent **A** (5.80 mg, 12 µmol, 1 eq.), di-aniline **B** (3.82 mg, 18 µmol, 1.5 eq.), and $Mg(NTf_2)_2$ (7.01 mg, 12 µmol, 1 eq.) or $Zn(NTf_2)_2$ (7.51 mg, 12 µmol, 1 eq.) in deuterated acetonitrile (3 mL) was heated at 70 °C for 24 hours. The reaction mixture was allowed to cool to room temperature and the insoluble by-products were removed by filtration through a glass fiber plug. The filtrate solution was used without further purification.

To obtain solid products, the filtrate was separated in 1 mL aliquots and each sample was diluted with diethyl ether to 15 mL. The resulting precipitate was dried under N_2 flow. The products were obtained as follows:

Cage **1**: light yellow powder (11.42 mg, 71 %)

Cage **1’**: yellow powder (10.38 mg, 63 %)

S3.1.1 Synthesis and characterization of 1

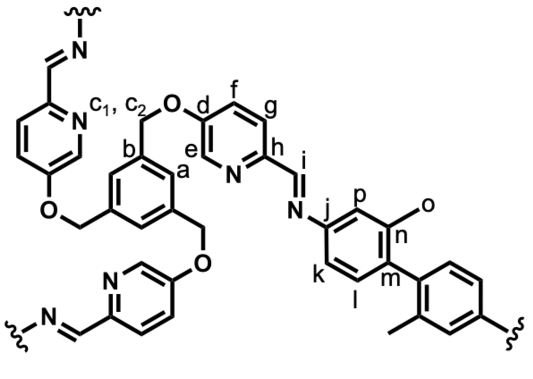

^{1}H NMR (500 MHz, CD_3CN, 343 K) δ 8.54 (s, 12H, H_i), 8.20 (br s, 12H, H_g), 8.11 (br s, 12H, H_e), 7.11 (s, 12H, H_a), 6.95 (br s, 12H, H_l), 6.57–6.50 (m, 12H, H_k), 6.43–6.38 (m, 12H, H_p), 6.31–6.19 (m, 12H, H_f), 5.56 (br s, 12H, H_{c1}), 5.29 (d, J = 10 Hz, 12H, H_{c2}), 1.86–1.78 (m, 36H, H_o).

ESI-HR-MS ([**1**(NTf_2)$_8$] = $C_{192}H_{156}N_{24}O_{12}Mg_4(C_2F_6NO_4S_2)_8$) *m/z* = 481.1575 [**1**+NTf_2]$^{7+}$ (calc. 481.1561), 608.0055 [**1**+2NTf_2]$^{6+}$ (calc. 608.0016), 785.5896 [**1**+3NTf_2]$^{5+}$ (calc. 785.5855), 1052.2165 [**1**+4NTf_2]$^{4+}$ (calc. 1052.2114), 1496.2579 [**1**+5NTf_2]$^{3+}$ (calc. 1496.2545).

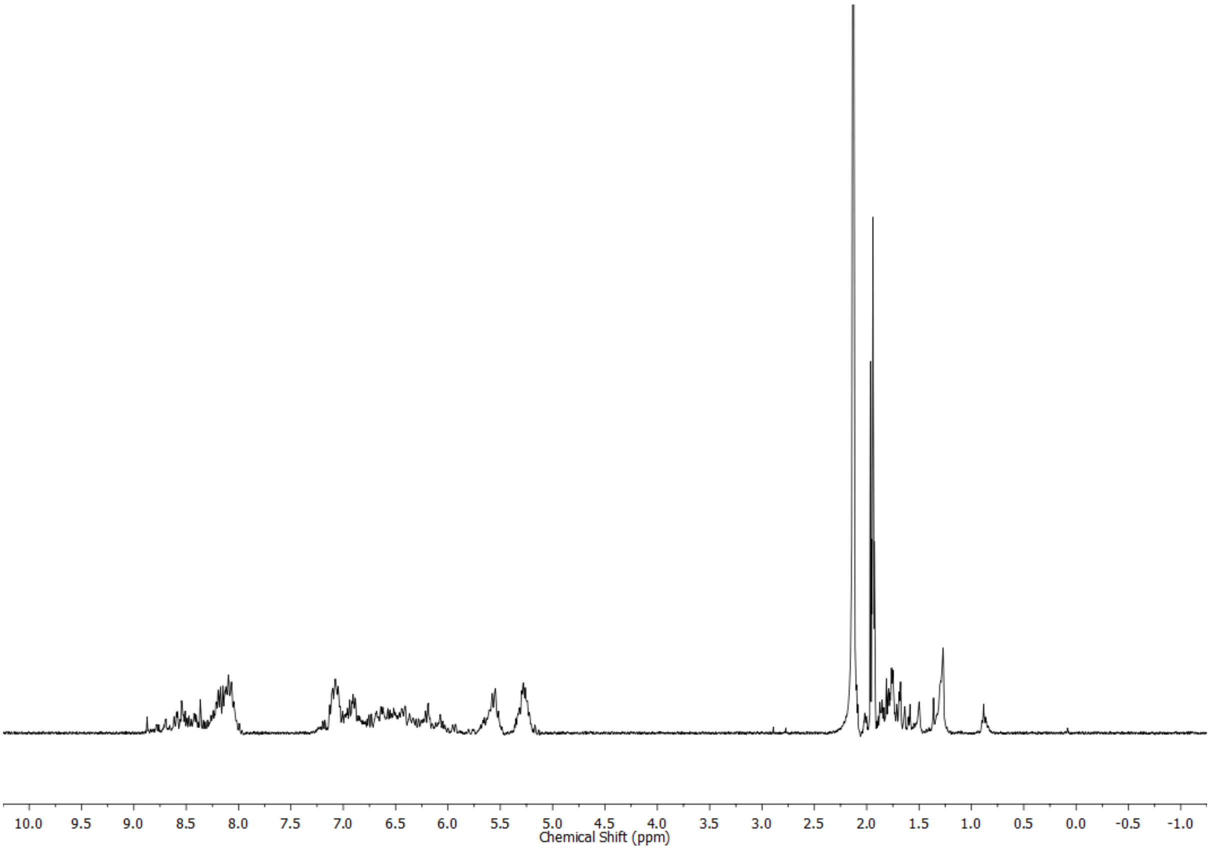

Figure S5. ^{1}H NMR spectrum (400 MHz, CD_3CN, 298 K) of **1**.

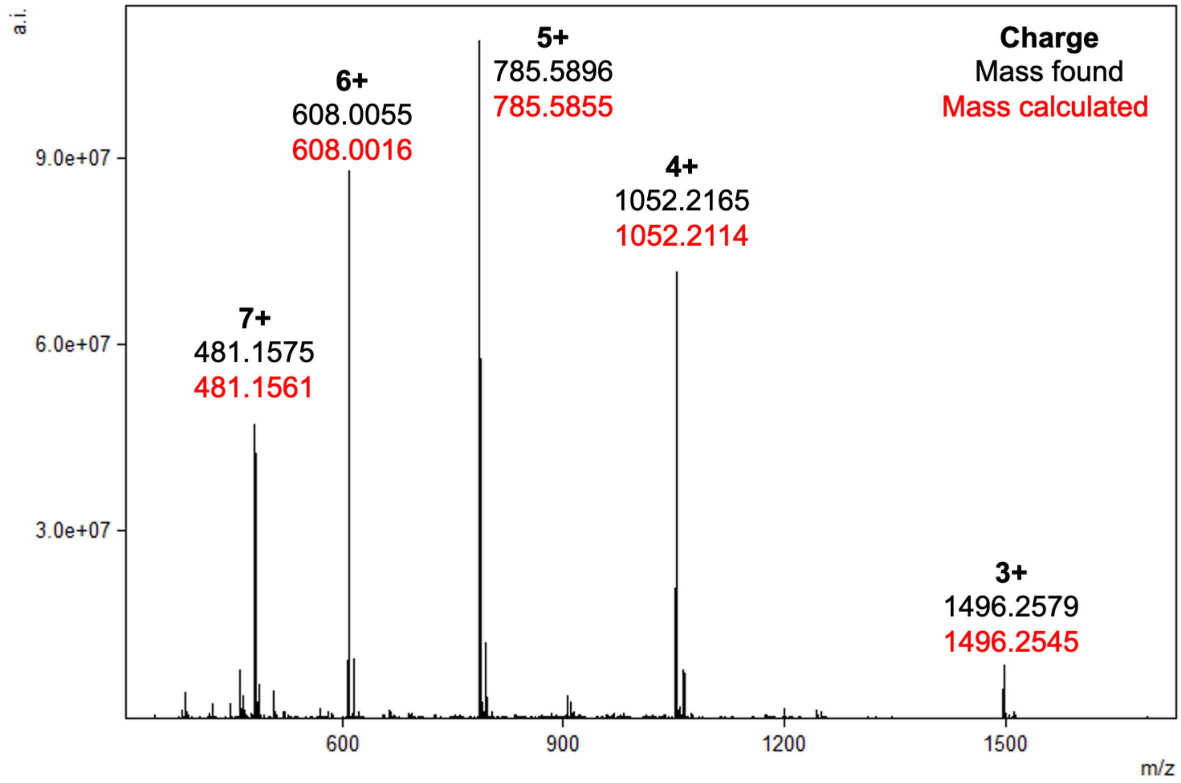

Figure S6. High resolution ESI-mass spectrum of **1**.

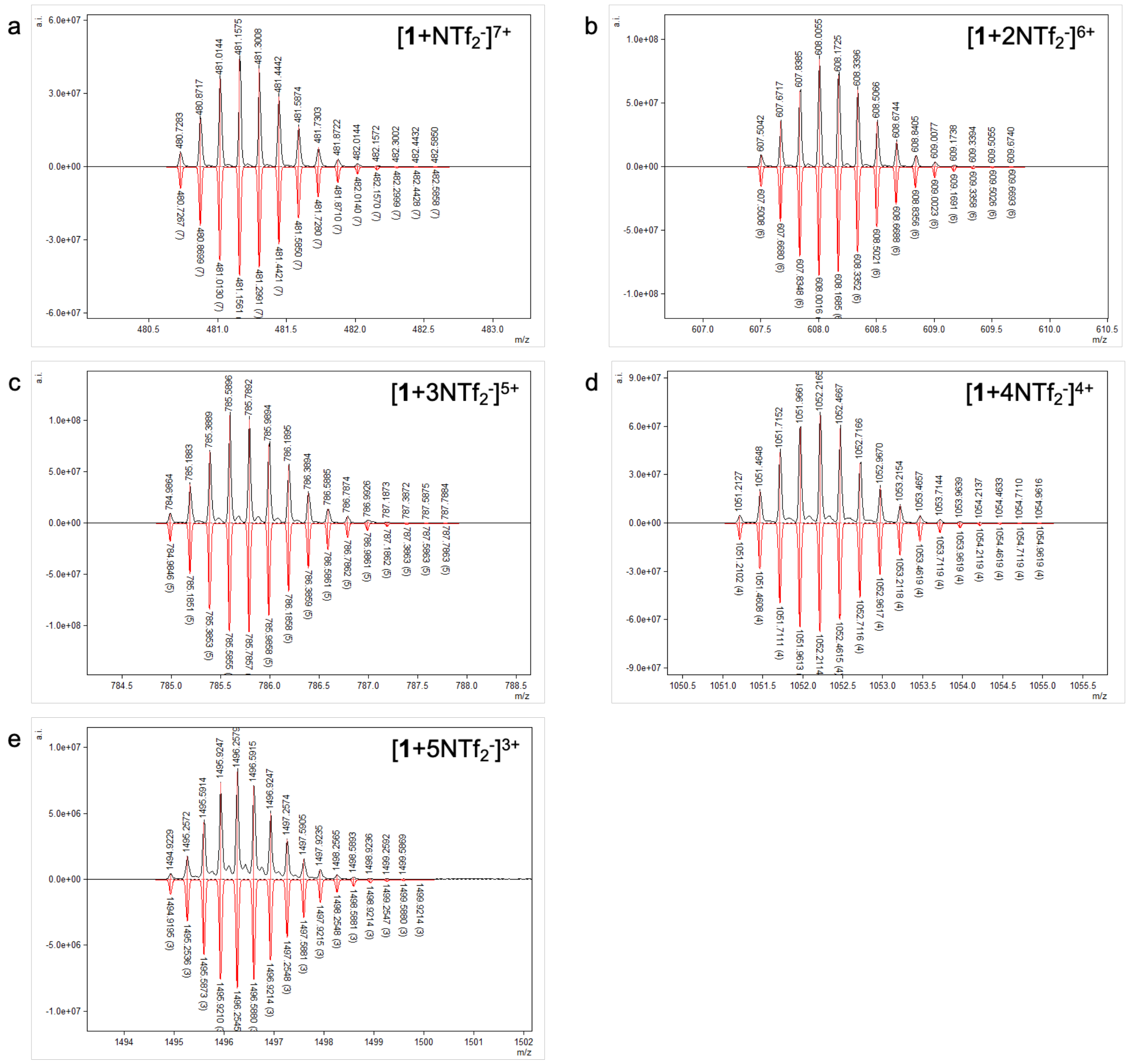

Figure S7. Signals from the HR-ESI-MS spectrum of **1**. Observed and calculated signals for (a) [**1**+NTf_2^-]$^{7+}$; (b) [**1**+$2NTf_2^-$]$^{6+}$; (c) [**1**+$3NTf_2^-$]$^{5+}$; (d) [**1**+$4NTf_2^-$]$^{4+}$; (e) [**1**+$5NTf_2^-$]$^{3+}$.

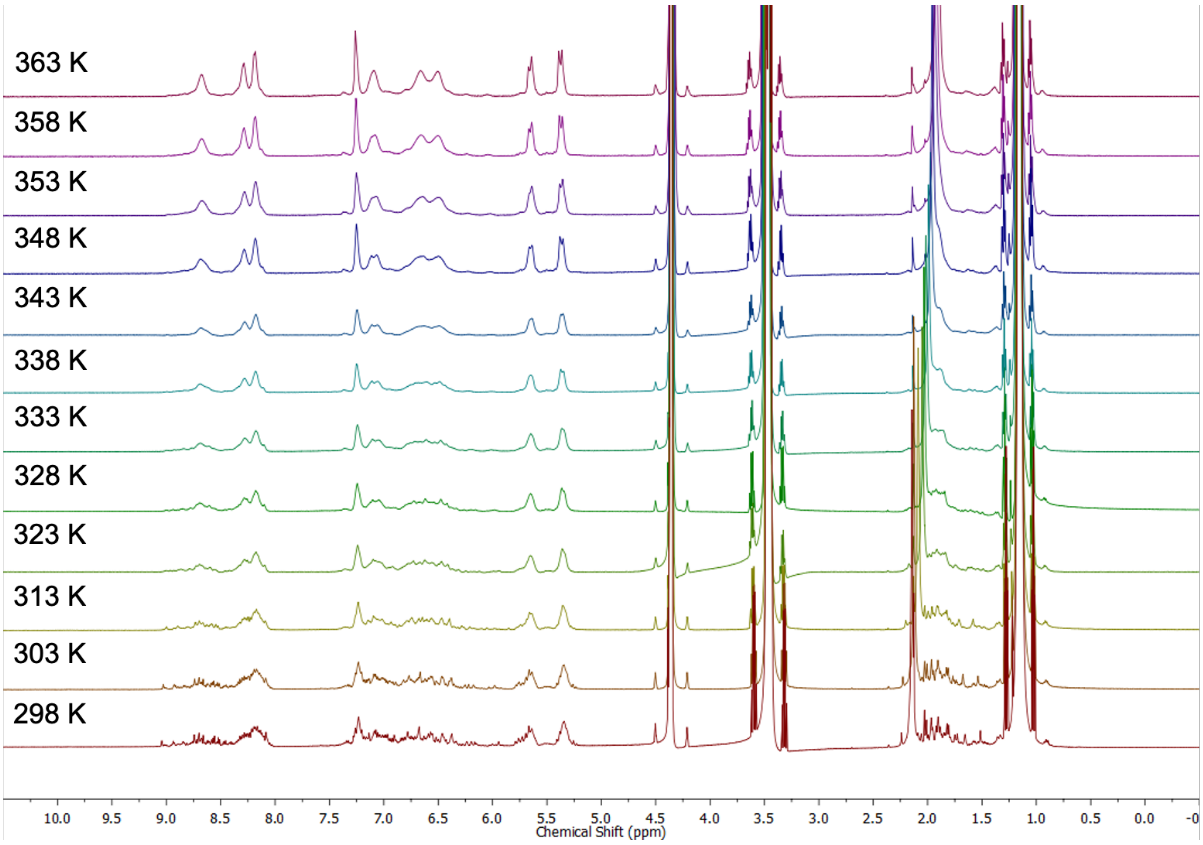

Figure S8. VT spectra (500 MHz, CD_3NO_2) of **1**. CD_3NO_2 was used for a wider range of temperature.

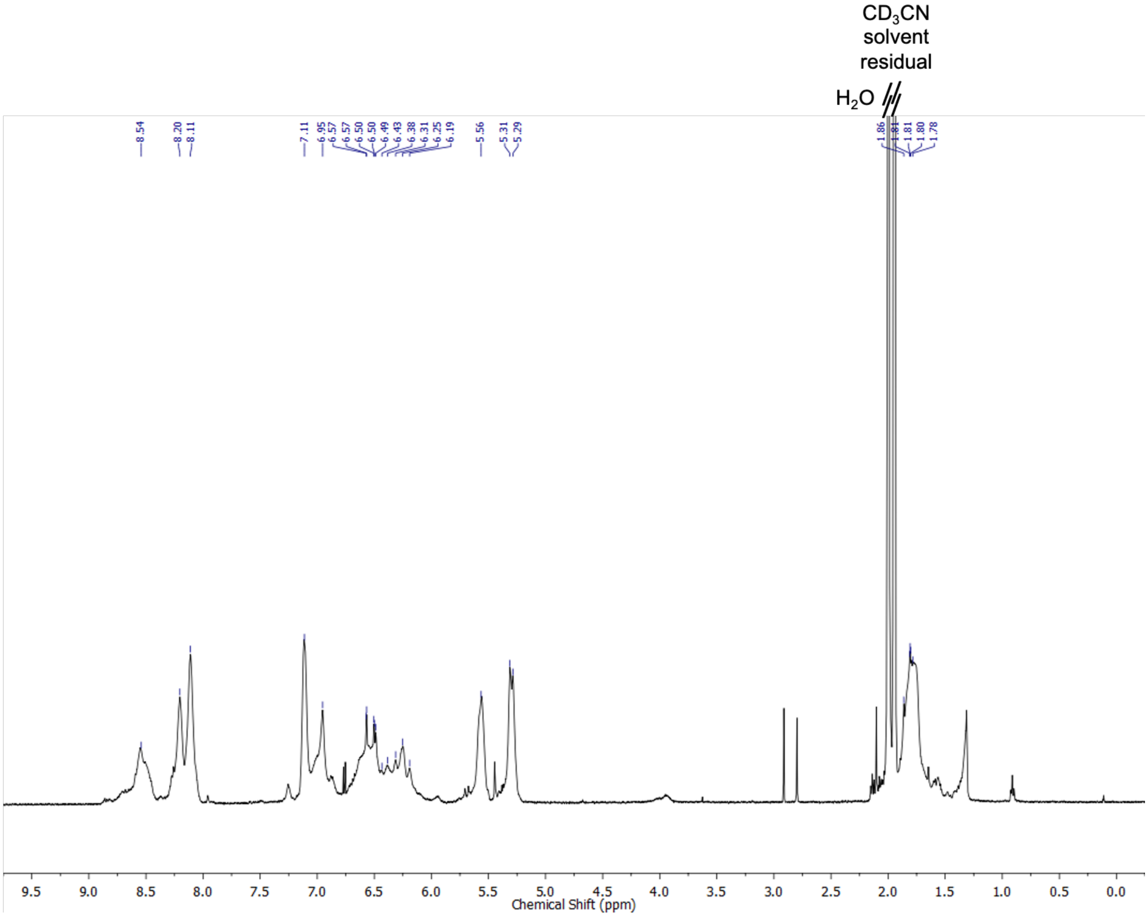

Figure S9. ^1H NMR spectrum (500 MHz, CD_3CN, 343 K) of **1**.

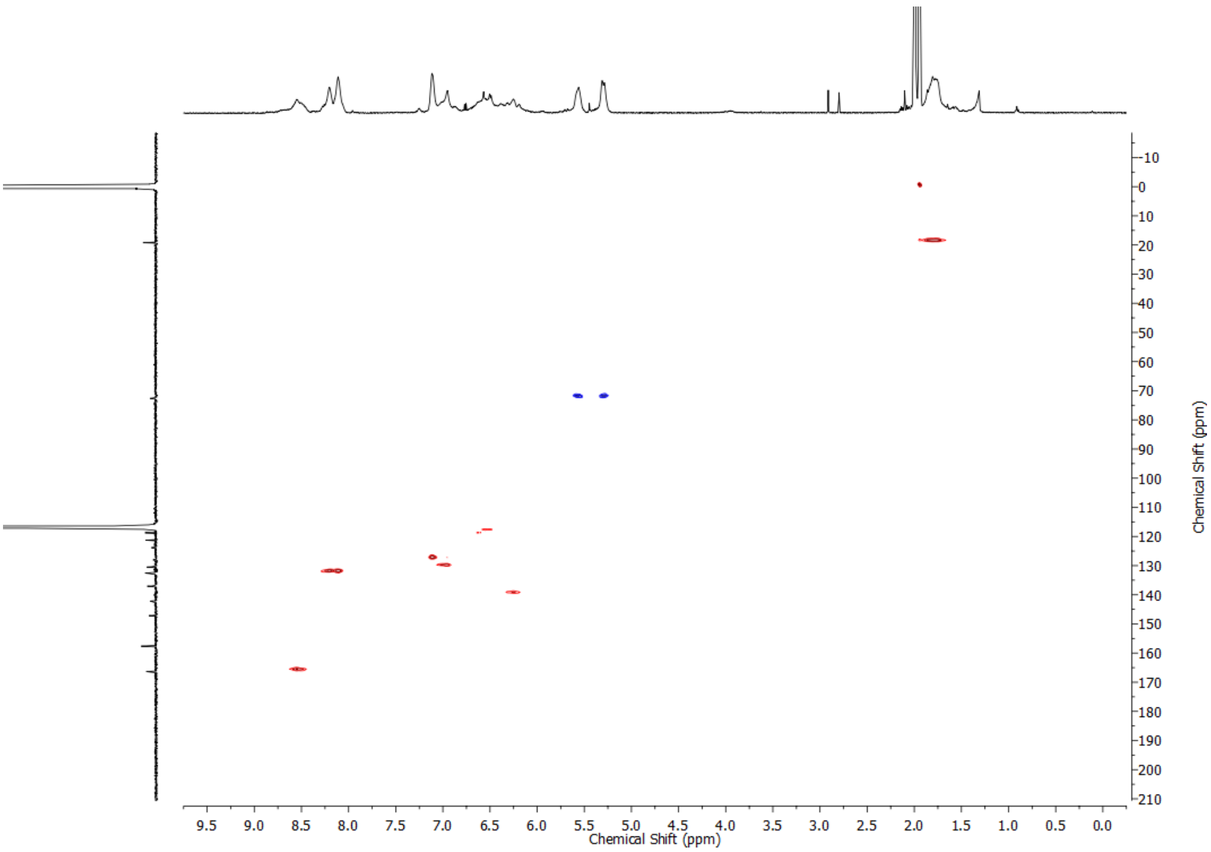

Figure S10. ^{1}H-^{1}H HSQC spectrum (500 MHz, CD_3CN, 343 K) of **1**.

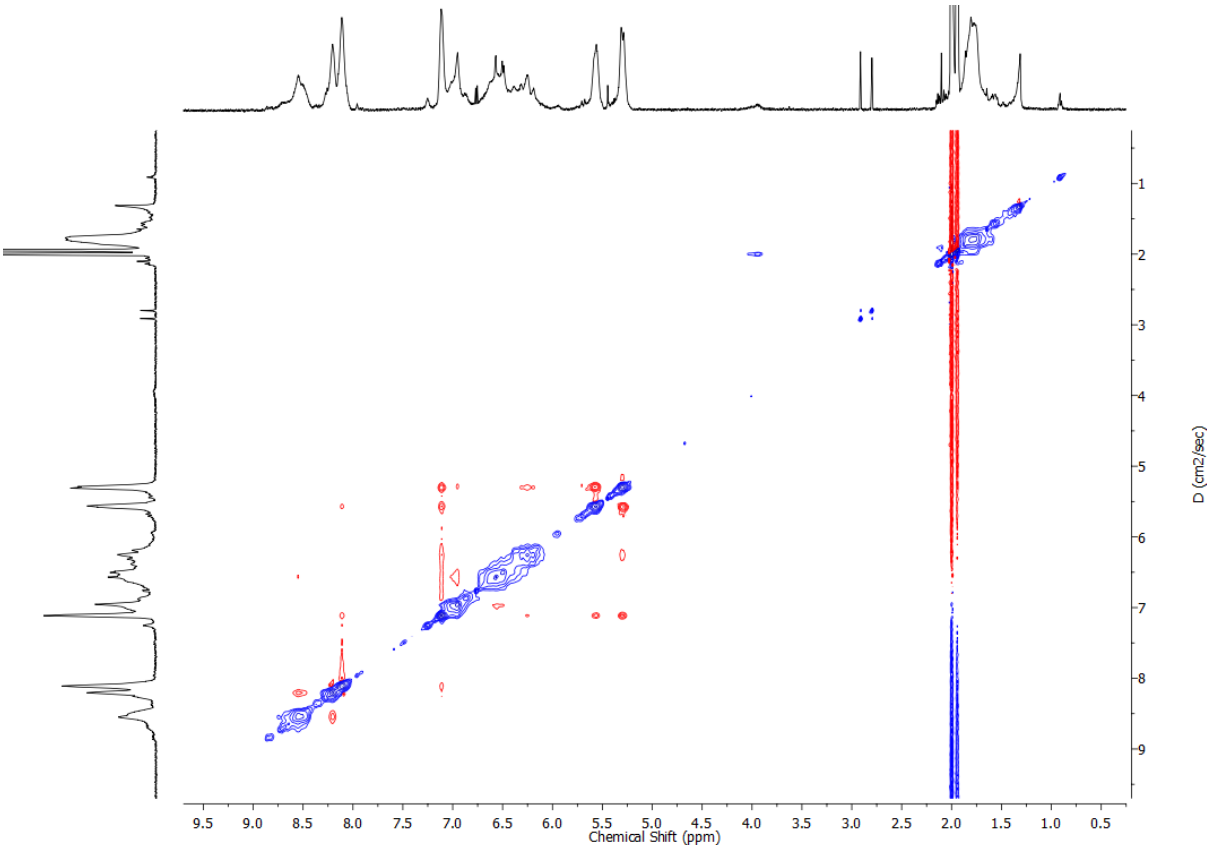

Figure S11. ^{1}H-^{1}H ROESY spectrum (500 MHz, CD_3CN, 343 K) of **1**.

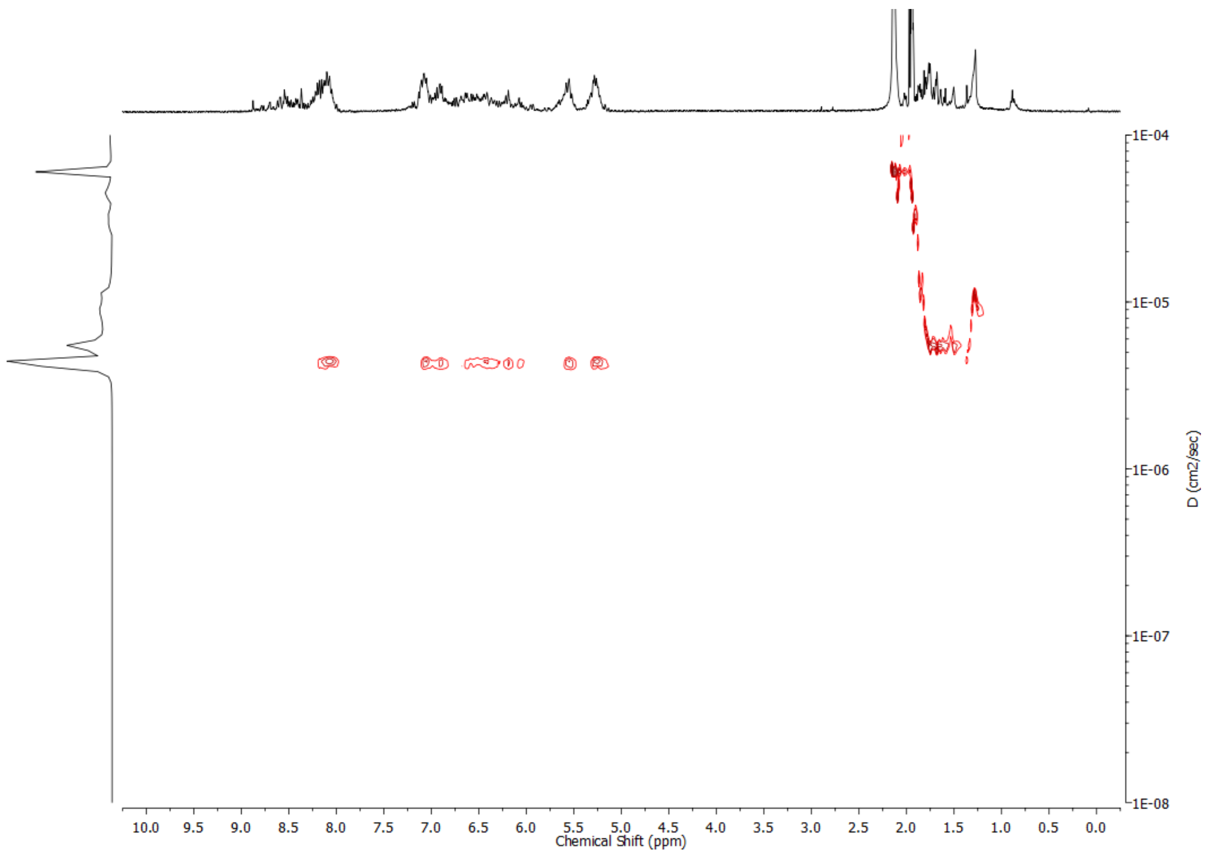

Figure S12. ^{1}H DOSY spectrum (400 MHz, CD_3CN, 298 K) of **1**.

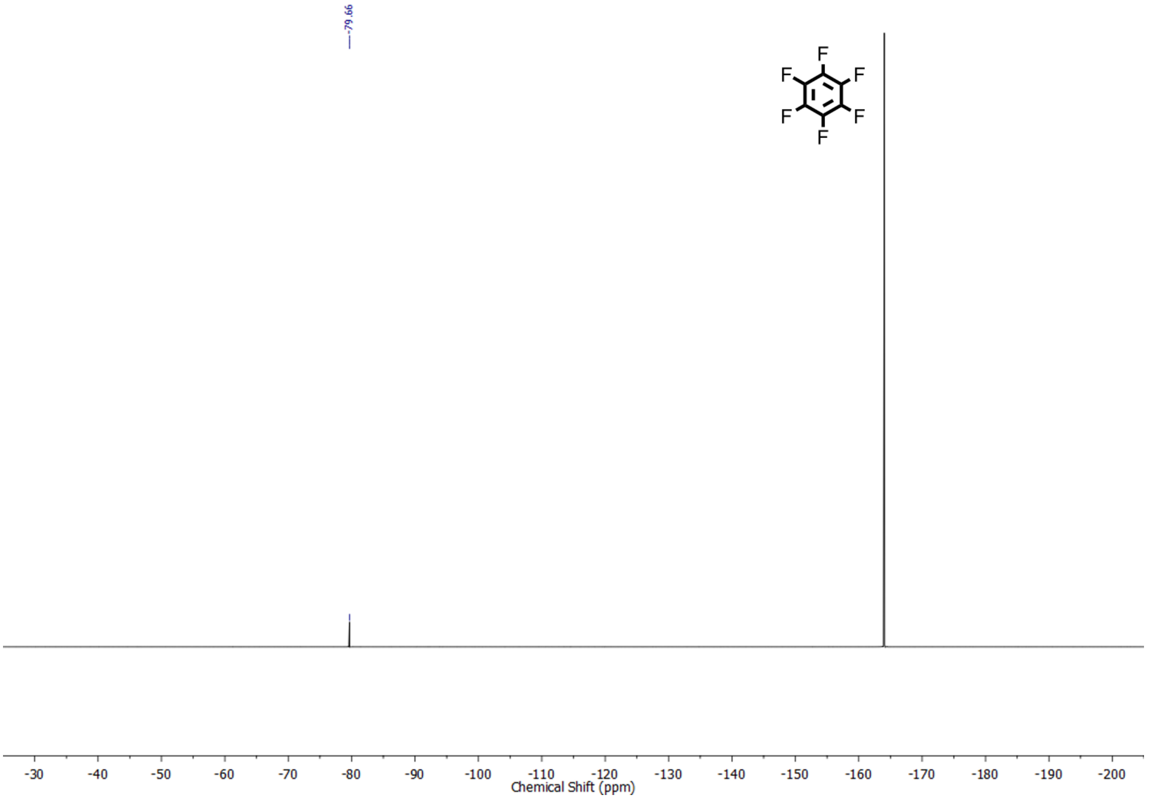

Figure S13. ^{19}F NMR spectrum (376 MHz, CD_3CN, 298 K) of **1**. Referenced to hexafluorobenzene.[3]

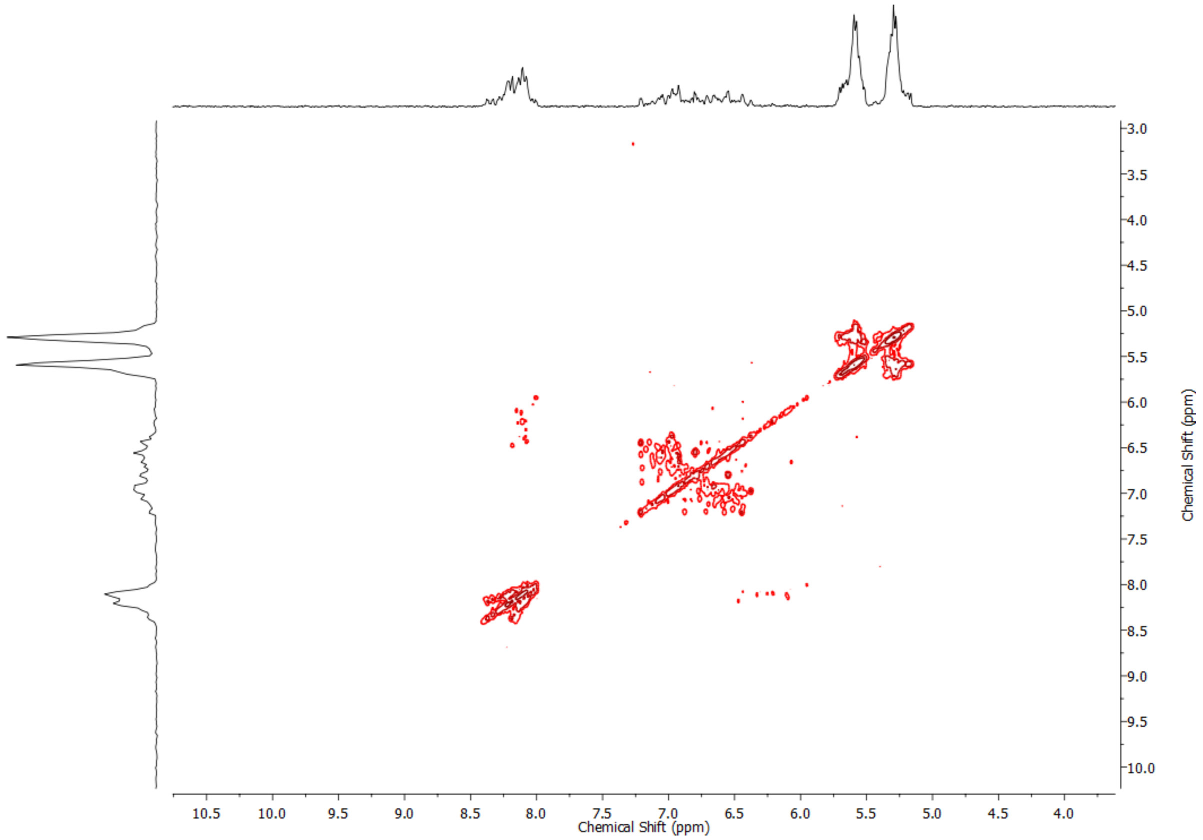

Figure S14. ^{1}H-^{1}H COSY spectrum (500 MHz, CD_3CN, 298 K) of **1**.

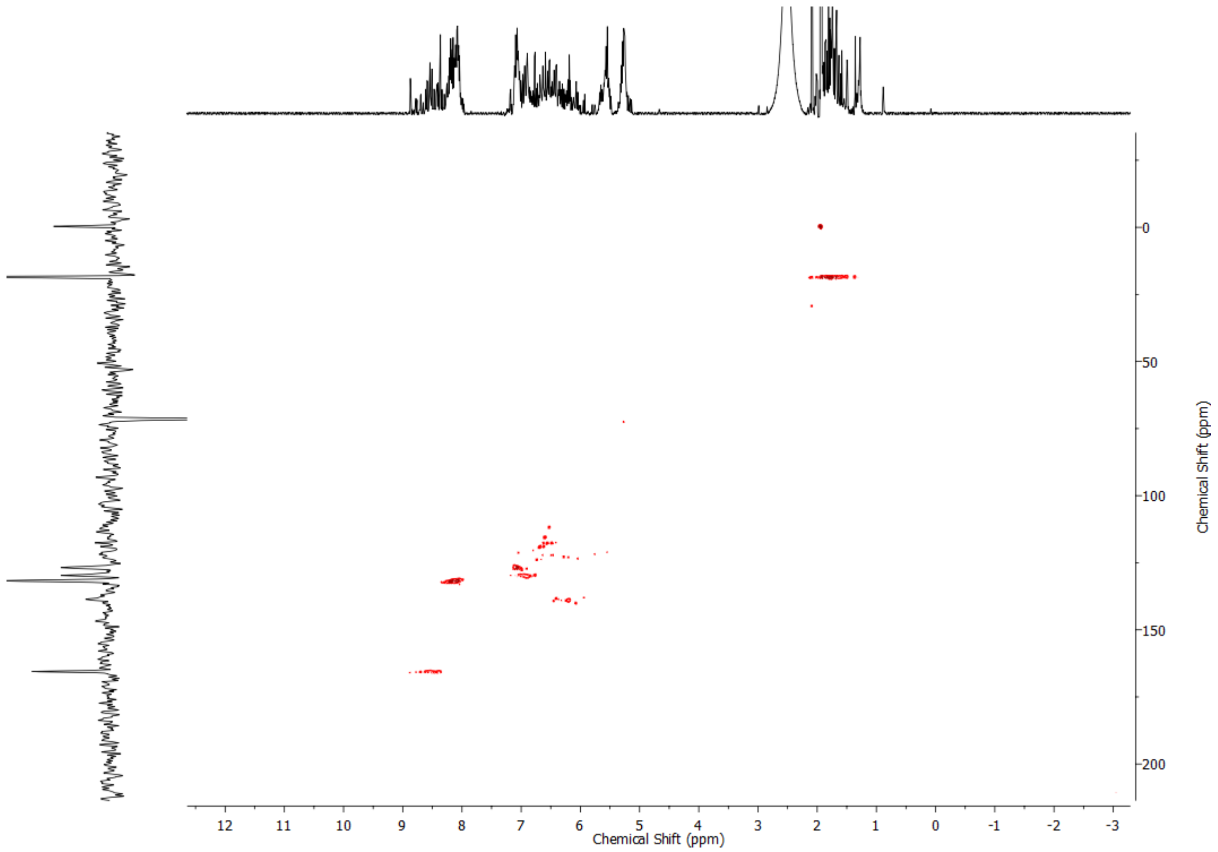

Figure S15. ^{1}H-^{13}C HSQC spectrum (500 MHz, CD_3CN, 298 K) of **1**.

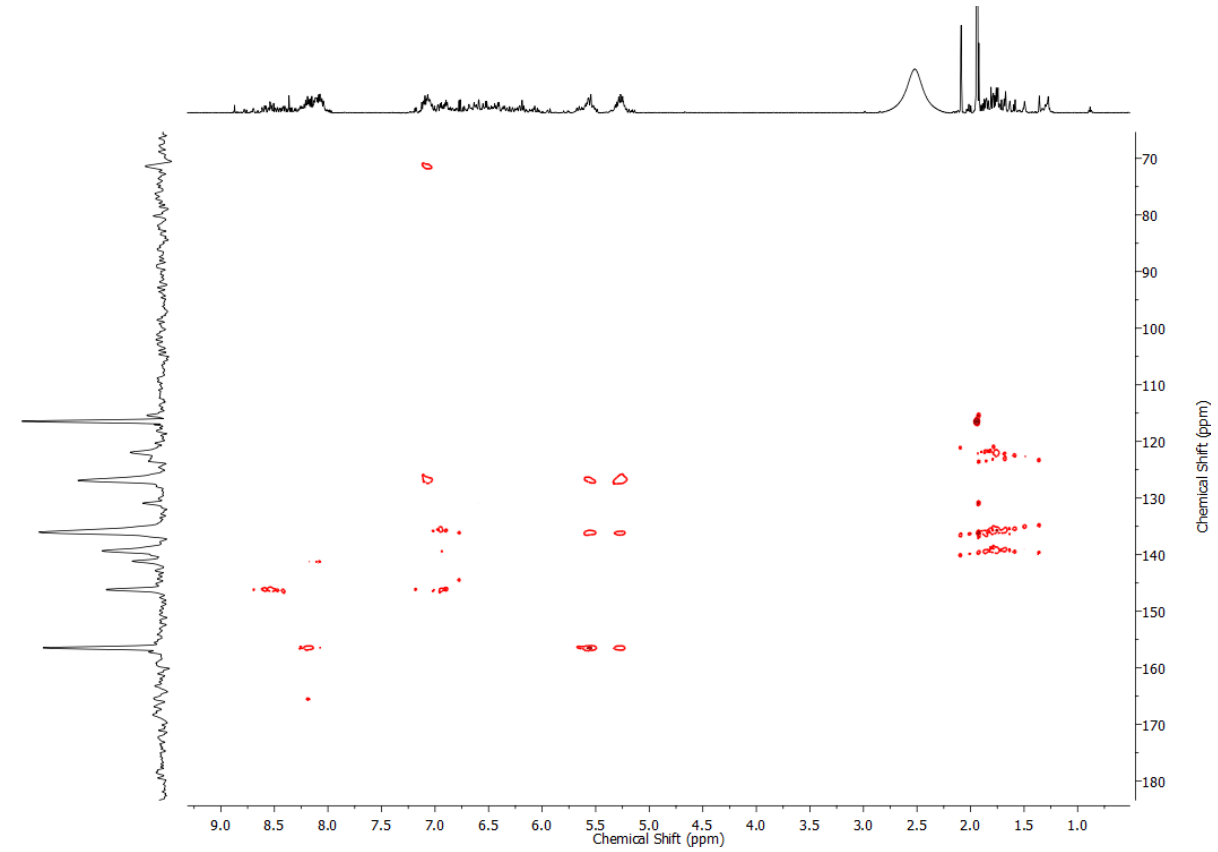

Figure S16. ^{1}H-^{13}C HMBC spectrum (500 MHz, CD_3CN, 298 K) of **1**.

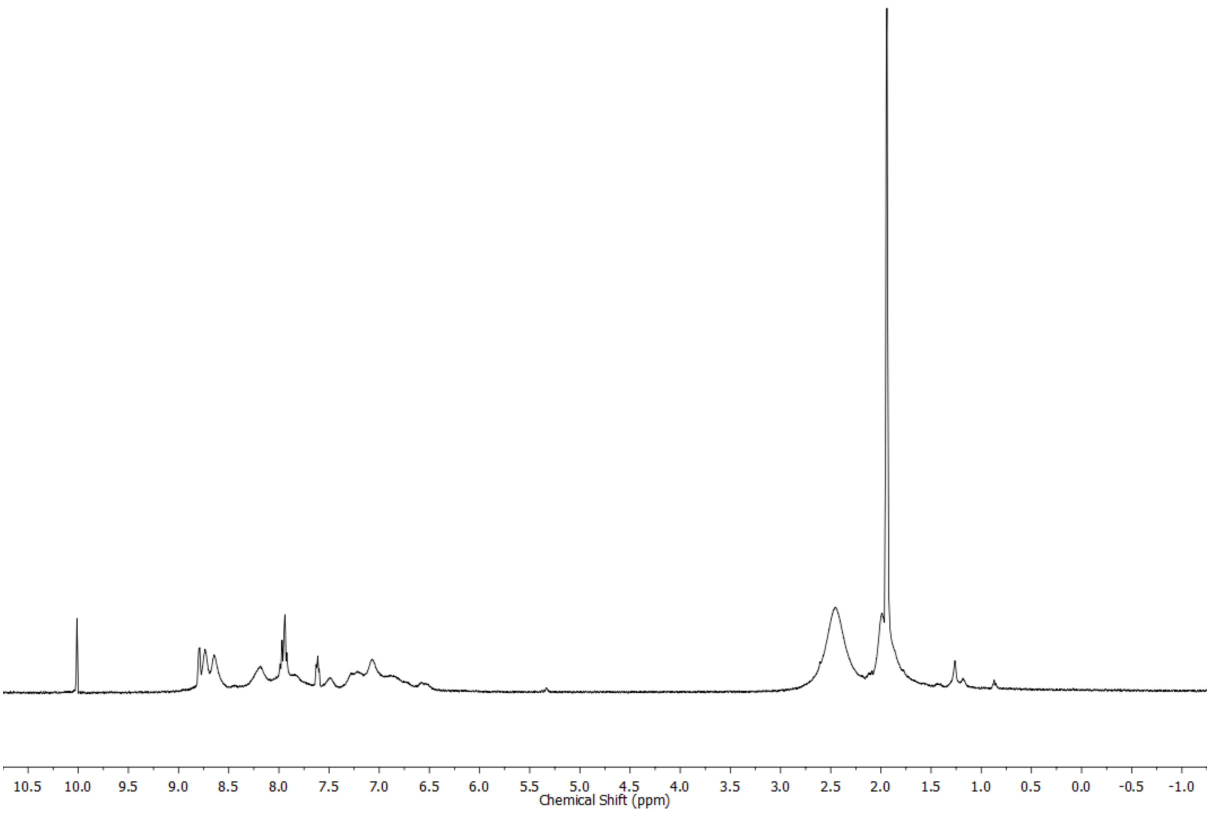

Figure S17. ^{1}H NMR spectrum (400 MHz, CD_3CN, 298 K) obtained from mixing **B**, $Mg(NTf_2)_2$, and 2-formylpyridine. No discrete cage was observed by ^{1}H NMR. No cage peaks were observed in the mass spectrum.

S3.1.2 Synthesis and characterization of 1'

ESI-HR-MS ([**1'**$(NTf_2)_8$] = $C_{192}H_{156}N_{24}O_{12}Zn_4(C_2F_6NO_4S_2)_8$) *m/z* = 406.4950 $[M]^{8+}$ (calc. 406.4932), 504.6963 [**1'**$+NTf_2]^{7+}$ (calc. 504.6946), 635.4661 [**1'**$+2NTf_2]^{6+}$ (calc. 635.4634), 818.5421 [**1'**$+3NTf_2]^{5+}$ (calc. 818.5396), 1093.1566 [**1'**$+4NTf_2]^{4+}$ (calc. 1093.1538), 1550.8440 [**1'**$+5NTf_2]^{3+}$ (calc. 1550.8446).

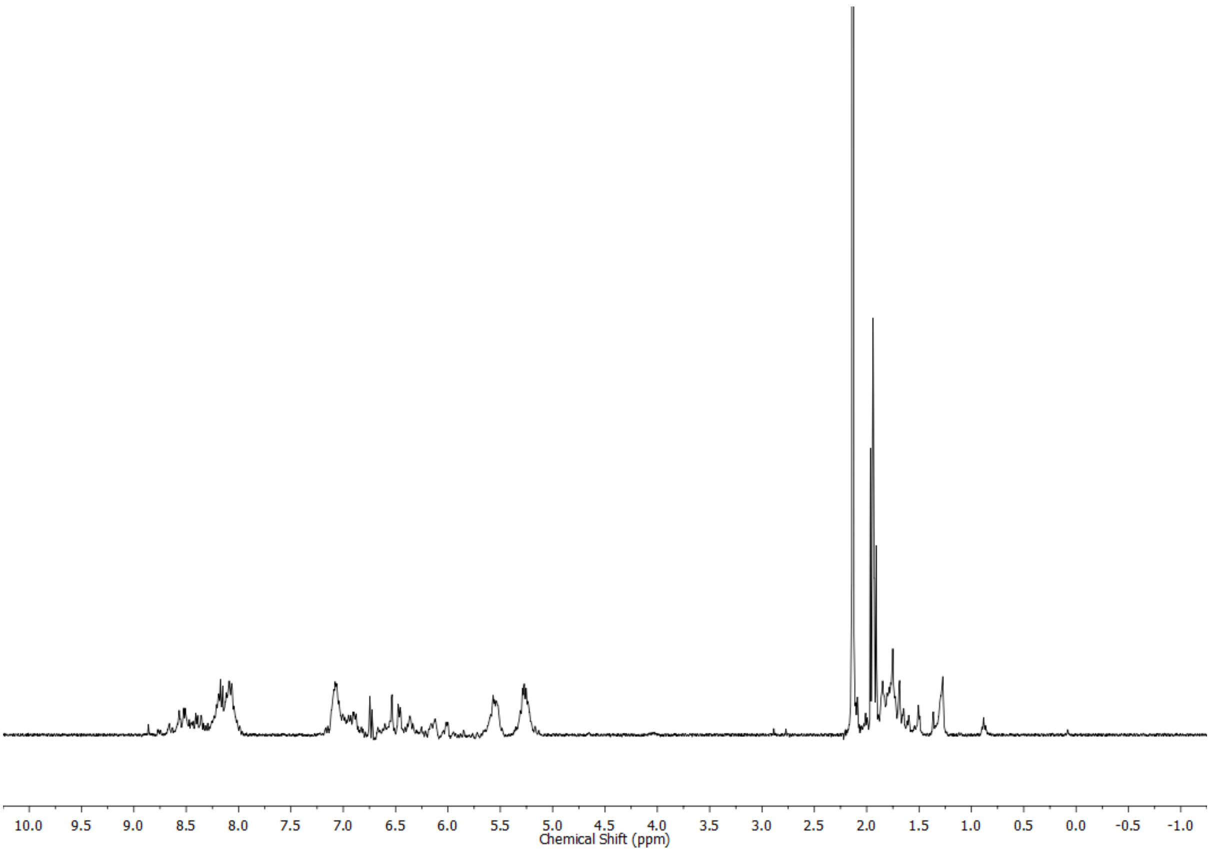

Figure S18. ^{1}H NMR spectrum (400 MHz, CD_3CN, 298 K) of **1'**.

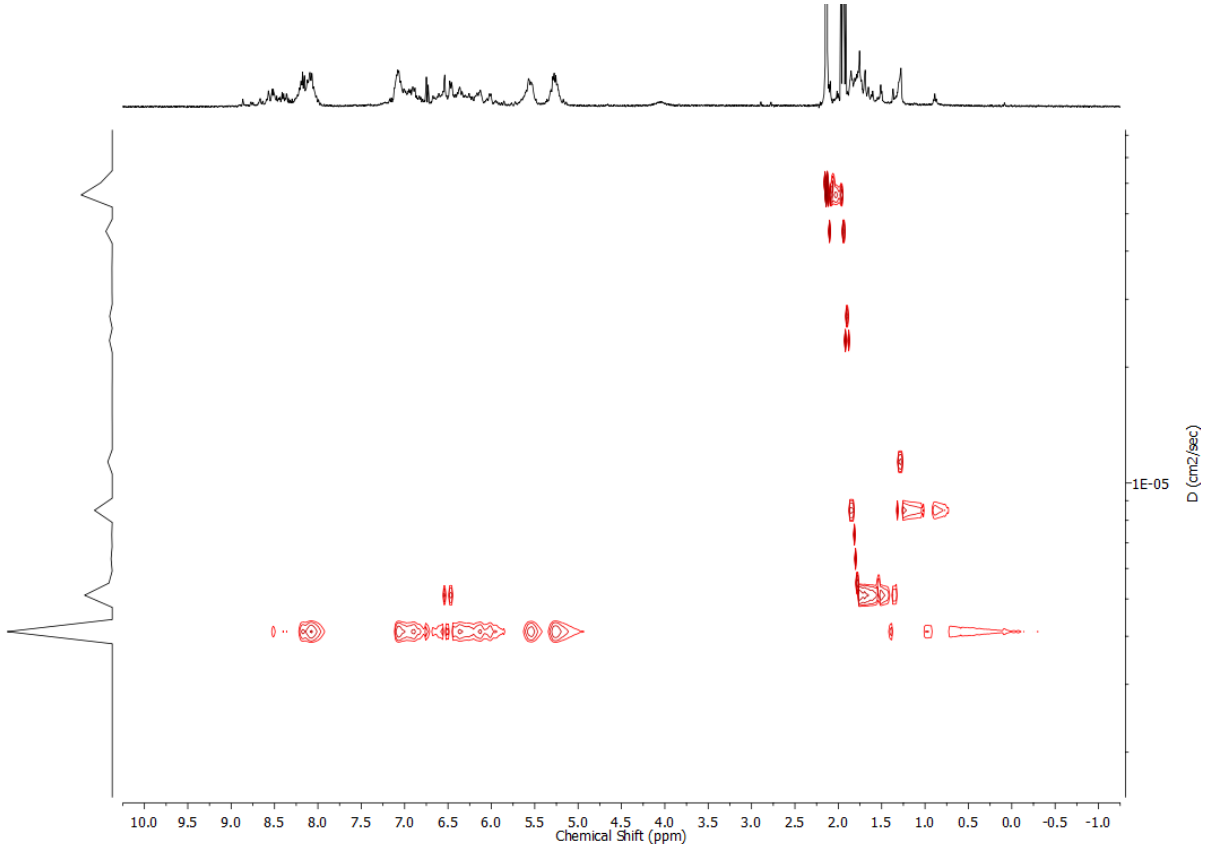

Figure S19. ^{1}H DOSY spectrum (400 MHz, CD_3CN, 298 K) of **1'**.

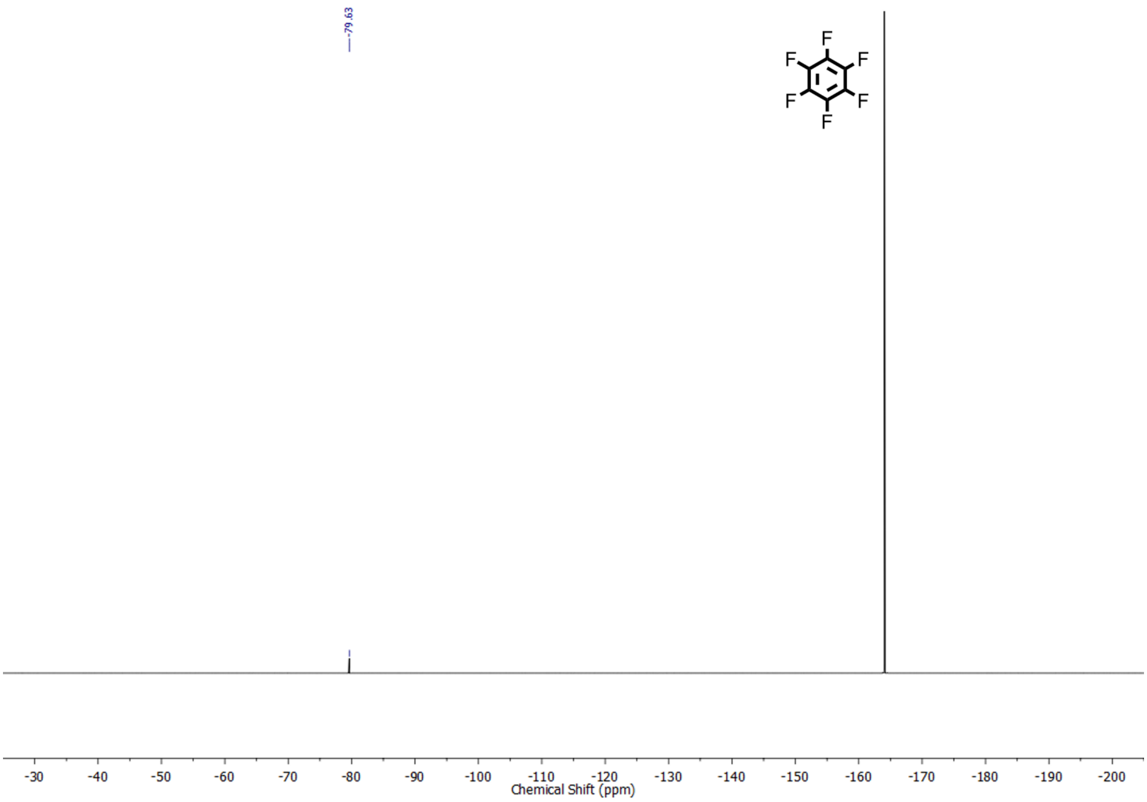

Figure S20. ^{19}F NMR spectrum (376 MHz, CD_3CN, 298 K) of **1'**. Referenced to hexafluorobenzene.

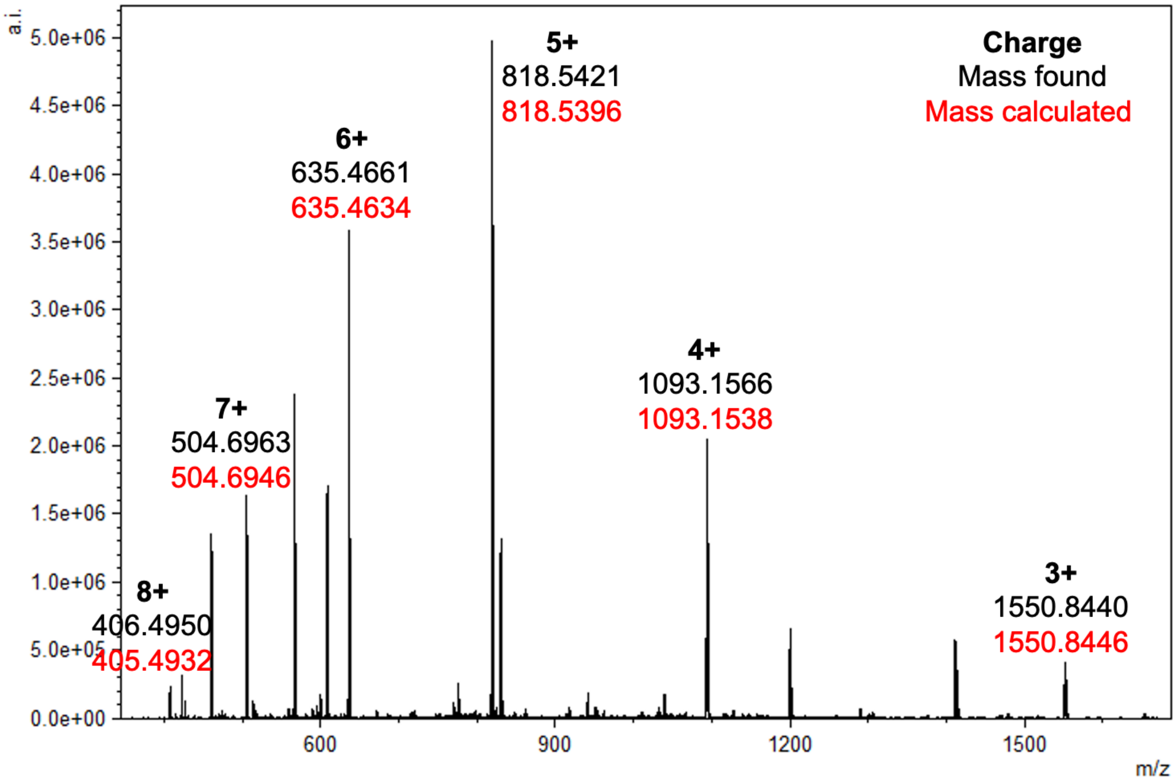

Figure S21. High resolution ESI-mass spectrum of **1'**.

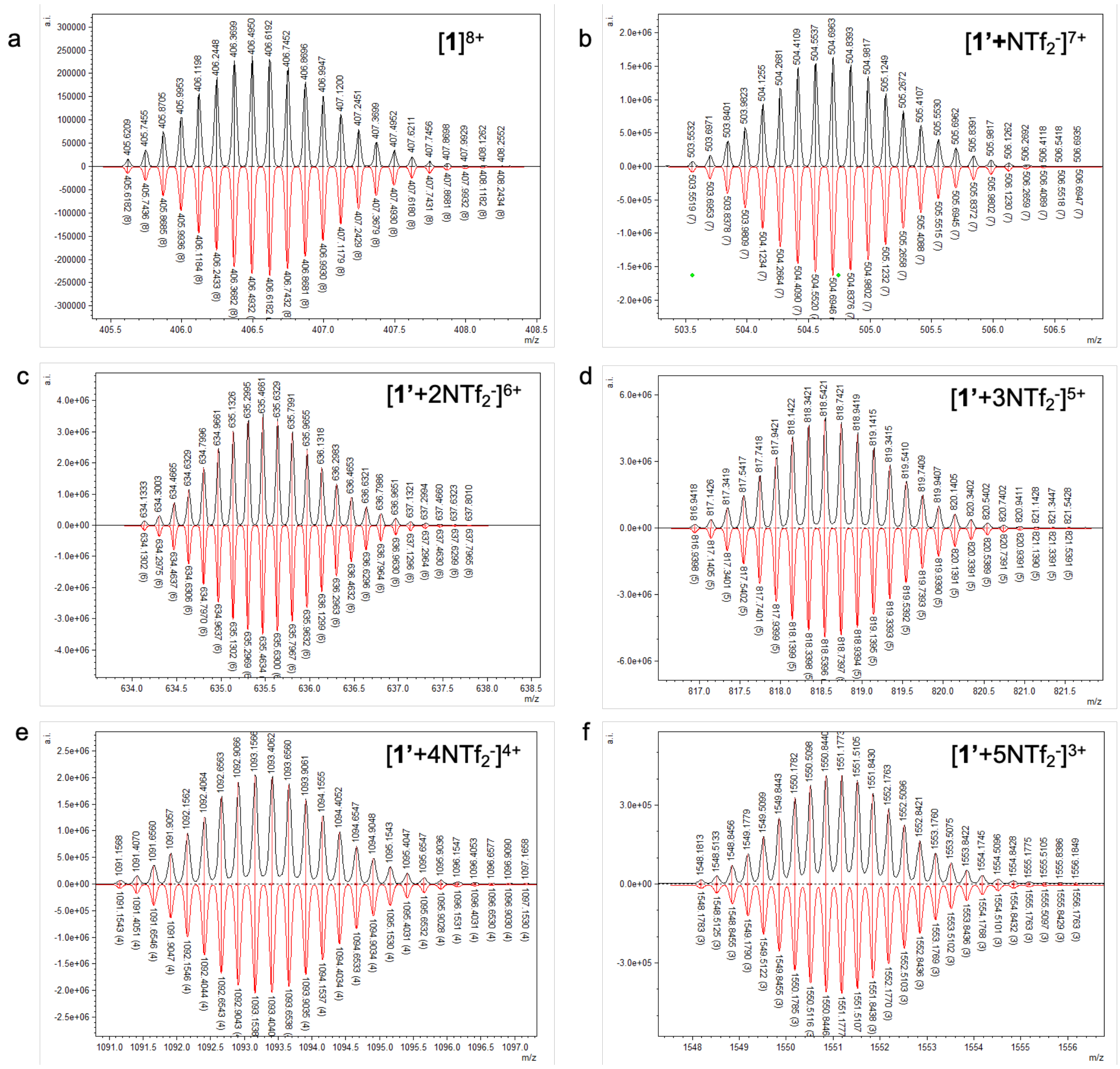

Figure S22. Signals from the HR-ESI-MS spectrum of **1'**. Found and calculated signals for (a) [**1'**]$^{8+}$; (b) [**1'**+NTf_2^-]$^{7+}$; (c) [**1'**+2NTf_2^-]$^{6+}$; (d) [**1'**+3NTf_2^-]$^{5+}$; (e) [**1'**+4NTf_2^-]$^{4+}$; (f) [**1'**+5NTf_2^-]$^{3+}$.

S3.2 Synthesis and characterization of **2** and **2’**

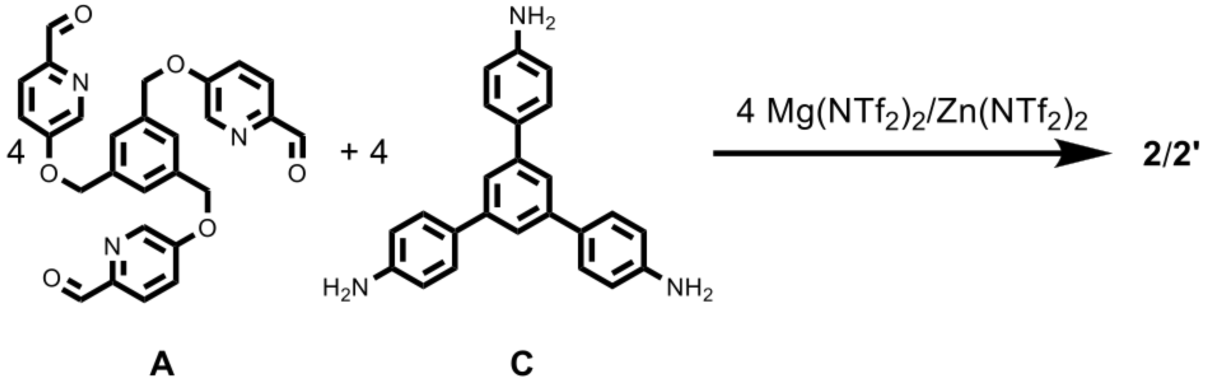

Scheme S5. Subcomponent self-assembly of **2** and **2’**.

The mixture of subcomponent **A** (5.80 mg, 12 μmol, 1 eq.), tri-aniline **C** (4.22 mg, 12 μmol, 1 eq.), and $Mg(NTf_2)_2$ (7.01 mg, 12 μmol, 1 eq.) or $Zn(NTf_2)_2$ (7.51 mg, 12 μmol, 1 eq.), in deuterated acetonitrile (3 mL) was heated at 70 °C for 24 hours. The reaction mixture was allowed to cool to room temperature and the insoluble by-products were removed by filtration through a glass fiber plug. The filtrate solution was used without further purification.

To obtain solid products, the filtrate was separated in 1 mL aliquots and each sample was diluted with diethyl ether to 15 mL. The resulting precipitate was dried under N_2 flow. The product was obtained as a yellow powder (cage **2**: 11.84 mg, 72 %; cage **2’**: 11.6 mg).

S3.2.1 Characterization of 2

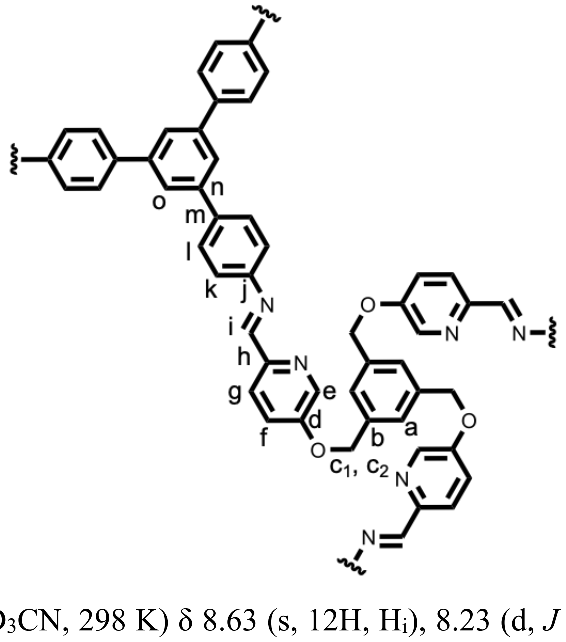

^{1}H NMR (500 MHz, CD_3CN, 298 K) δ 8.63 (s, 12H, H_i), 8.23 (d, J = 8.7 Hz, 12H, H_g), 8.10 (dd, J = 8.6, 2.7 Hz, 12H, H_f), 7.51 (s, 12H, H_o), 7.30 (d, J = 10 Hz, 24H, H_l), 7.13 (s, 12H,

H_a), 6.56 (d, J = 10 Hz, 24H, H_k), 6.35 (d, J = 2.8 Hz, 12H, H_e), 5.63 (d, J = 15 Hz, 12H, H_{c1}), 5.29 (d, J = 15 Hz, 12H, H_{c2}).

^{13}C NMR (125 MHz, CD_3CN, 298 K) δ 165.95 (C_i), 157.06 (C_d), 146.55 (C_j), 141.75 (Ch), 141.41 (C_m), 139.80 (C_n), 139.08 (C_g), 136.89 (C_b), 132.26 (C_e), 131.97 (C_f), 127.86 (C_l), 126.90 (C_a), 124.89 (C_o), 123.39 (NTf_2^-), 120.84 (NTf_2^-), 118.29(NTf_2^-), 115.74 (NTf_2^-), 122.48 (C_k), 71.76 (C_c).

^{19}F NMR (376 MHz, CD_3CN, 298 K) δ -79.65.

ESI-HR-MS ([**2**(NTf_2)$_8$] = $C_{204}H_{144}N_{24}O_{12}Mg_4(C_2F_6NO_4S_2)_8$) *m/z* = 500.0386 [**2**+NTf_2]$^{7+}$ (calc. 499.9999), 630.0226 [**2**+2NTf_2]$^{6+}$ (calc. 629.9860), 812.1979 [**2**+3NTf_2]$^{5+}$ (calc. 812.1670), 1085.2089 [**2**+4NTf_2]$^{4+}$ (calc. 1085.1881), 1540.2228 [**2**+5NTf_2]$^{3+}$ (calc. 1540.2234).

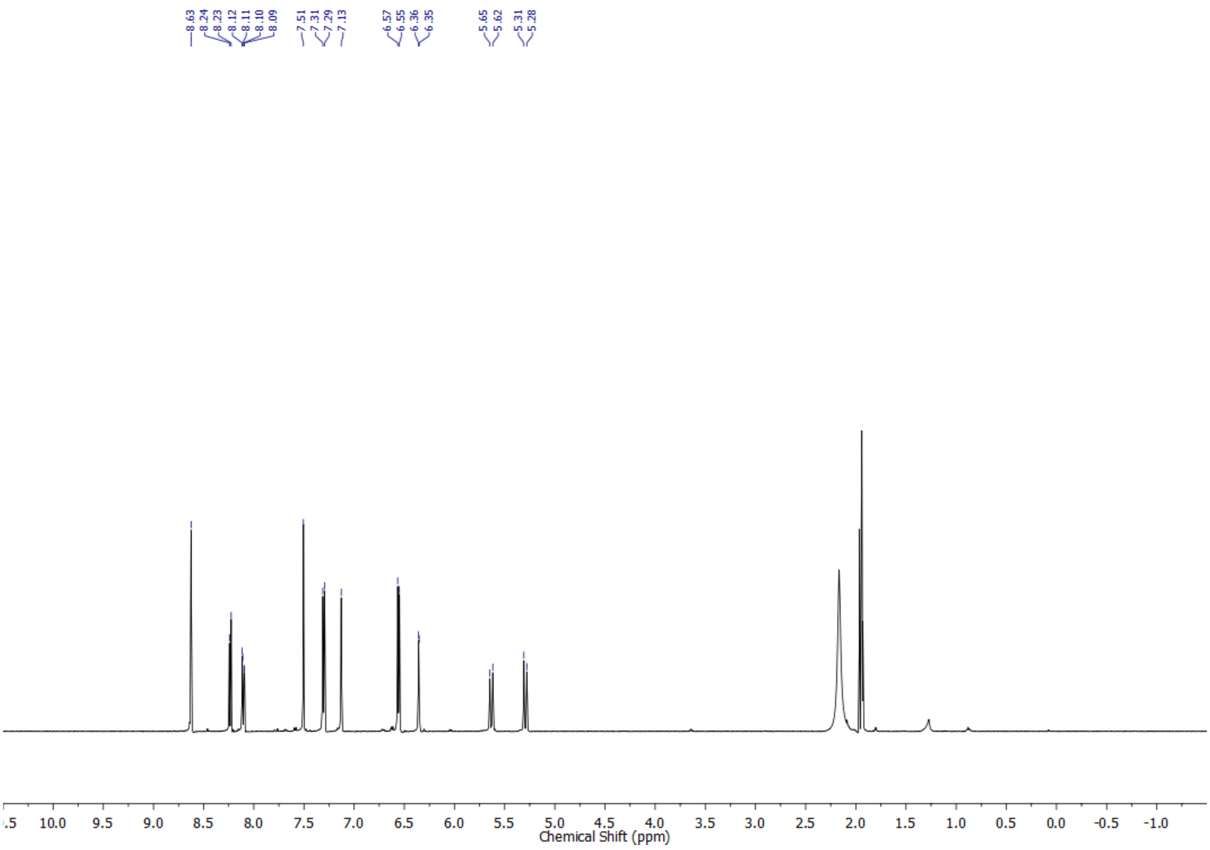

Figure S23. ^{1}H NMR spectrum (500 MHz, CD_3CN, 298 K) of **2**.

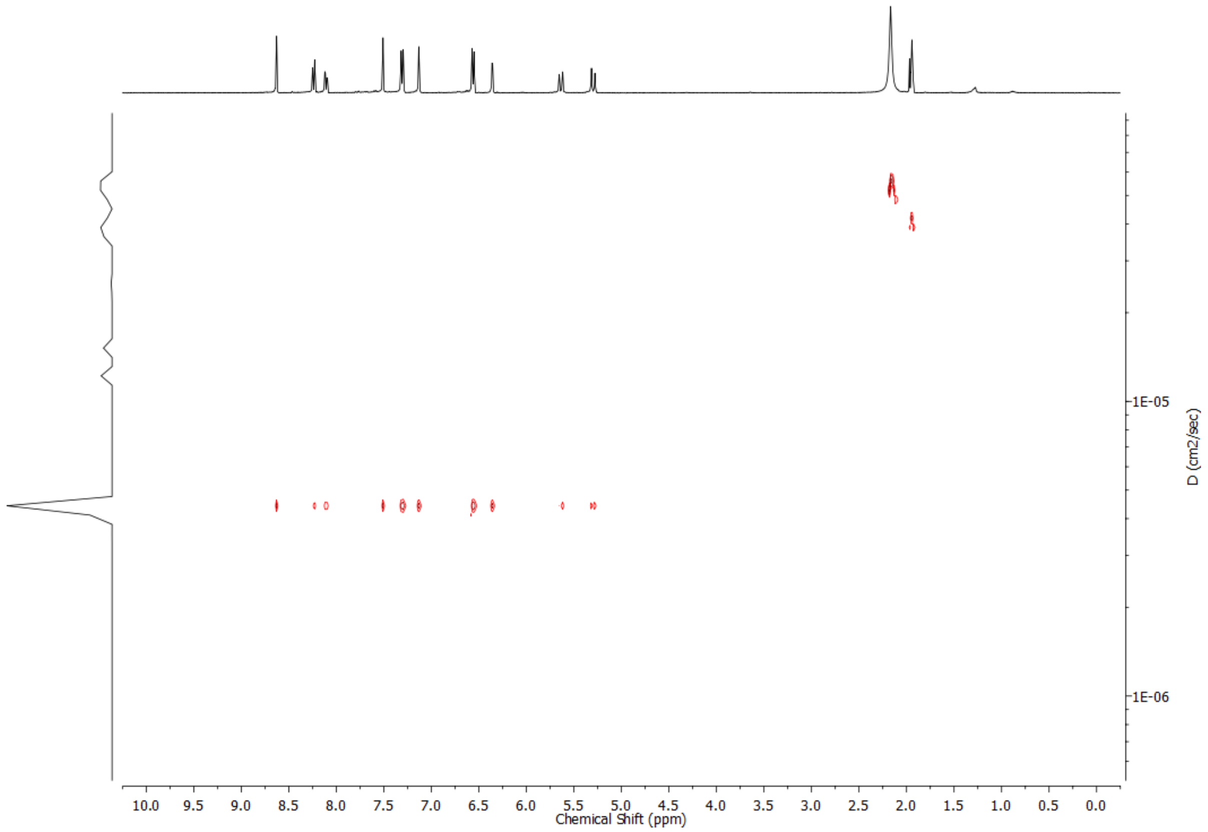

Figure S24. ^{1}H DOSY spectrum (400 MHz, CD_3CN, 298 K) of **2**.

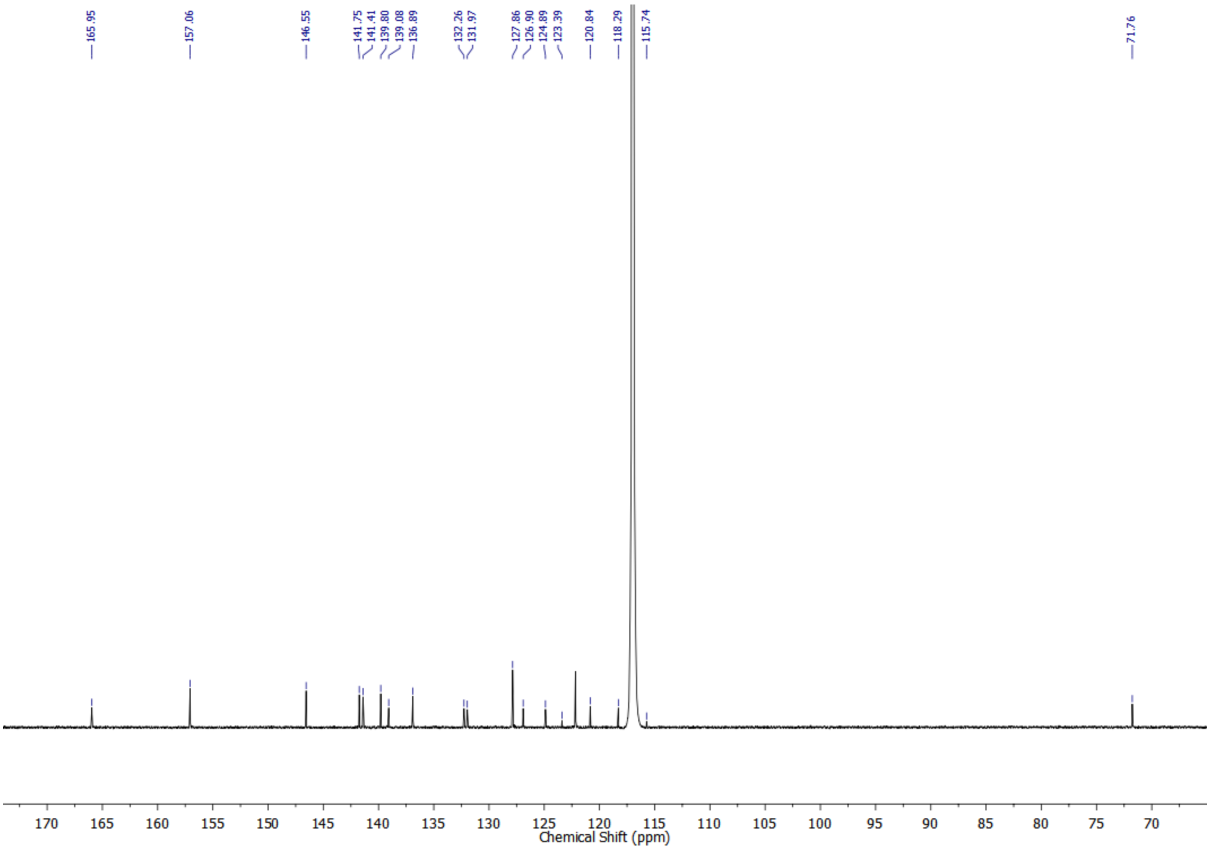

Figure S25. ^{13}C NMR spectrum (125 MHz, CD_3CN, 298 K) of **2**.

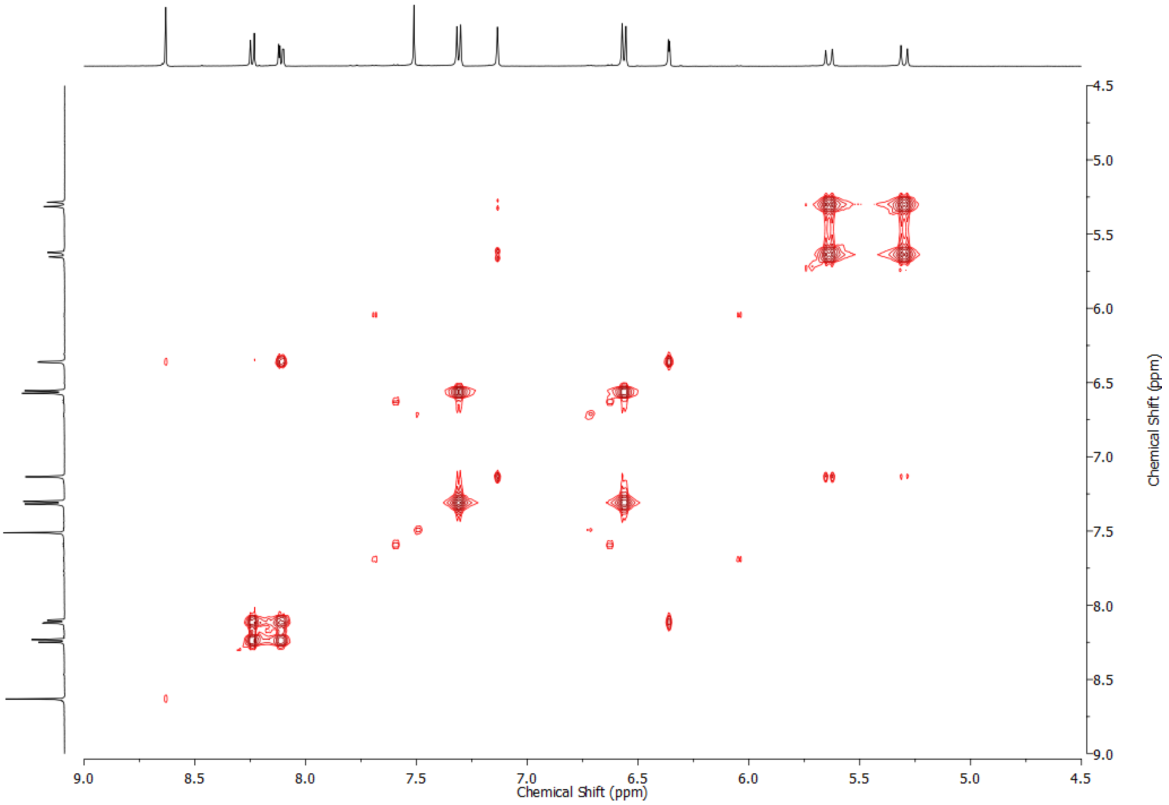

Figure S26. ^{1}H-^{1}H COSY spectrum (500 MHz, CD_3CN, 298 K) of **2**.

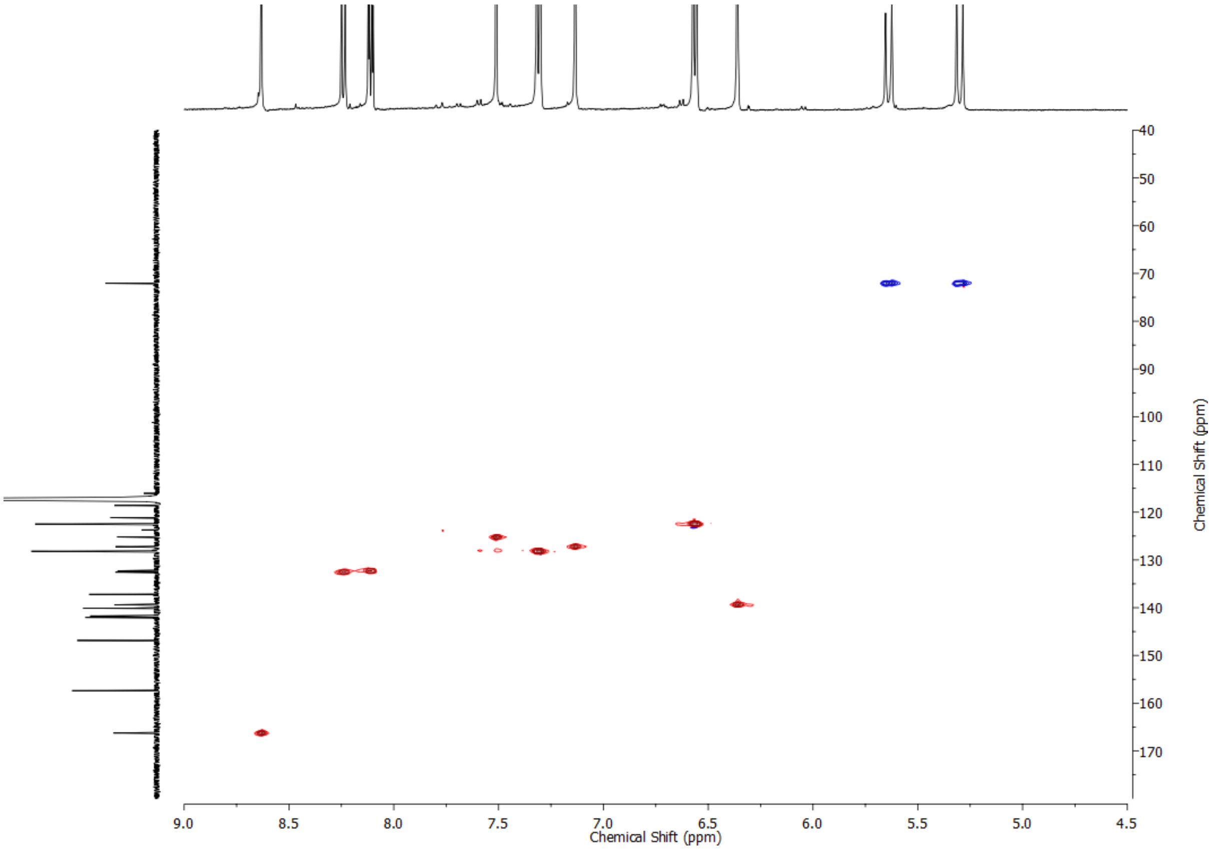

Figure S27. ^{1}H-^{13}C HSQC spectrum (500 MHz, CD_3CN, 298 K) of **2**.

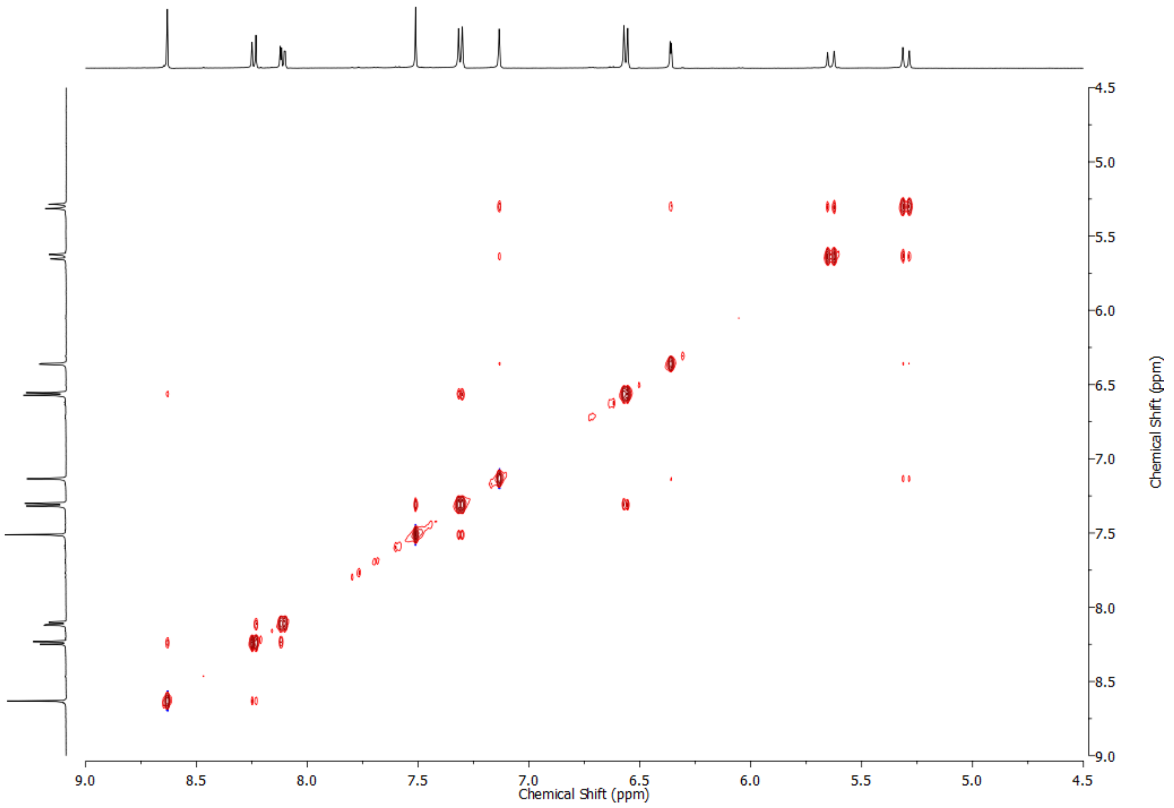

Figure S28. ^{1}H-^{1}H NOESY spectrum (500 MHz, CD_3CN, 298 K) of **2**.

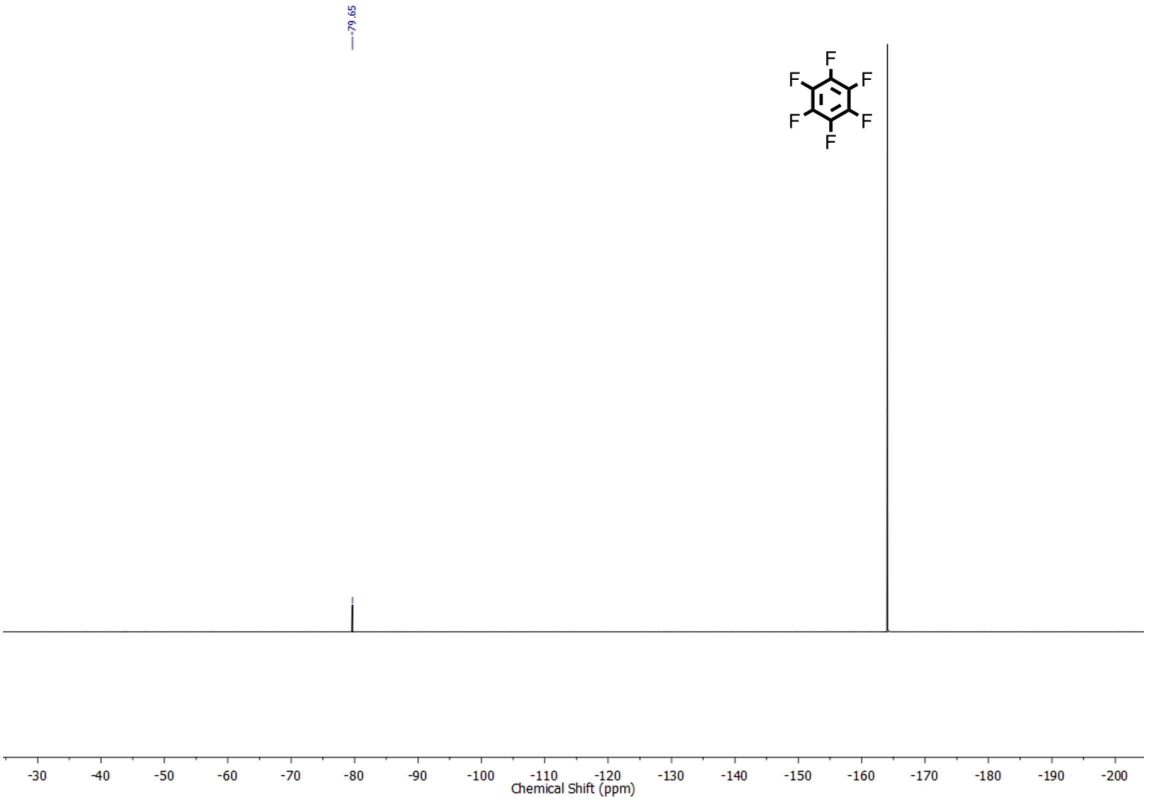

Figure S29. ^{19}F NMR spectrum (376 MHz, CD_3CN, 298 K) of **2**. Referenced to hexafluorobenzene.[3]

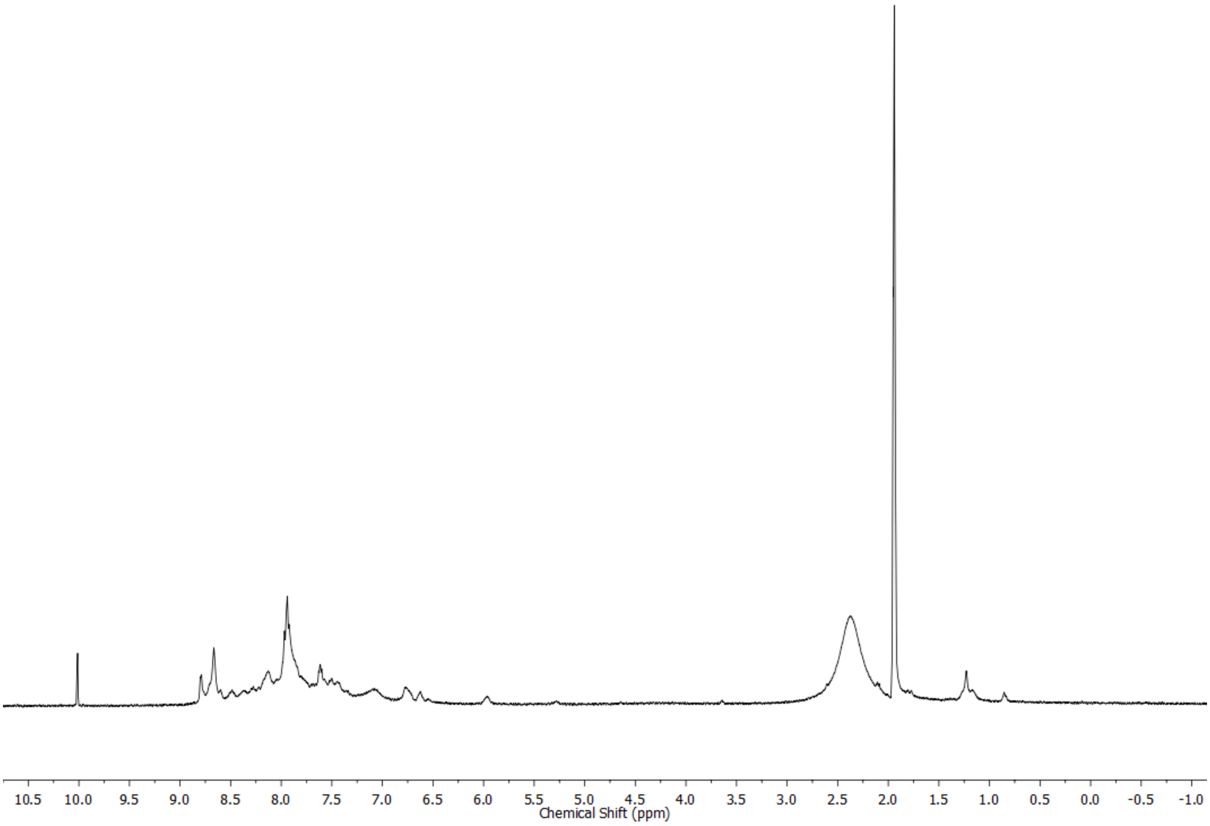

Figure S30. ^{1}H NMR spectrum (400 MHz, CD_3CN, 298 K) obtained from mixing **C**, $Mg(NTf_2)_2$, and 2-formylpyridine. No discrete cage was observed by ^{1}H NMR. No cage peaks were observed in the mass spectrum.

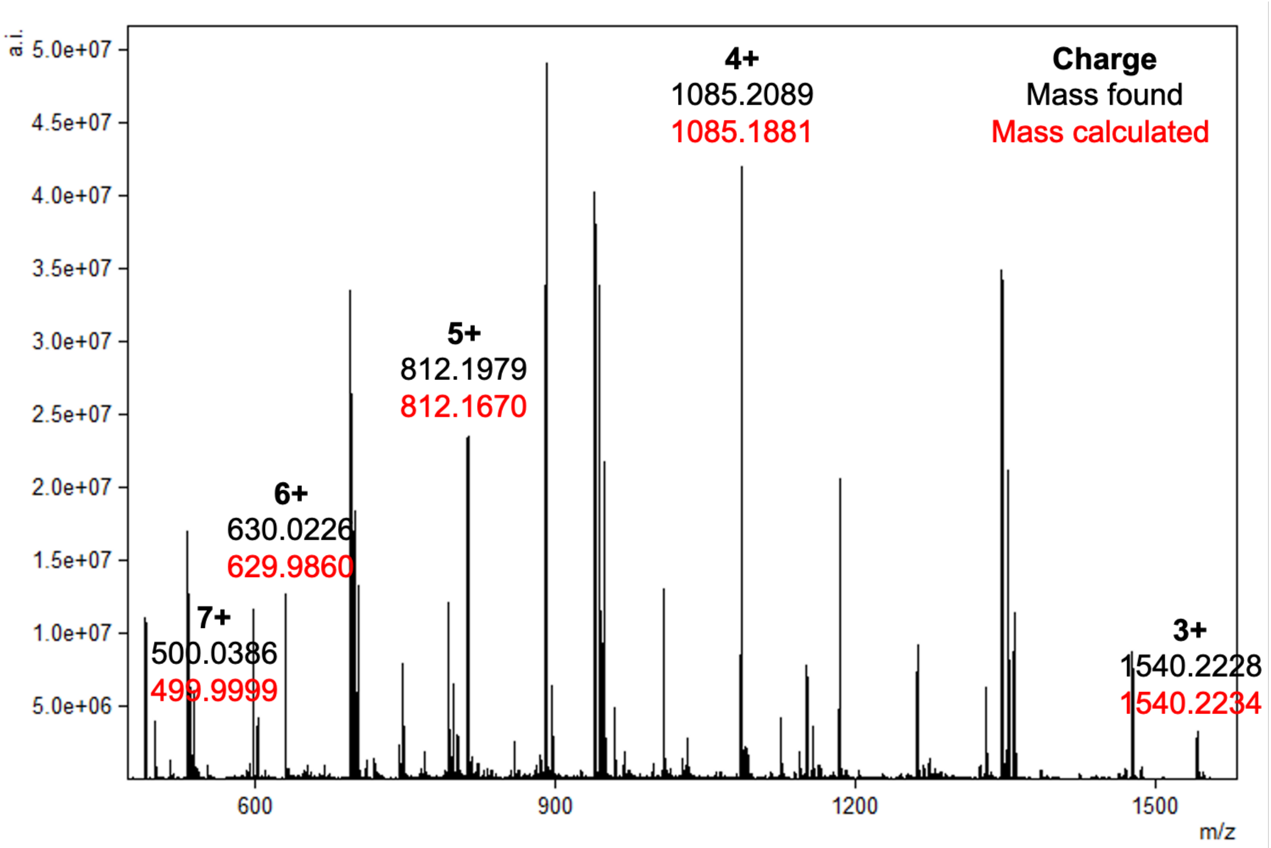

Figure S31. HR-ESI-MS spectrum of cage **2**.

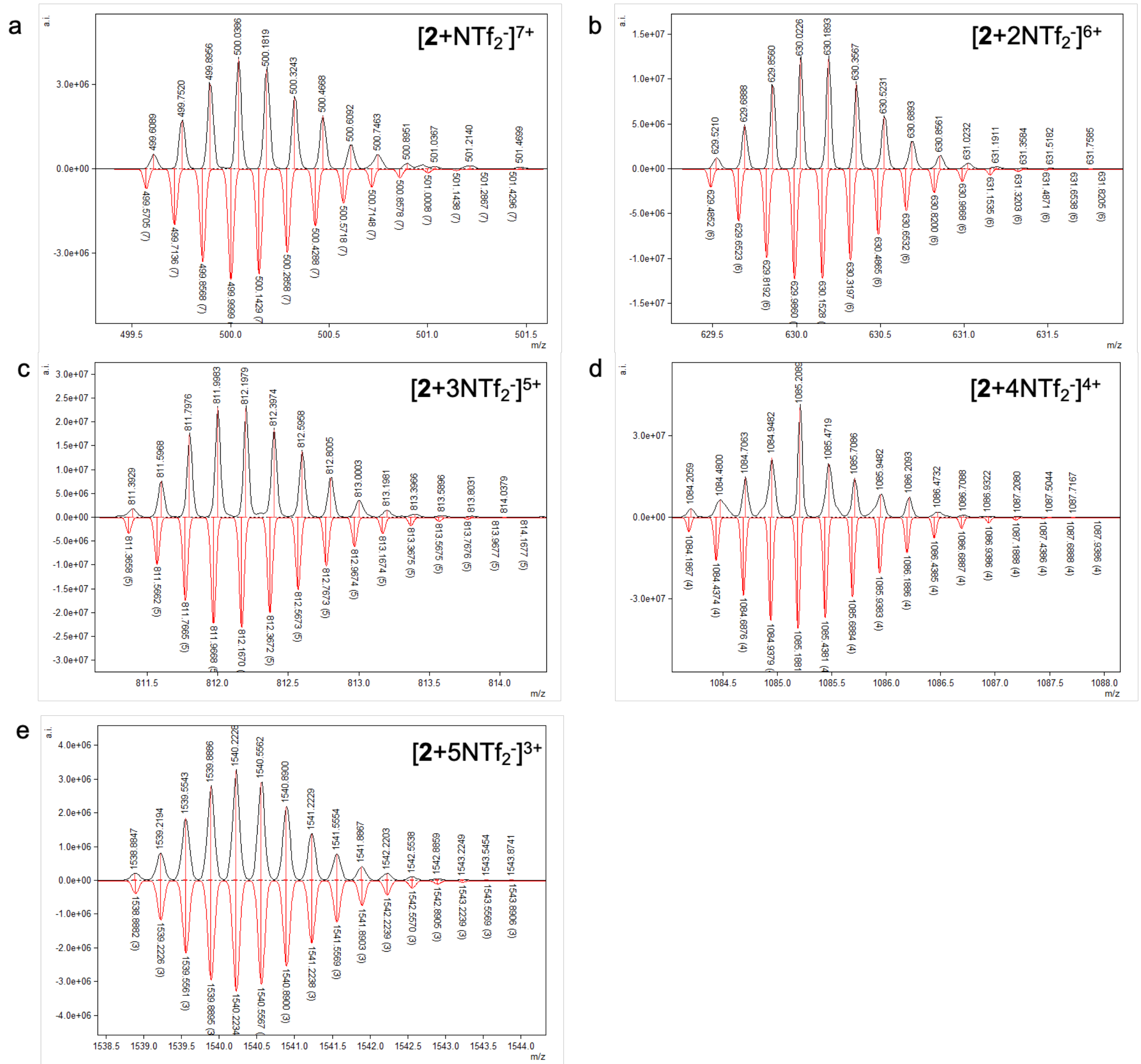

Figure S32. Signals from the HR-ESI-MS spectrum of **2**. Observed and calculated signals for (a) [**2**+NTf_2^-]$^{7+}$; (b) [**2**+2NTf_2^-]$^{6+}$; (c) [**2**+3NTf_2^-]$^{5+}$; (d) [**2**+4NTf_2^-]$^{4+}$; (e) [**2**+5NTf_2^-]$^{3+}$.

S3.2.2 Synthesis and characterization of 2’

2’ tetrahedron:

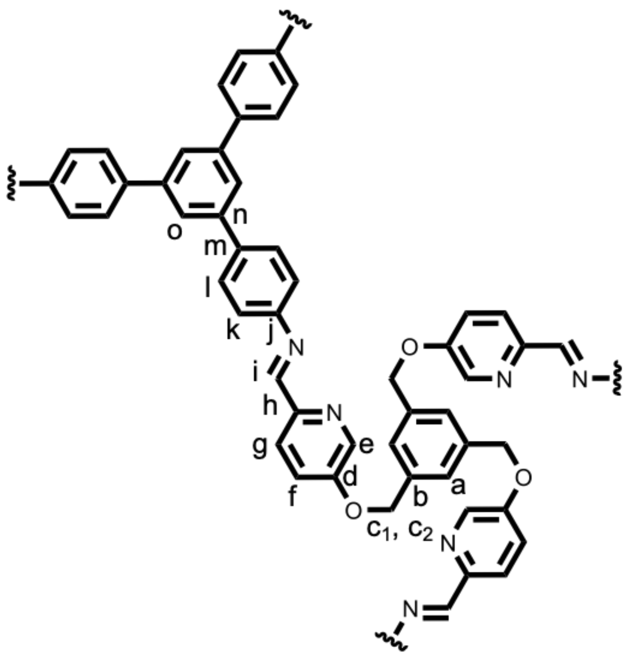

2' Helicate (2'H):

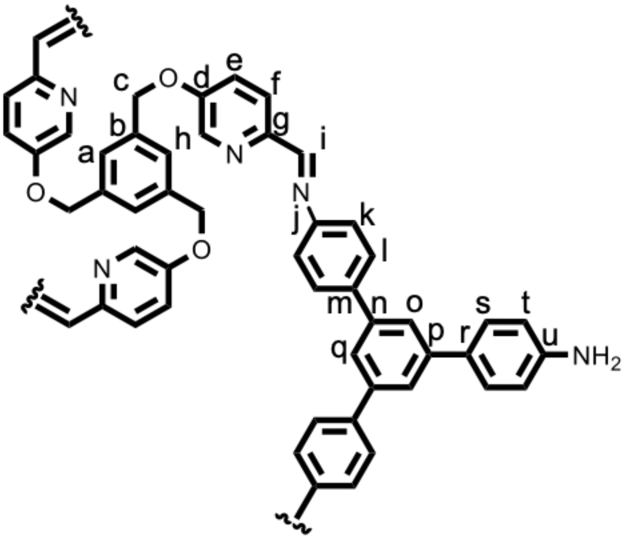

^{1}H NMR (500 MHz, CD_3CN, 298 K) **2'** (tetrahedron): δ 8.64 (s, 3H, H_i), 8.62 (s, 9H, H_i), 8.25 (d, J = 10 Hz, 9H, H_g), 8.22 (d, J = 10 Hz, 3H, H_g), 8.11 (m, 12H, H_f), 7.60 (d, J = 10 Hz, 6H, H_l), 7.49 (s, 12H, H_o), 7.27 (m, 18 H, H_l), 7.12 (s, 12H, H_a), 6.56 (d, J = 5 Hz, 6H, H_k), 6.49 (d, J = 5 Hz, 18H, H_k), 6.27 (d, J = 5 Hz, 9H, H_e), 6.23 (d, J = 5 Hz, 3H, H_e), 5.63 (m, 12H, H_{c1}/H_{c2}), 5.30 (m, 12 H, H_{c1}/H_{c2}).

2'H (helicate): δ 8.47 (s, 6H, H_i), 8.18 (d, J = 10 Hz, 6H, H_f), 8.11–8.09 (m, 6H, H_e), 7.81 (d, J = 1.8 Hz, 6H, H_o), 7.75 (t, J = 1.8 Hz, 3H, H_q), 7.69 (d, J = 10 Hz, 12H, H_l), 7.50 (m, 6H, H_s/H_t), 7.16 (s, 6H, H_a), 6.71 (m, 6H, H_s/H_t), 5.95 (d, J = 10 Hz, 12H, H_k), 5.73 (d, J = 15 Hz, 6 H, H_{c1}), 5.30 (m, 6H, H_{c2}). Some peaks cannot be assigned because of signal overlap.

^{13}**C NMR** (125 MHz, CD_3CN, 298 K) **2’** (tetrahedron): δ 163.26 (C_i), 163.12 (C_i), 146.52 (C_h), 146.23 (C_h), 141.54 (C_m), 140.22 (C_j), 140.03 (C_d), 139.92 (C_m), 139.81 (C_j), 139.78 (C_d), 138.98 (C_e), 138.80 (C_e), 132.25 (C_g), 132.01 (C_g), 132.11 (C_f), 132.08 (C_f), 127.76 (C_l), 127.36 (C_l), 127.46 (C_a), 127.12 (C_b), 126.70 (C_a), 125.41 (C_o), 125.03 (C_n), 122.62 (C_k), 122.19 (C_k), 72.00 (C_c), 71.89 (C_c).

2’H (helicate): δ 163.36 (C_i), 157.33 (C_d), 147.95 (C_u), 139.70 (C_j), 139.47 (C_g), 132.41 (C_e), 131.81 (C_f), 126.37 (C_b), 124.31 (C_o), 128.19 (C_p), 128.09 (C_s), 128.05 (C_l), 127.68 (C_r), 123.91 (C_n), 122.59 (C_q), 121.61 (C_k), 114.25 (C_t), 71.45 (C_c).

NTf_2^-: δ 123.38, 120.83, 118.28, 115.73.

^{19}**F NMR** (376 MHz, CD_3CN, 298 K) δ -79.63.

ESI-HR-MS ([**2’**(NTf_2)$_8$] = $C_{204}H_{144}N_{24}O_{12}Zn_4(C_2F_6NO_4S_2)_8$) *m/z* = 423.0980 [**2’**]$^{8+}$ (calc. 423.1065), 523.5281 [**2’**+NTf_2]$^{7+}$ (calc. 523.5385), 657.4337 [**2’**+2NTf_2]$^{6+}$ (calc. 657.4479), 844.9027 [**2’**+3NTf_2]$^{5+}$ (calc. 844.9209), 1126.1062 [**2’**+4NTf_2]$^{4+}$ (calc. 1126.1305), 1595.1084 [**2’**+5NTf_2]$^{3+}$ (calc. 1595.1468).

([**2’H**(NTf_2)$_4$] = $C_{126}H_{93}N_{15}O_6Zn_4(C_2F_6NO_4S_2)_4$) *m/z* = 510.8882 [**2’H**]$^{4+}$ (calc. 510.8999), 774.4865 [**2’H** +NTf_2]$^{3+}$ (calc. 774.5059), 1301.6840 [**2’H** +2NTf_2]$^{2+}$ (calc. 1302.7175).

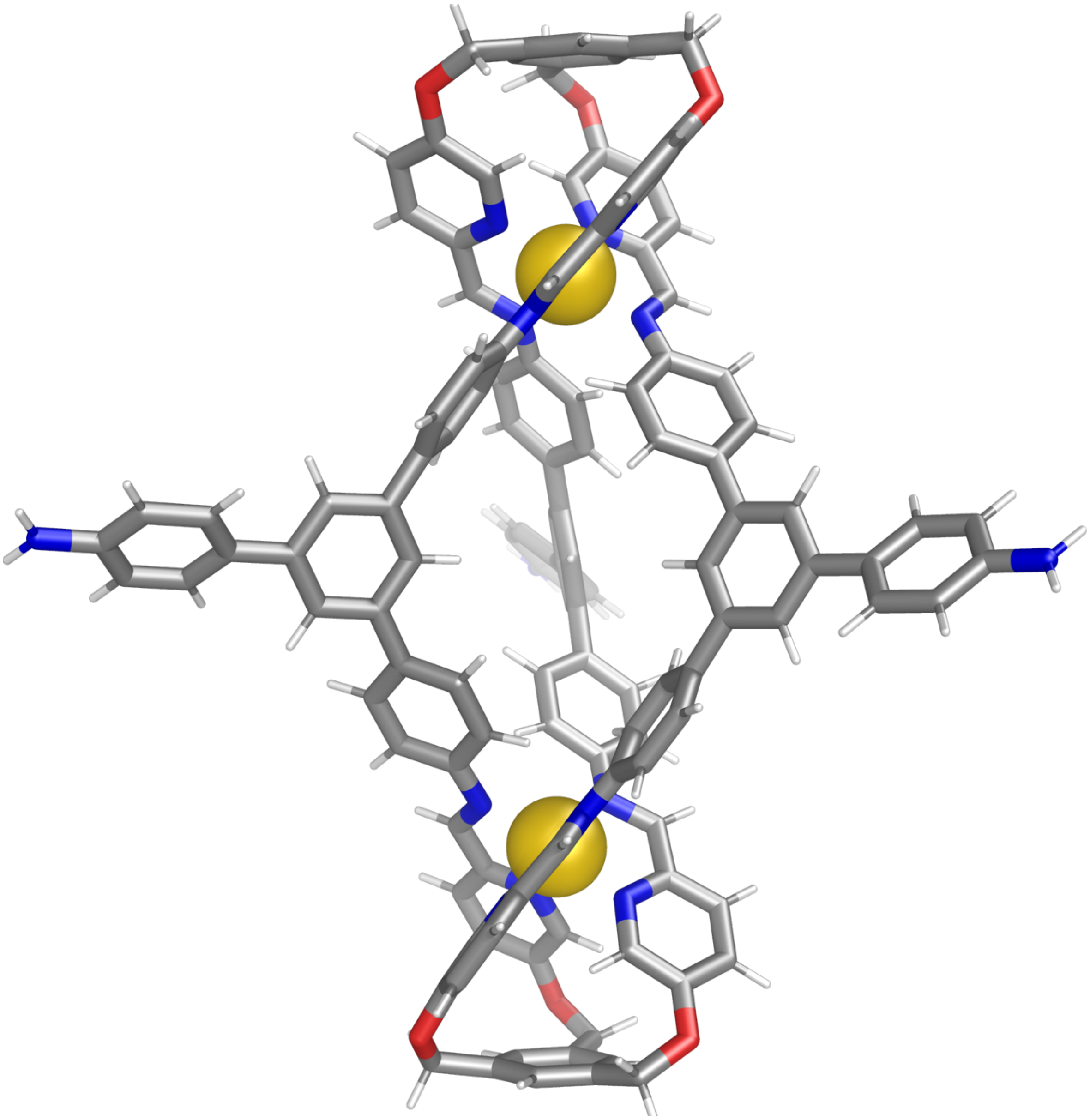

Figure S33. Modelled structure of **2’H**, a helicate formed as a side product alongside the tetrahedron **2’**.

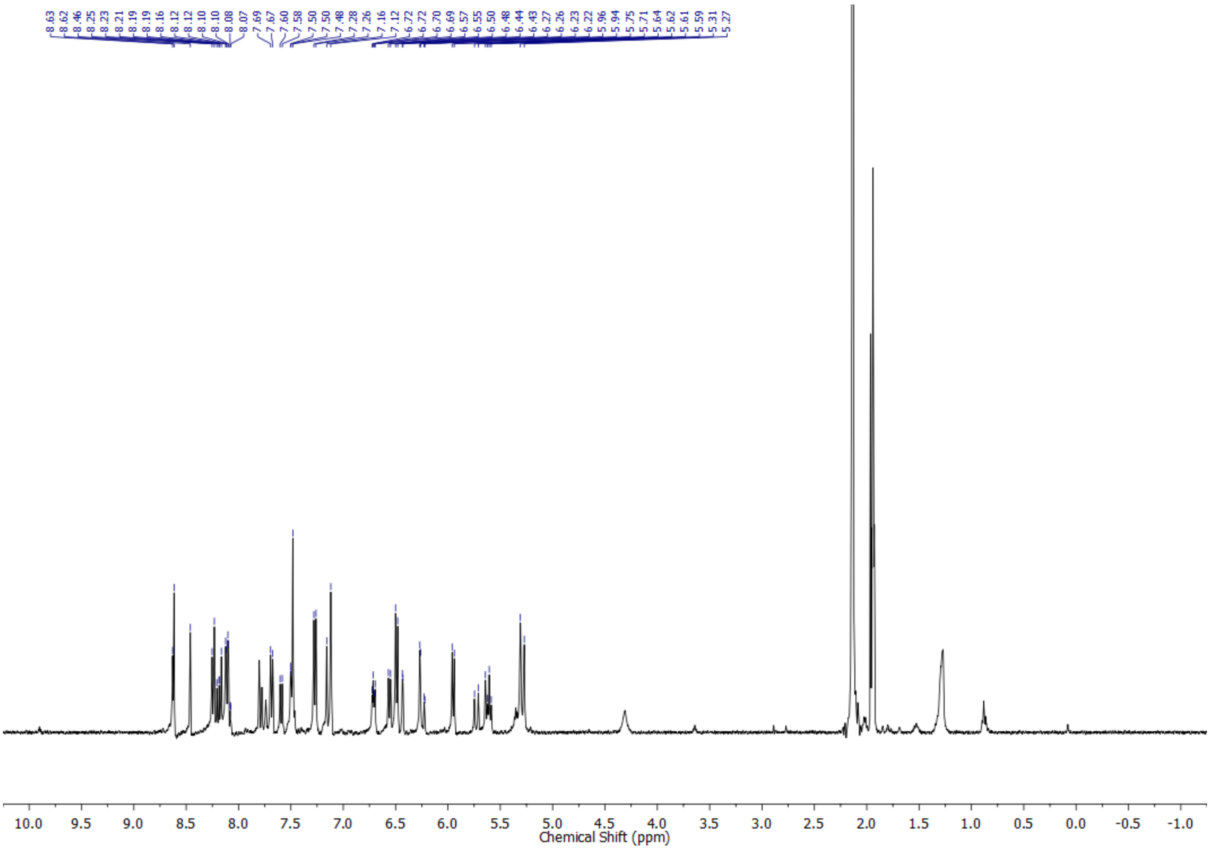

Figure S34. ^{1}H NMR spectrum (500 MHz, CD_3CN, 298 K) of **2’**, showing the presence of two species: a tetrahedron with C_3 symmetry and a helicate, in a 1.2:1 ratio.

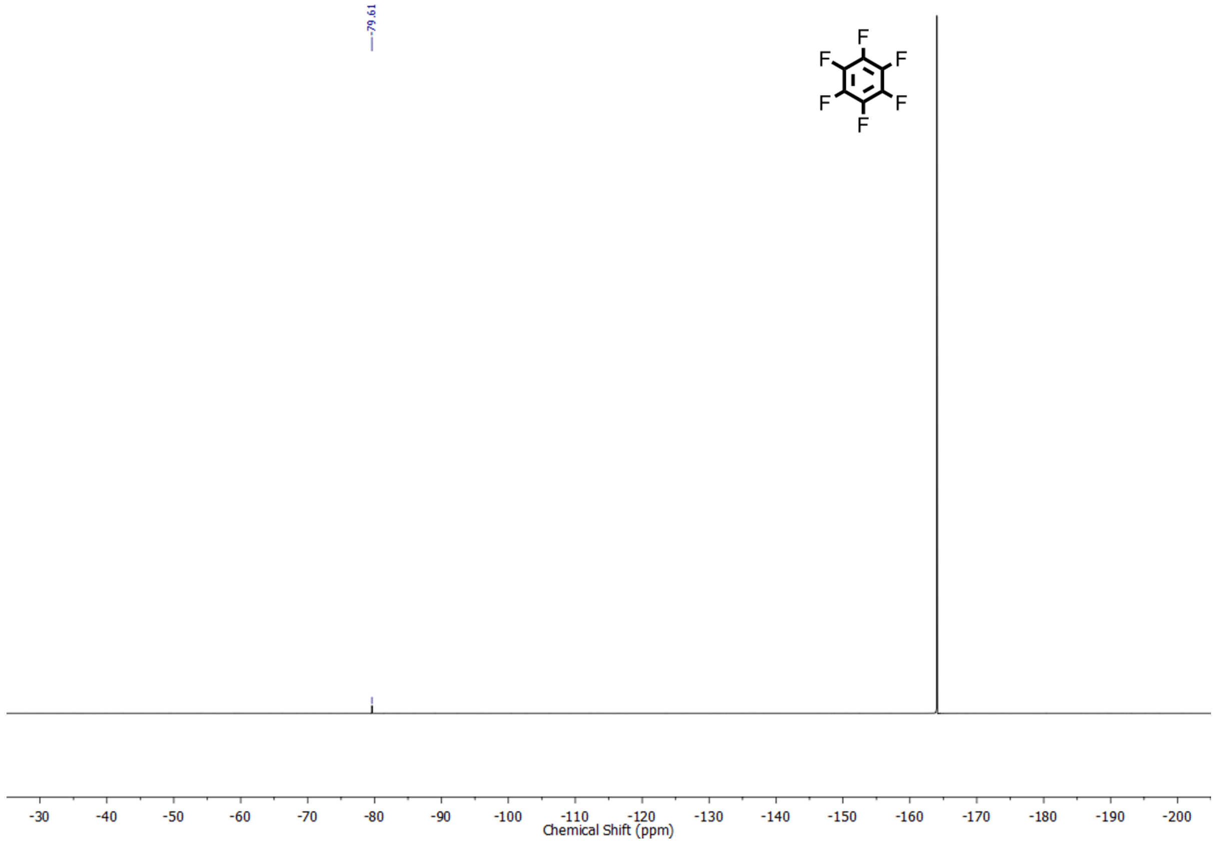

Figure S35. ^{19}F NMR spectrum (376 MHz, CD_3CN, 298 K) of **2'**. Referenced to hexafluorobenzene.

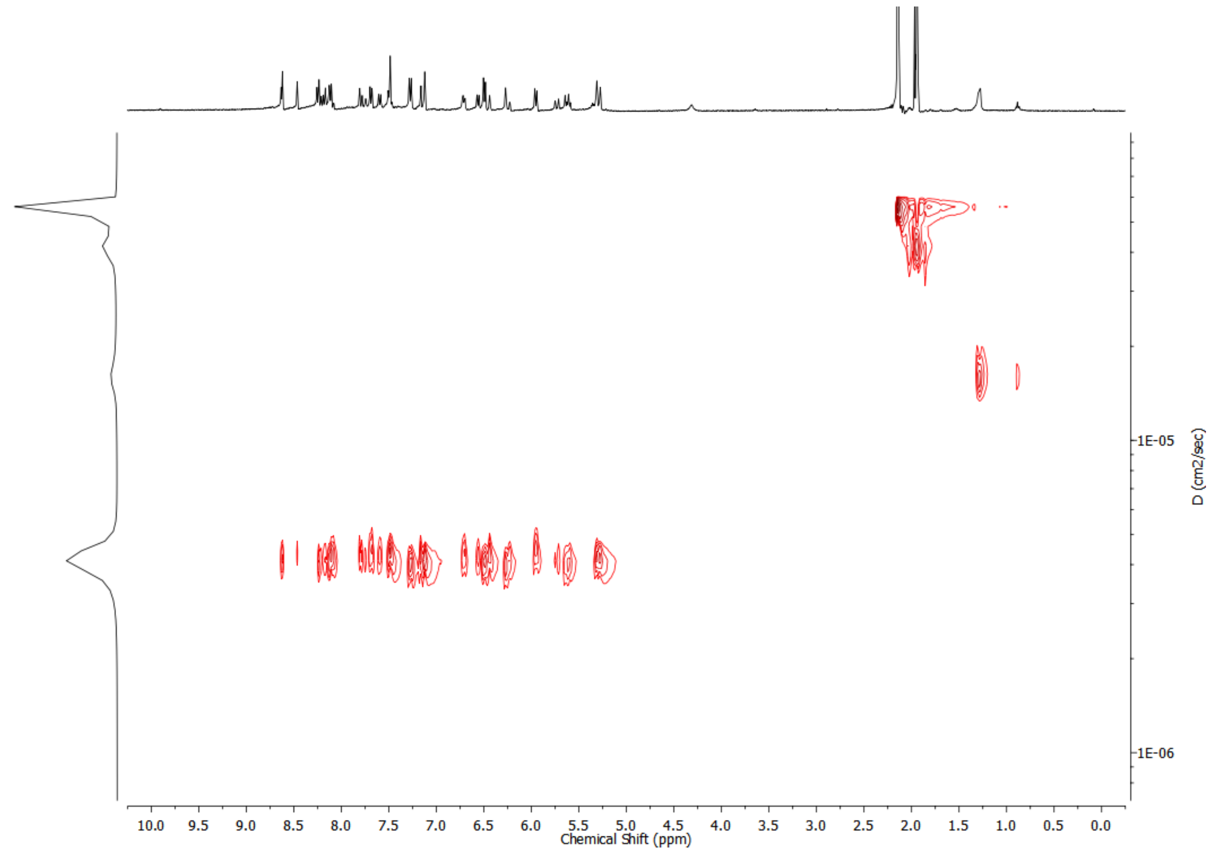

Figure S36. ^{1}H DOSY spectrum (400 MHz, CD_3CN, 298 K) of **2'**.

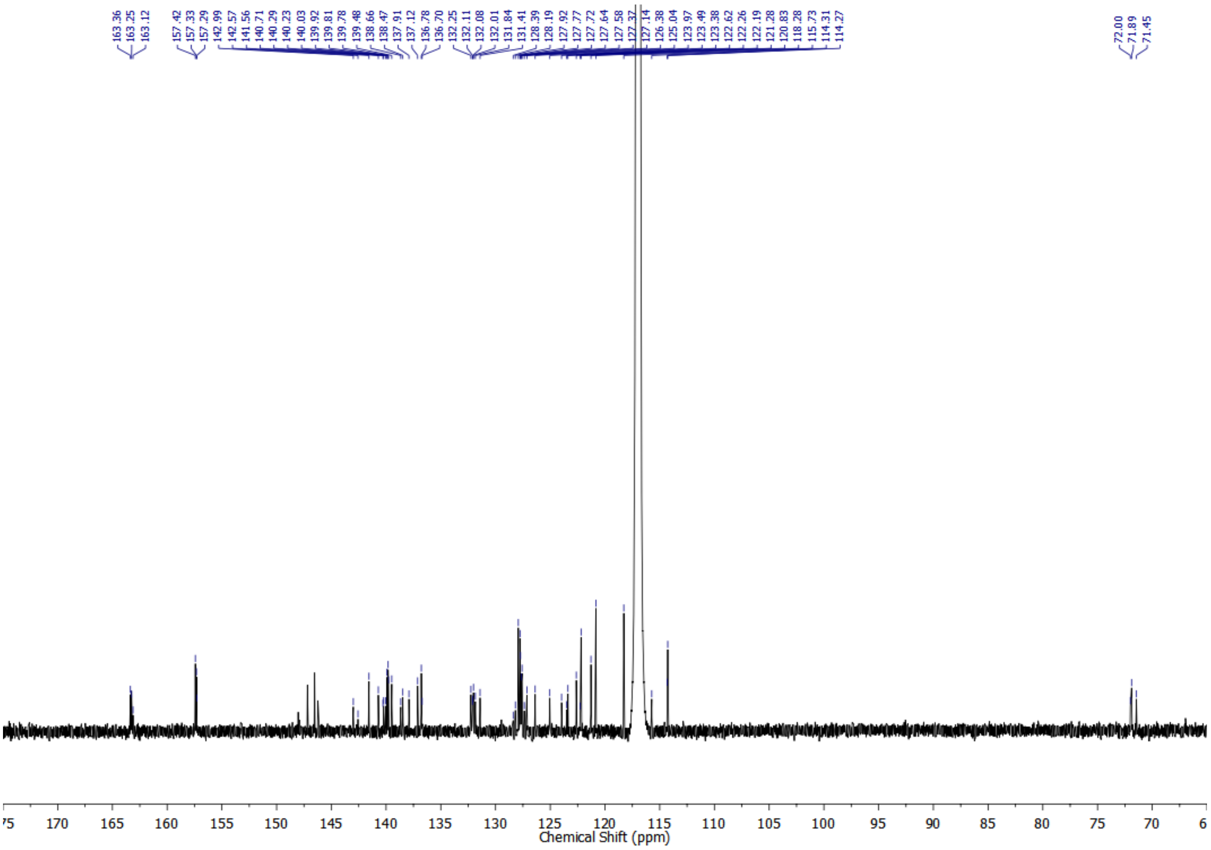

Figure S37. ^{13}C NMR spectrum (125 MHz, CD_3CN, 298 K) of **2'**.

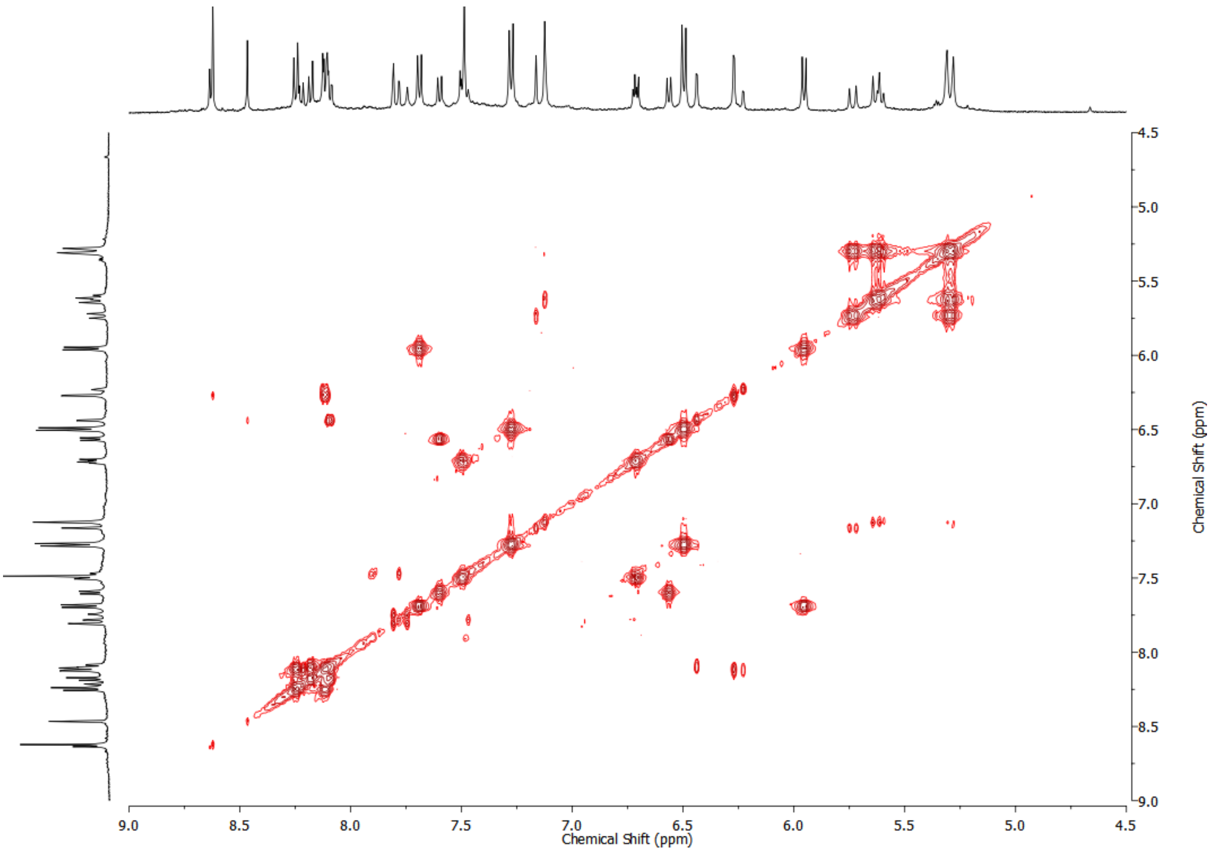

Figure S38. ^1H-^1H COSY spectrum (500 MHz, CD_3CN, 298 K) of **2’**.

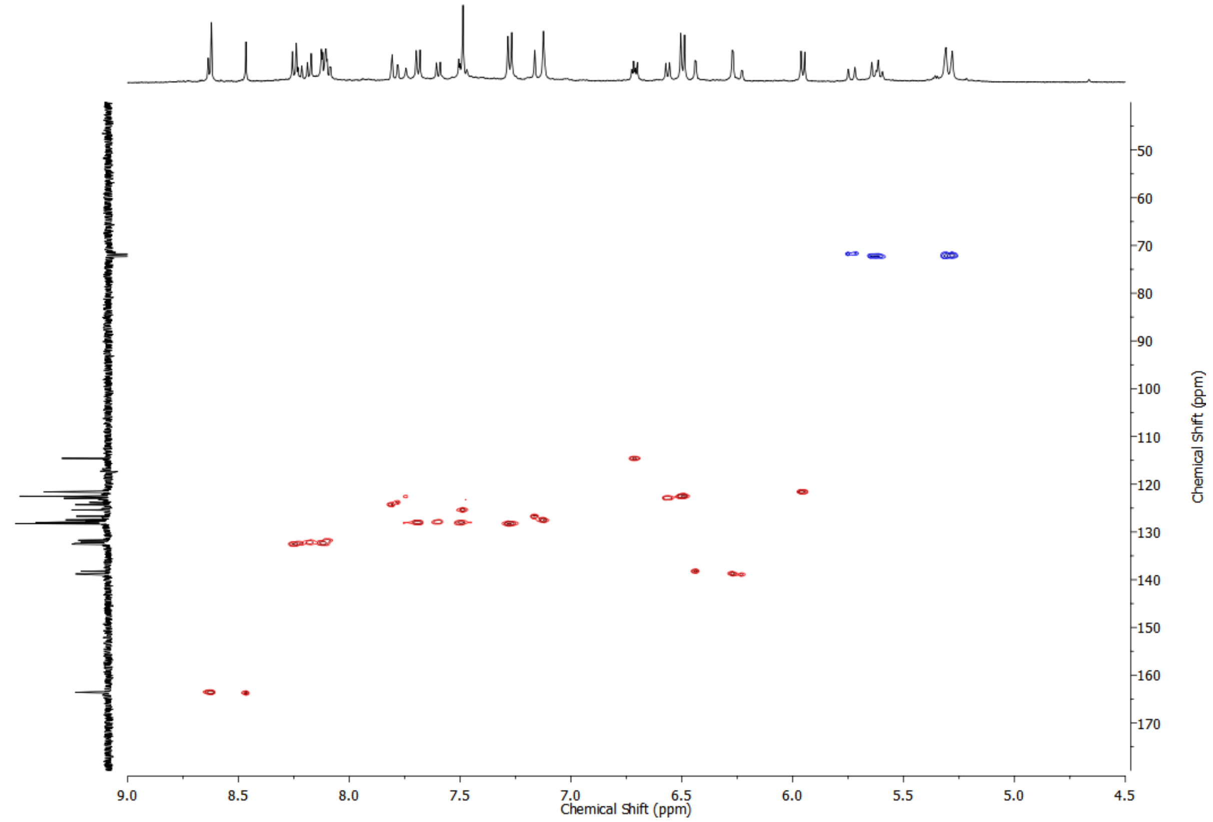

Figure S39. ^1H-^{13}C HSQC spectrum (500 MHz, CD_3CN, 298 K) of **2’**.

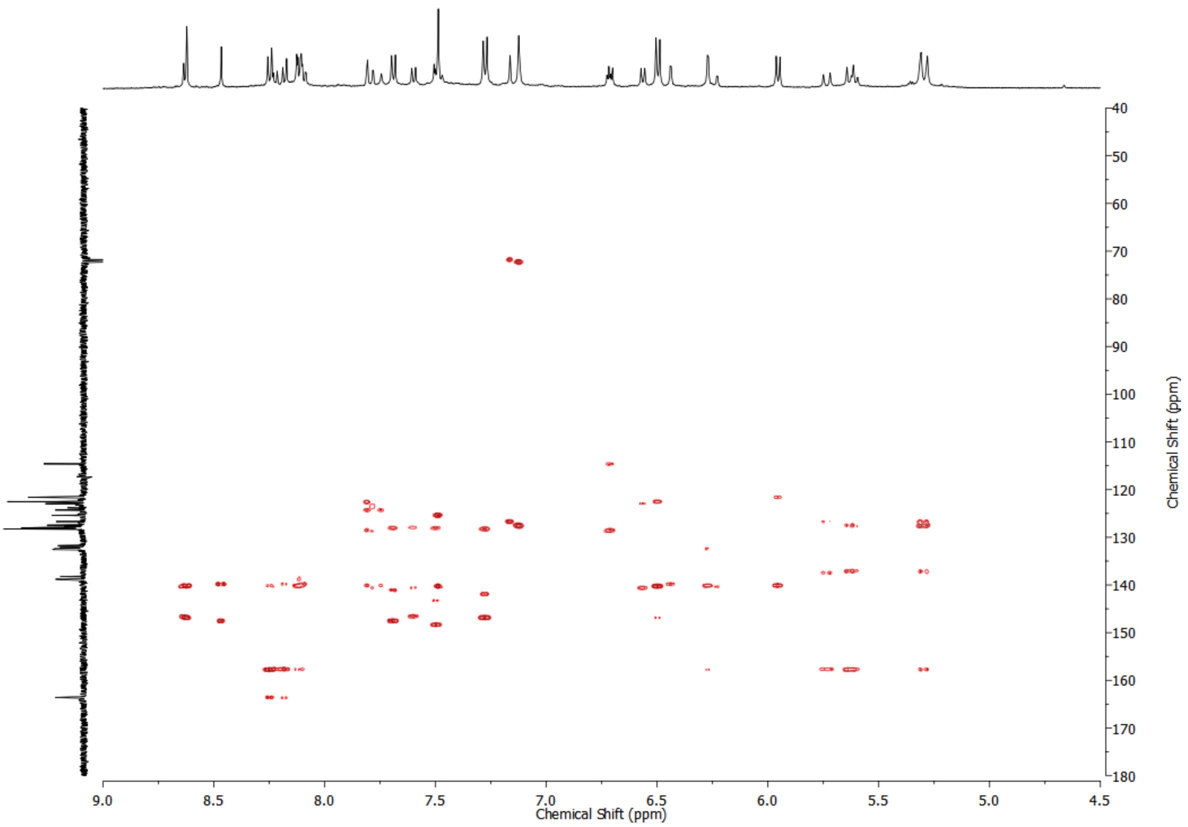

Figure S40. ^{1}H-^{13}C HMBC spectrum (500 MHz, CD_3CN, 298 K) of **2'**.

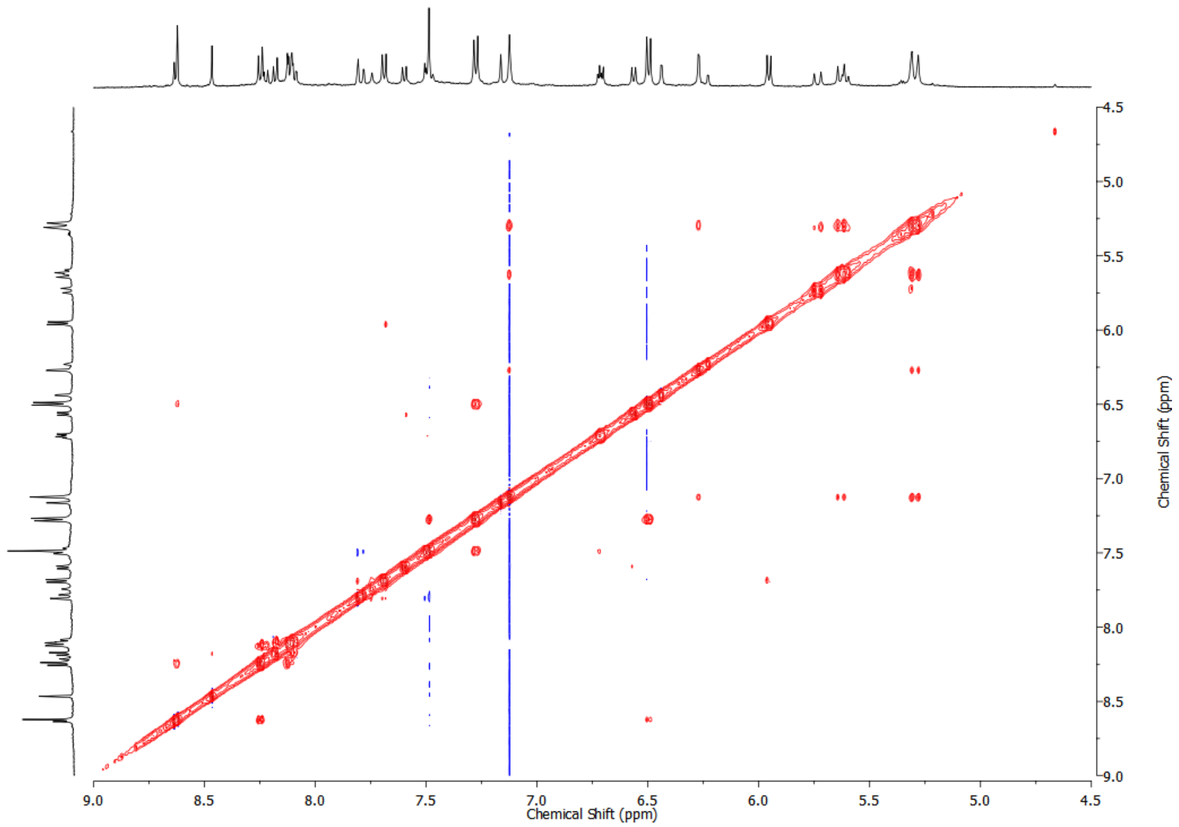

Figure S41. ^{1}H-^{1}H NOESY spectrum (500 MHz, CD_3CN, 298 K) of **2'**.

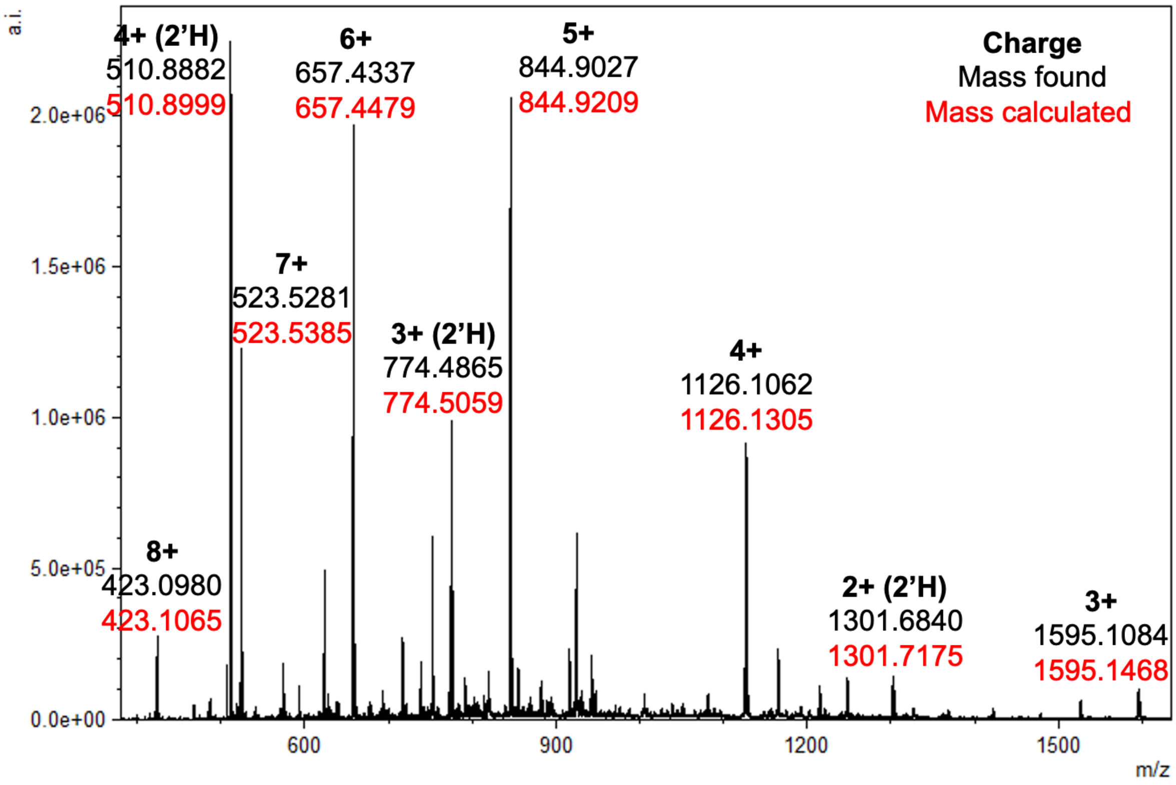

Figure S42. High resolution ESI-mass spectrum of **2'**.

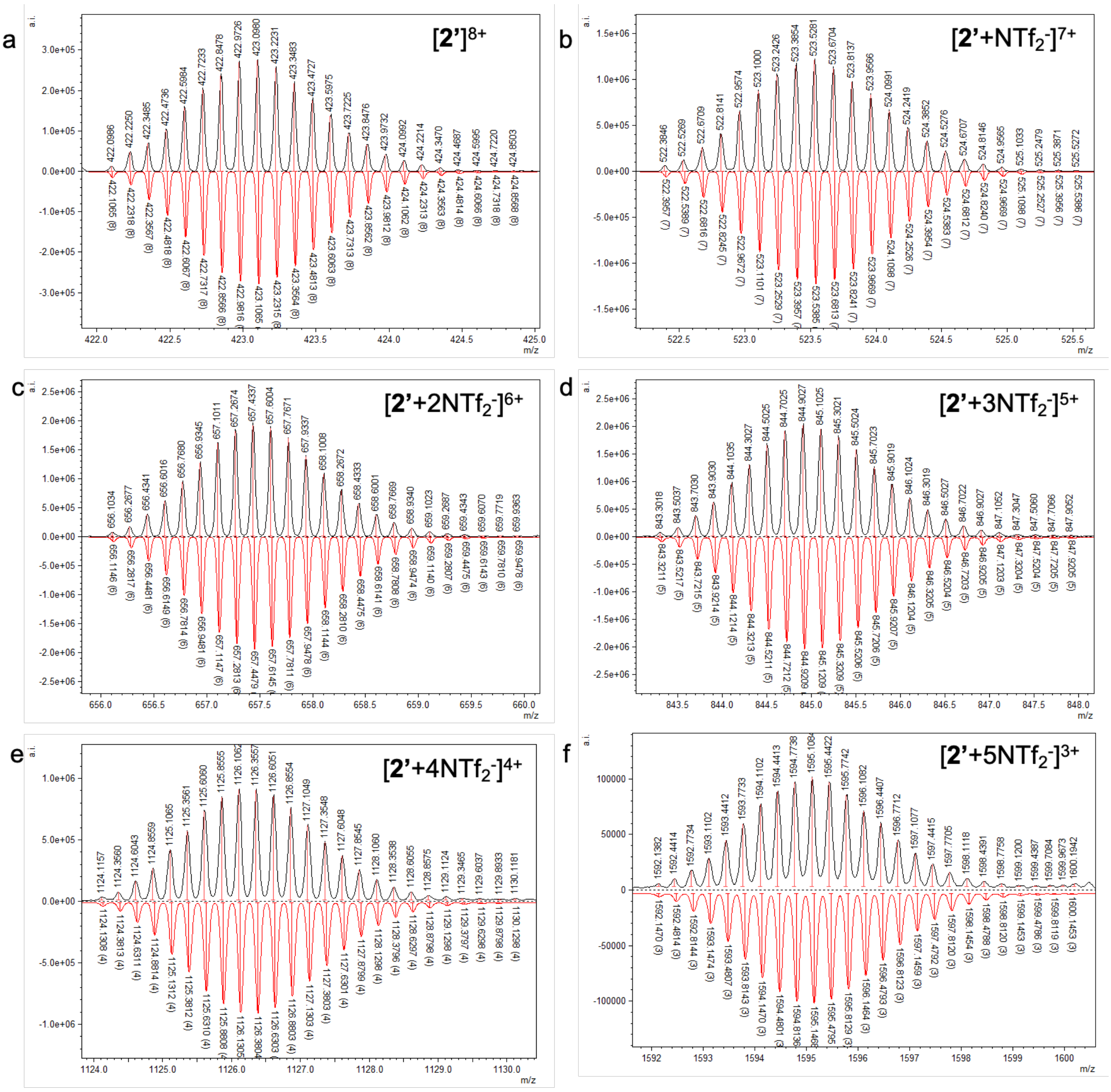

Figure S43. Signals from the HR-ESI-MS spectrum of **2’**. Experimental and calculated signals for (a) [**2’**]$^{8+}$; (b) [**2’**+NTf$_2^-$]$^{7+}$; (c) [**2’**+2NTf$_2^-$]$^{6+}$; (d) [**2’**+3NTf$_2^-$]$^{5+}$; (e) [**2’**+4NTf$_2^-$]$^{4+}$; (f) [**2’**+5NTf$_2^-$]$^{3+}$.

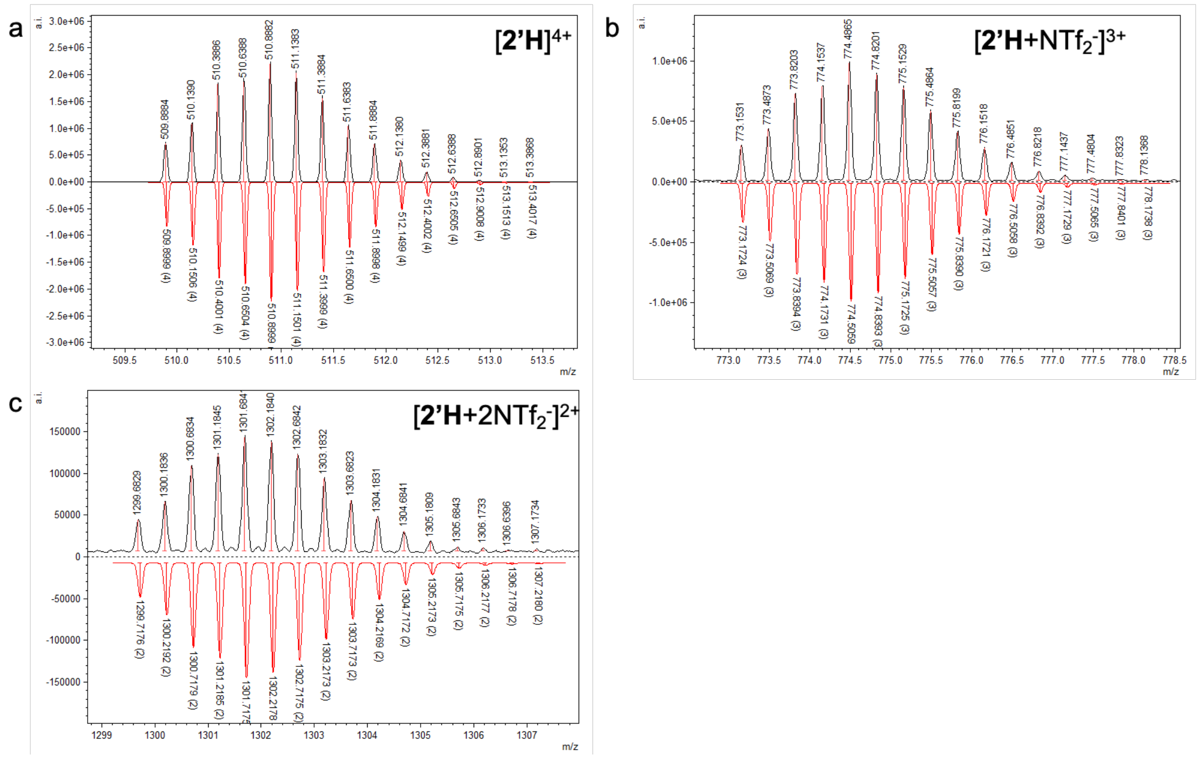

Figure S44. Signals from the HR-ESI-MS spectrum of helicate **2'H.** Experimental and calculated signals for (a) [**2'H**]$^{4+}$; (b) [**2'H**+NTf$_2^-$]$^{3+}$; (c) [**2'H**+2NTf$_2^-$]$^{2+}$.

S3.3 Synthesis and characterization of **3**

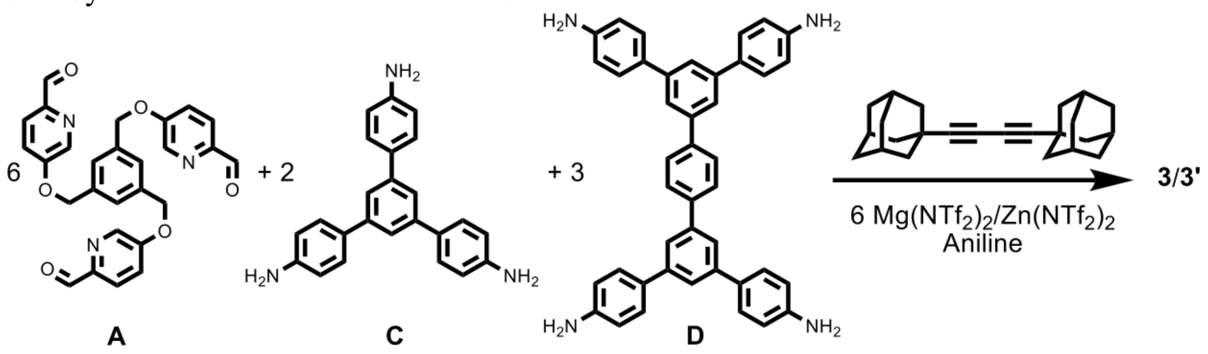

Scheme S6. Subcomponent self-assembly of **3**.

To a mixture of subcomponents tri-aniline **C** (1.05 mg, 3.00 µmol, 1.0 eq.), **A** (13.05 mg, 27.00 µmol, 9.0 eq.), tetra-aniline **D** (12.31 mg, 20.70 µmol, 6.9 eq.), Mg(NTf$_2$)$_2$ (15.76 mg, 26.96 µmol, 3.0 eq.),and guest **G5** (2.87 mg, 9.01 µmol, 3 eq.) in deuterated acetonitrile (3 mL), aniline (1.00 mg, 8.98 µmol, 3 eq.) was added. The purpose of adding extra aniline was to facilitate aniline exchange, thereby kinetically accelerating the formation of the desired structure. The resulting mixture was stirred at 70 °C and monitored daily. The imine signal corresponding to tetrahedron **2** gradually decreased over time, and by day 6, the system had reached equilibrium, with only signals from cage **3** observed in the ^{1}H NMR spectrum. The

reaction mixture was then allowed to cool to room temperature and the insoluble by-products were removed by filtration through a glass fiber plug. The filtrate was separated in 1 mL aliquots and each sample was diluted with diethyl ether to 15 mL. The resulting precipitate was dried under an N_2 flow. The product was obtained as a brown solid without further purification (8.10 mg, 63 %).

^{1}H NMR (500 MHz, CD_3CN, 298 K) δ 8.73 (s, 6H), 8.67 (s, 6H), 8.52 (s, 6H), 8.30 (d, *J* = 10 Hz, 6H), 8.26 (d, *J* = 10 Hz, 6H), 8.20 (d, *J* = 10 Hz, 6H), 8.12–8.08 (m, 18H), 7.91 (s, 6H), 7.73 (s, 12H), 7.63 (s, 6H), 7.61 (s, 3H), 7.59 (d, *J* = 10 Hz, 6H), 7.48 (s, 6H), 7.36 (d, *J* = 5 Hz, 12H), 7.29 (d, *J* = 10 Hz, 12H), 7.17 (s, 6H), 7.12 (s, 6H), 7.09 (s, 6H), 6.70–6.68 (m, 24H), 6.58 (d, *J* = 10 Hz, 12H), 6.46 (d, *J* = 5 Hz, 12H), 6.40 (d, *J* = 5 Hz, 12H), 6.21 (d, *J* = 5 Hz, 12H), 5.69–5.58 (m, 18H), 5.33–5.26 (m, 18H).

^{13}C NMR (125 MHz, CD_3CN, 298 K) δ 167.20, 166.45, 165.79, 157.41, 157.30, 157.27, 142.54, 142.27, 142.15, 141.97, 141.94, 141.84, 141.48, 140.27, 139.75, 139.53, 138.85, 137.14, 129.13, 128.20, 128.01, 127.13, 125.01, 122.45, 122.40, 122.21, 121.16, 119.69 (q, $^3J\_C\text{–}F \approx 320$ Hz, CF_3, NTf_2^-), 72.53, 71.89, 71.76, 41.07, 28.91, 26.58.

ESI-HR-MS ([**3**$(NTf_2)_{12}$] = $C_{336}H_{234}N_{36}O_{18}Mg_6(C_2F_6NO_4S_2)_{12}$) *m/z* = 498.9635 $[M+NTf_2]^{11+}$ (calc. 498.9710), 576.9499 $[M+2NTf_2]^{10+}$ (calc. 576.9601), 672.1576 $[M+3NTf_2]^{9+}$ (calc. 672.1687), 791.1649 $[M+4NTf_2]^{8+}$ (calc. 791.1792), 944.1775 $[M+5NTf_2]^{7+}$ (calc. 944.1933).

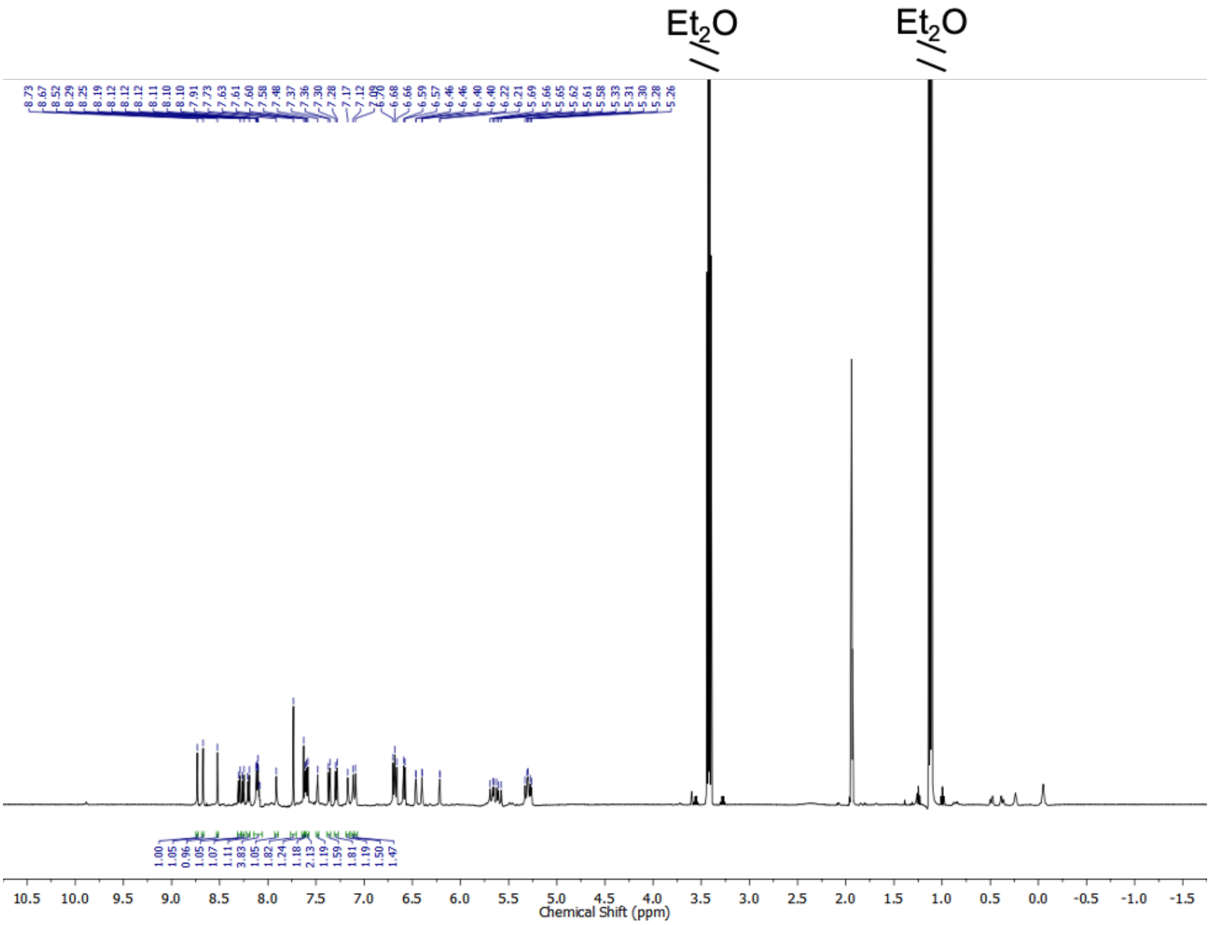

Figure S45. ^{1}H NMR spectrum (500 MHz, CD_3CN, 298 K) of **3**. The peak assignment is shown in Figure 2c in the main text.

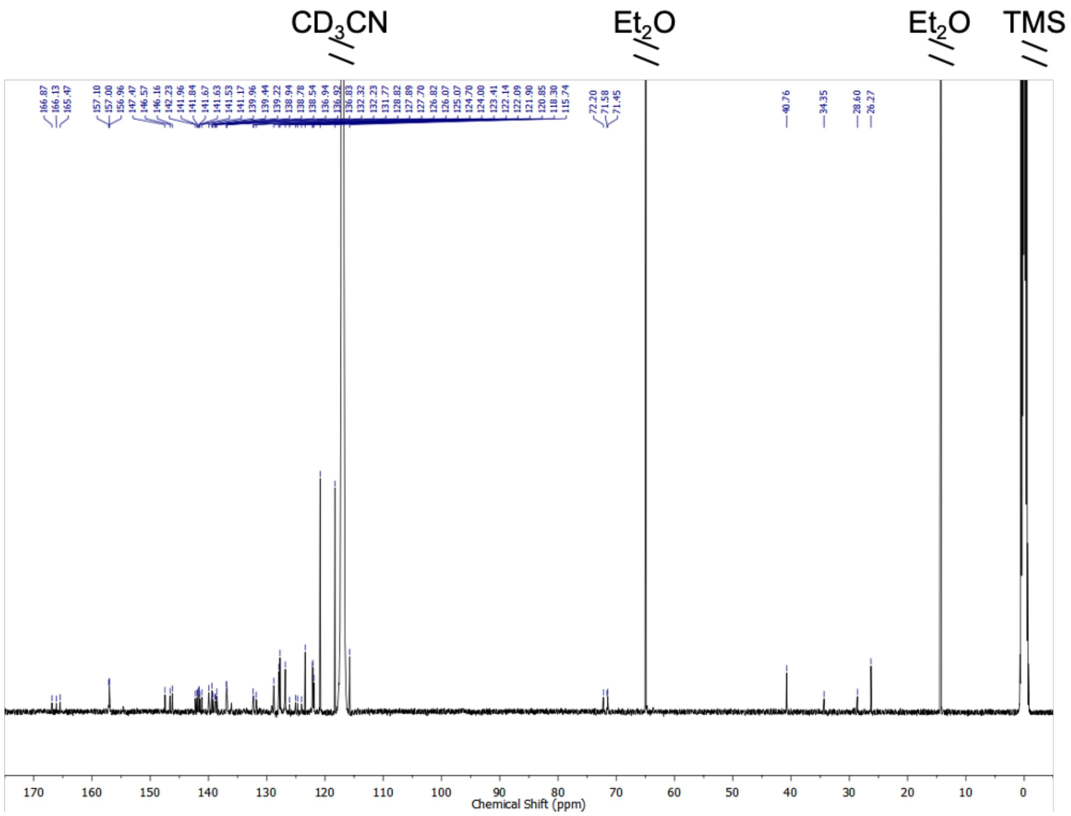

Figure S46. ^{13}C spectrum (126 MHz, CD_3CN, 298 K) of **3**.

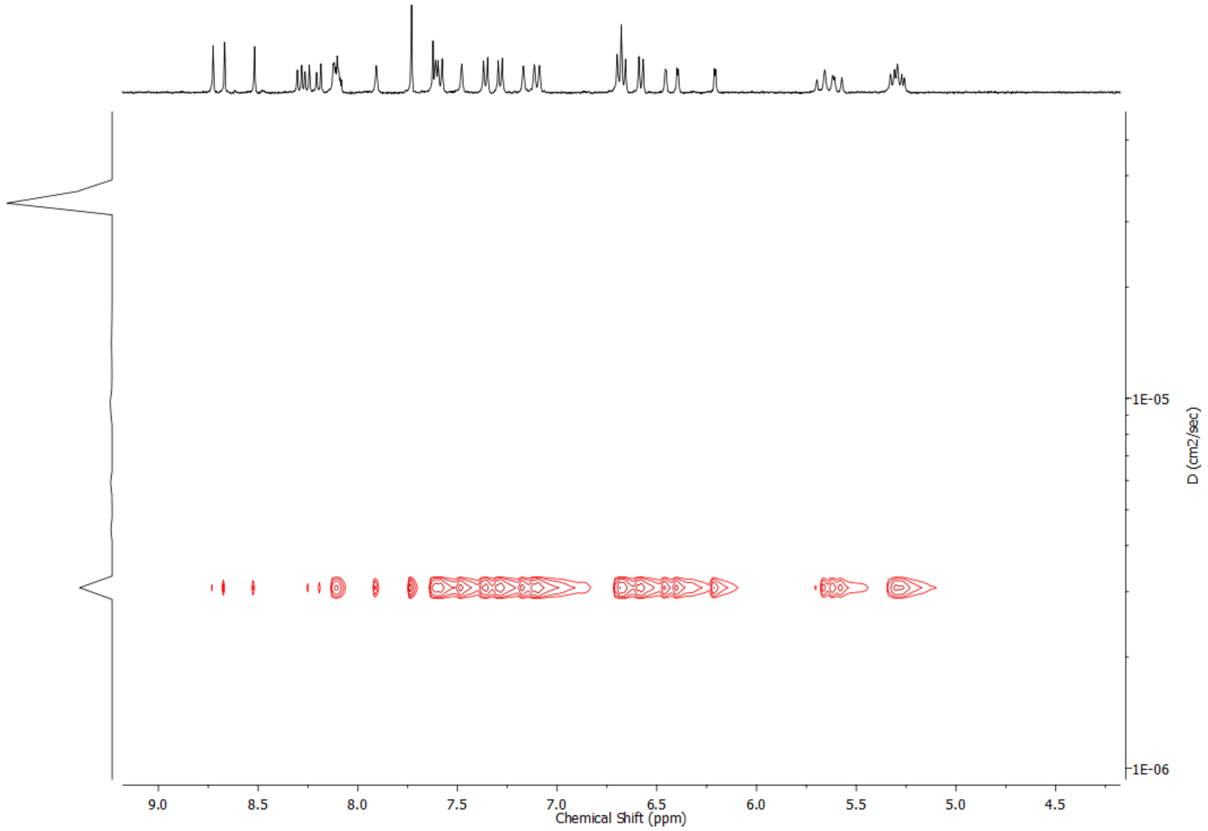

Figure S47. ^{1}H DOSY spectrum (400 MHz, CD_3CN, 298 K) of **3**.

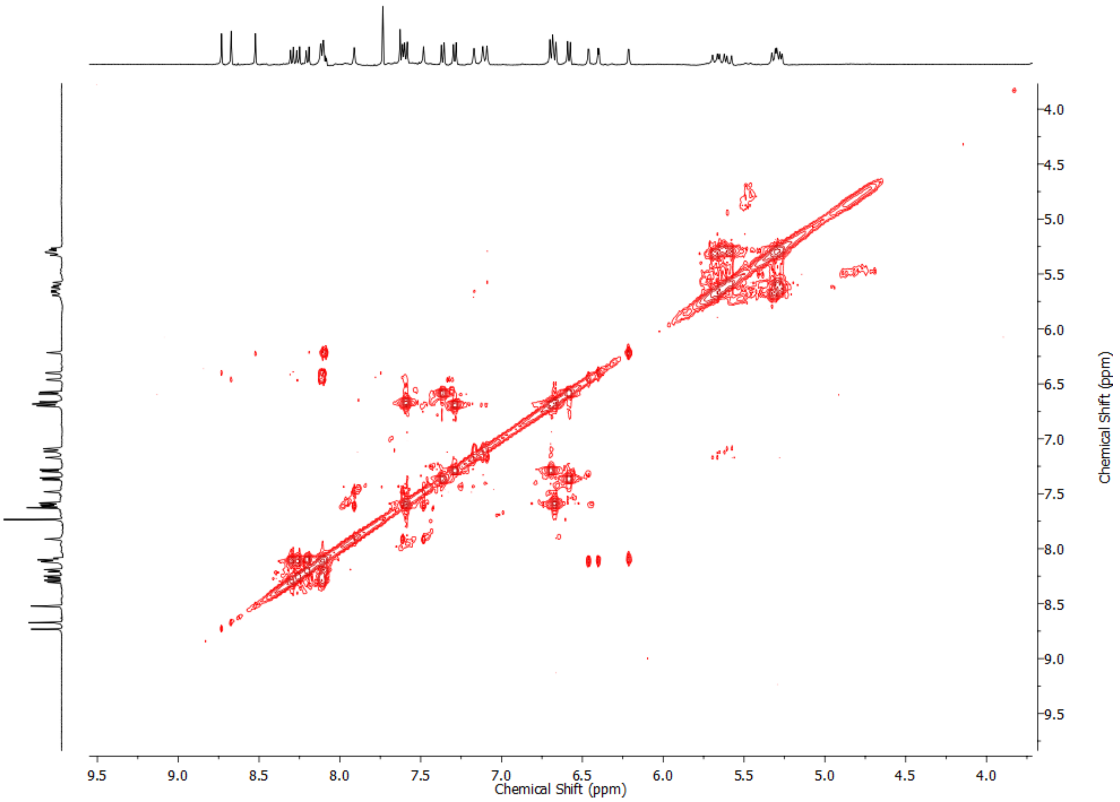

Figure S48. ^1H-^1H COSY spectrum (500 MHz, CD_3CN, 298 K) of **3**.

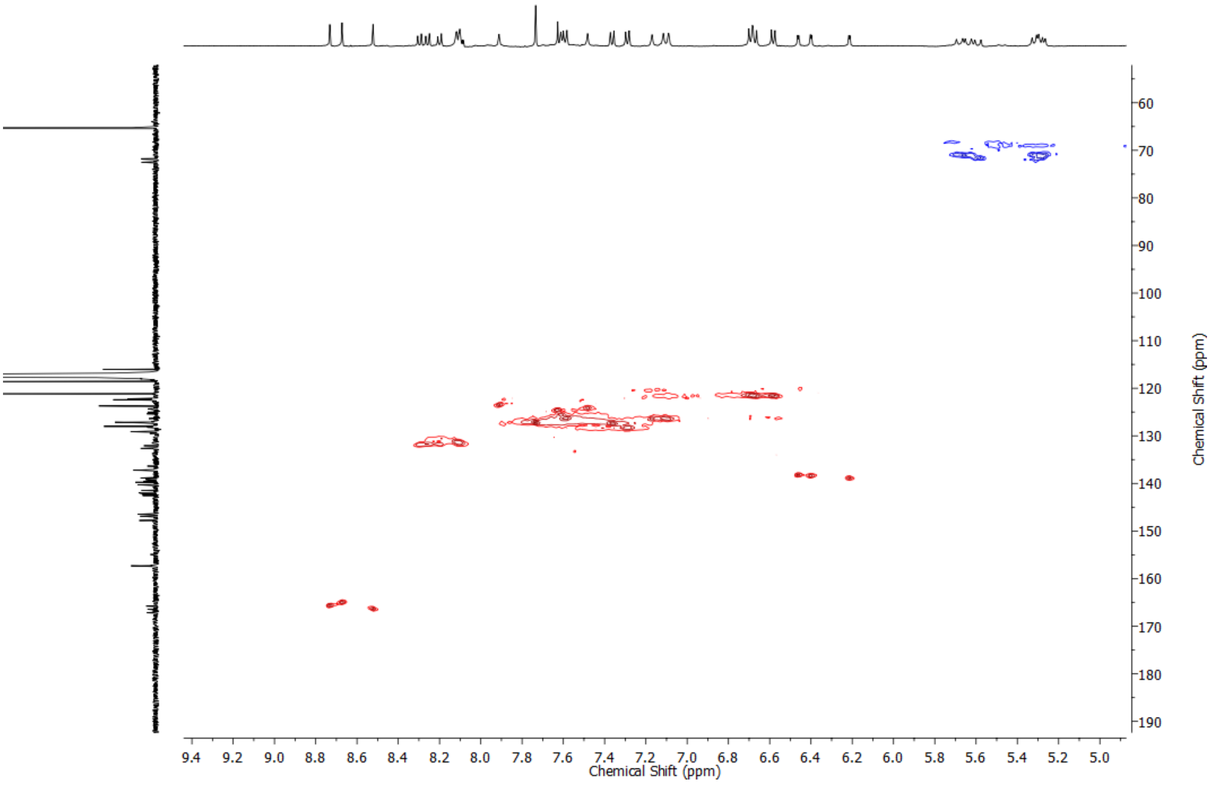

Figure S49. ^1H-^{13}C HSQC spectrum (500 MHz, CD_3CN, 298 K) of **3**.

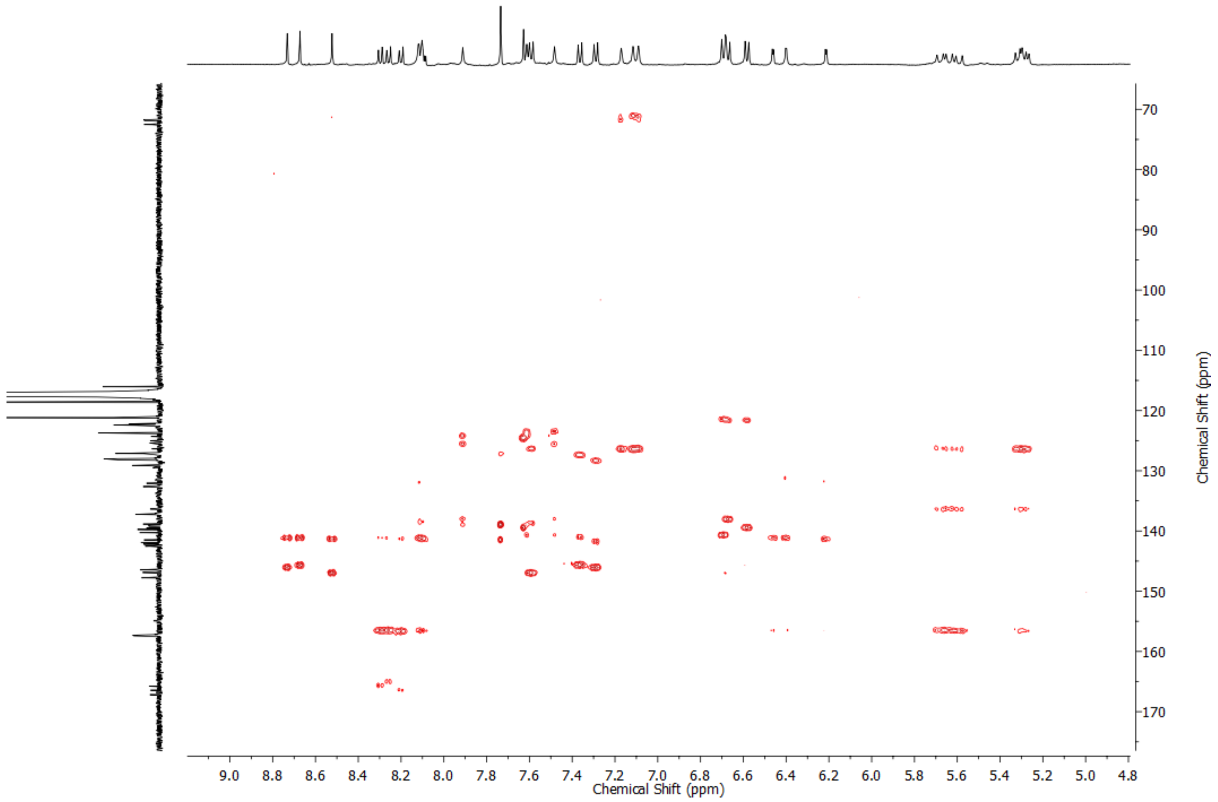

Figure S50. ^{1}H-^{13}C HMBC spectrum (500 MHz, CD_3CN, 298 K) of **3**.

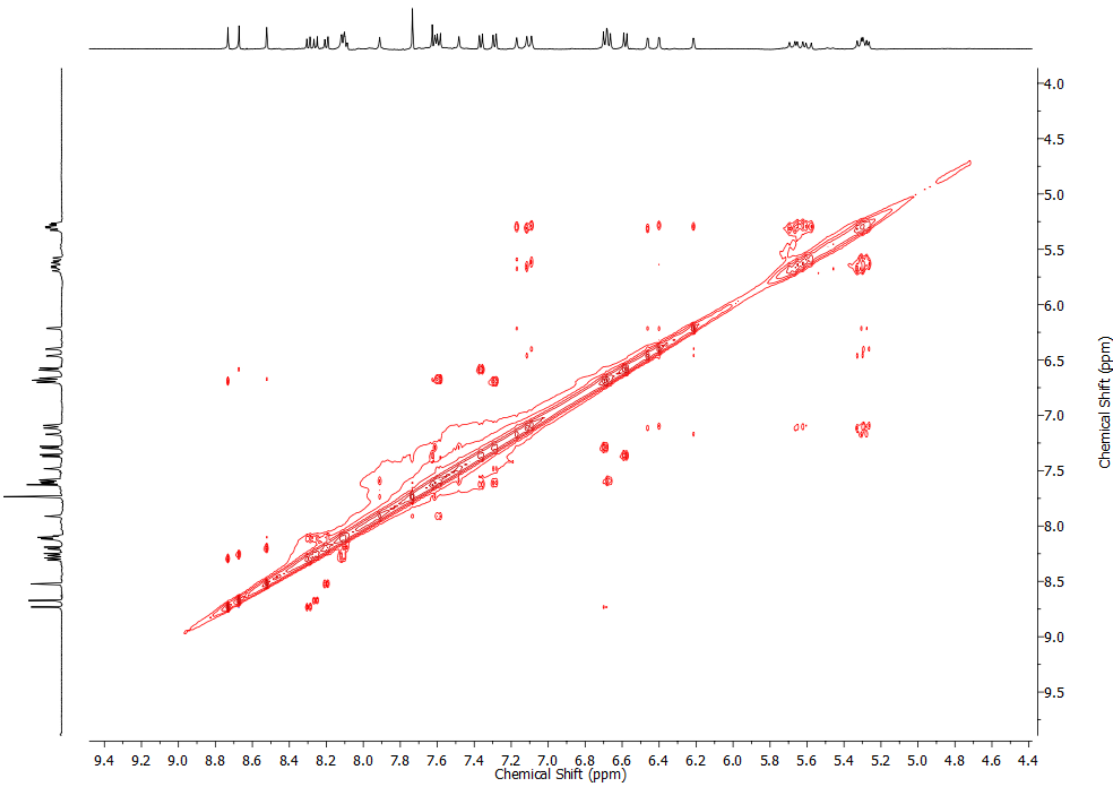

Figure S51. ^{1}H-^{1}H NOESY spectrum (500 MHz, CD_3CN, 298 K) of **3**.

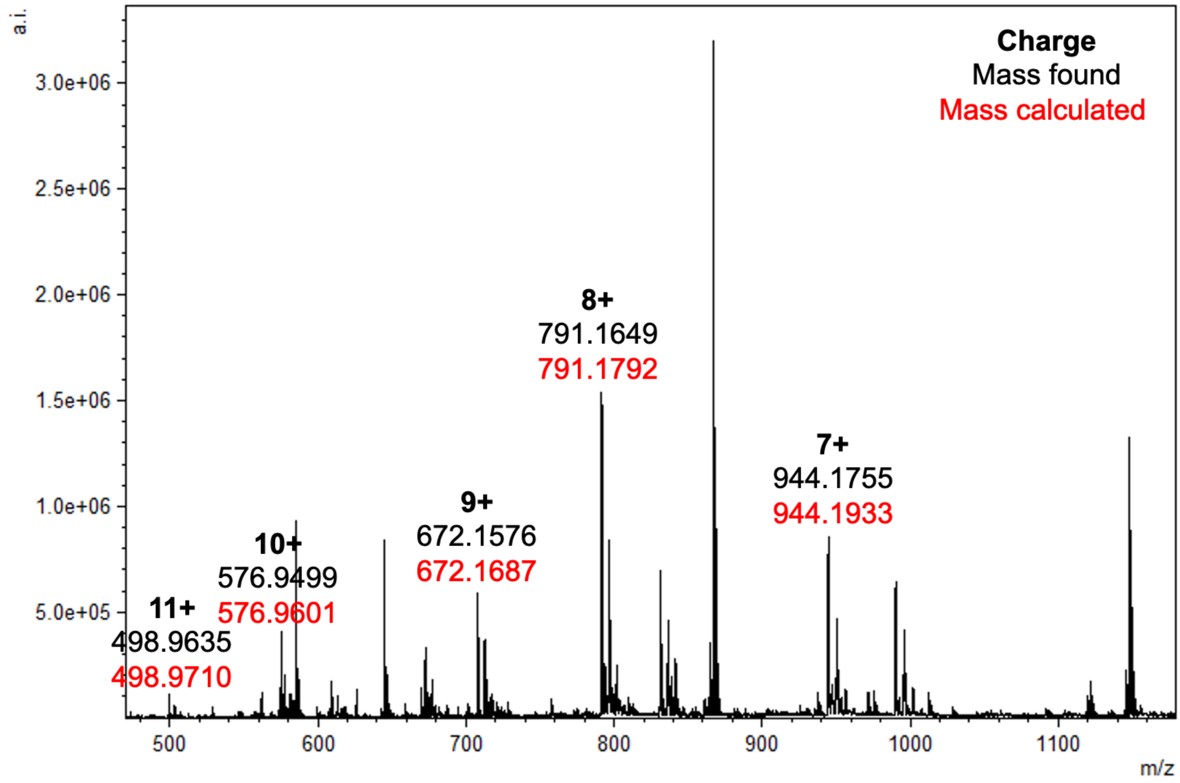

Figure S52. High resolution ESI-mass spectrum of **3**. Additional minor m/z peaks were observed; assignments to tetrahedral cage **2**, solvent-related adducts (CH_3CN or H_2O), and anion exchange were excluded. The remaining minor peaks are likely attributable to fragmentation of cage **3** during ionization.

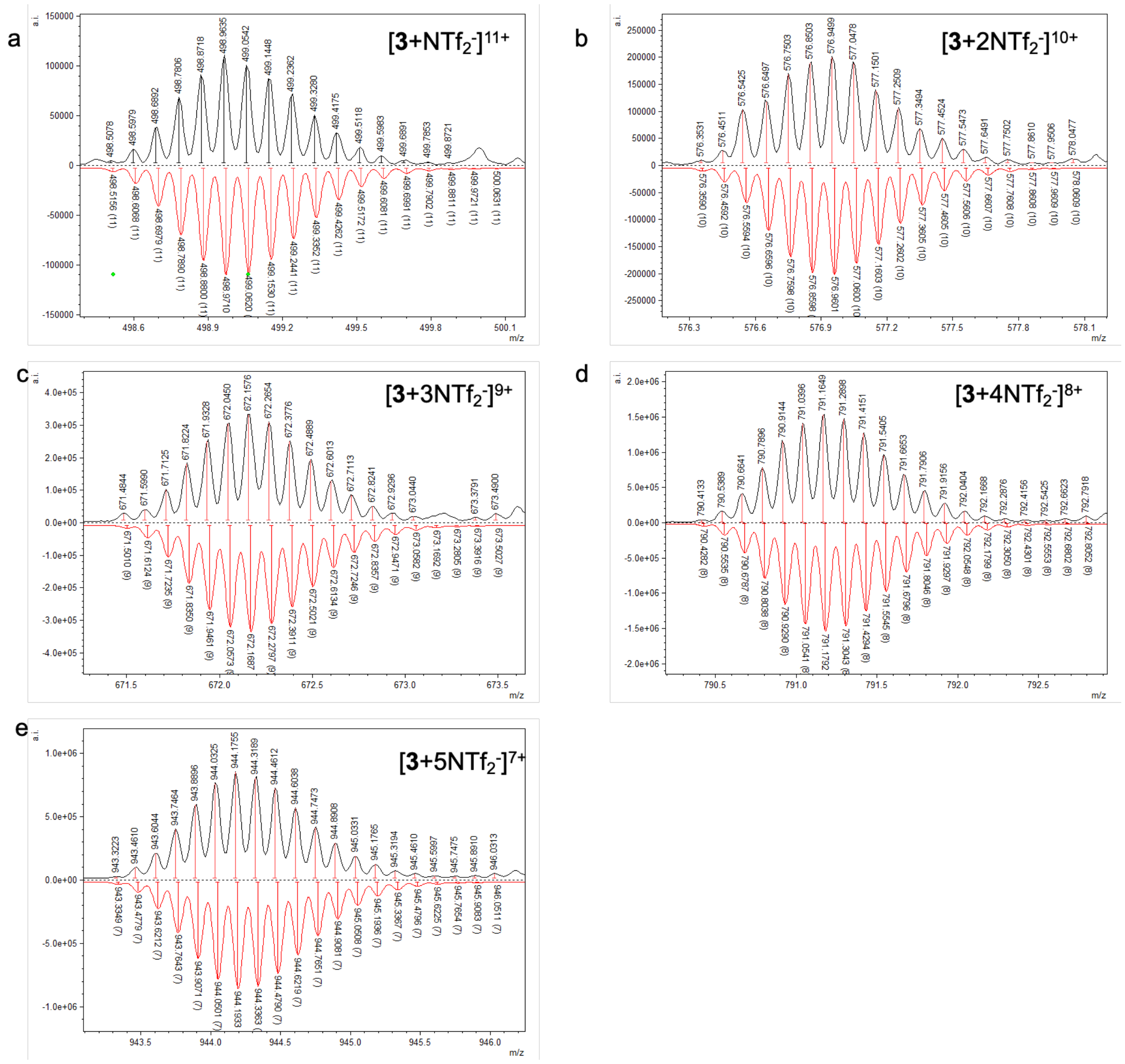

Figure S53. Signals from the HR-ESI-MS spectrum of **3**. Experimental and calculated signals for (a) [**3**+NTf$_2^-$]$^{11+}$; (b) [**3**+2NTf$_2^-$]$^{10+}$; (c) [**3**+3NTf$_2^-$]$^{9+}$; (d) [**3**+4NTf$_2^-$]$^{8+}$; (e) [**3**+5NTf$_2^-$]$^{7+}$.

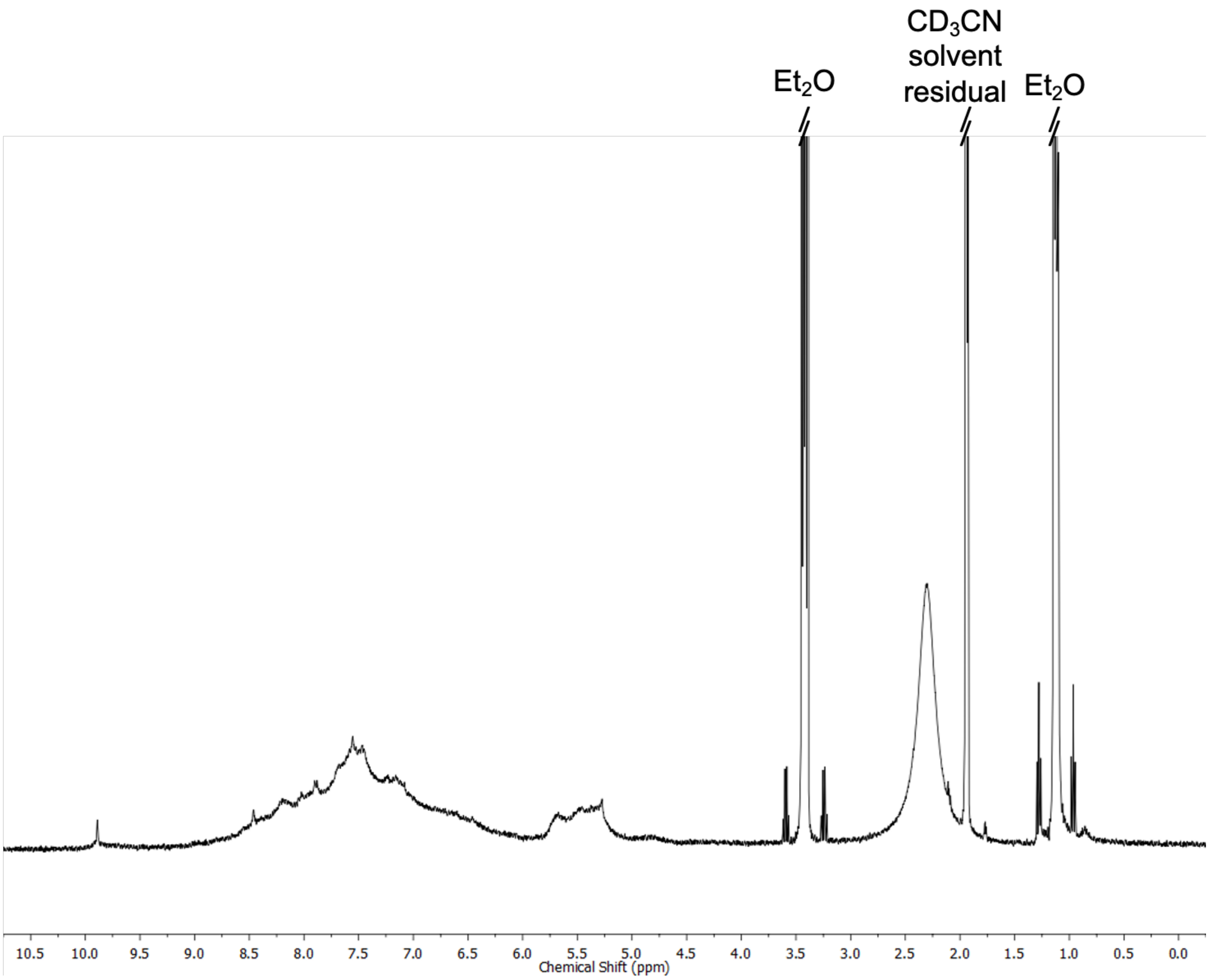

Figure S54. ^{1}H NMR spectrum of the reaction of subcomponents **A** (2.32 mg, 4.8 µmol) and **D** (1.79 mg, 3.0 µmol) with $Mg(NTf_2)_2$ (2.81 mg, 4.8 µmol) in CD_3CN (0.5 mL) at 70 °C overnight. No evidence of a discrete cage species was observed. HR-MS analysis showed no peaks corresponding to any cage species.

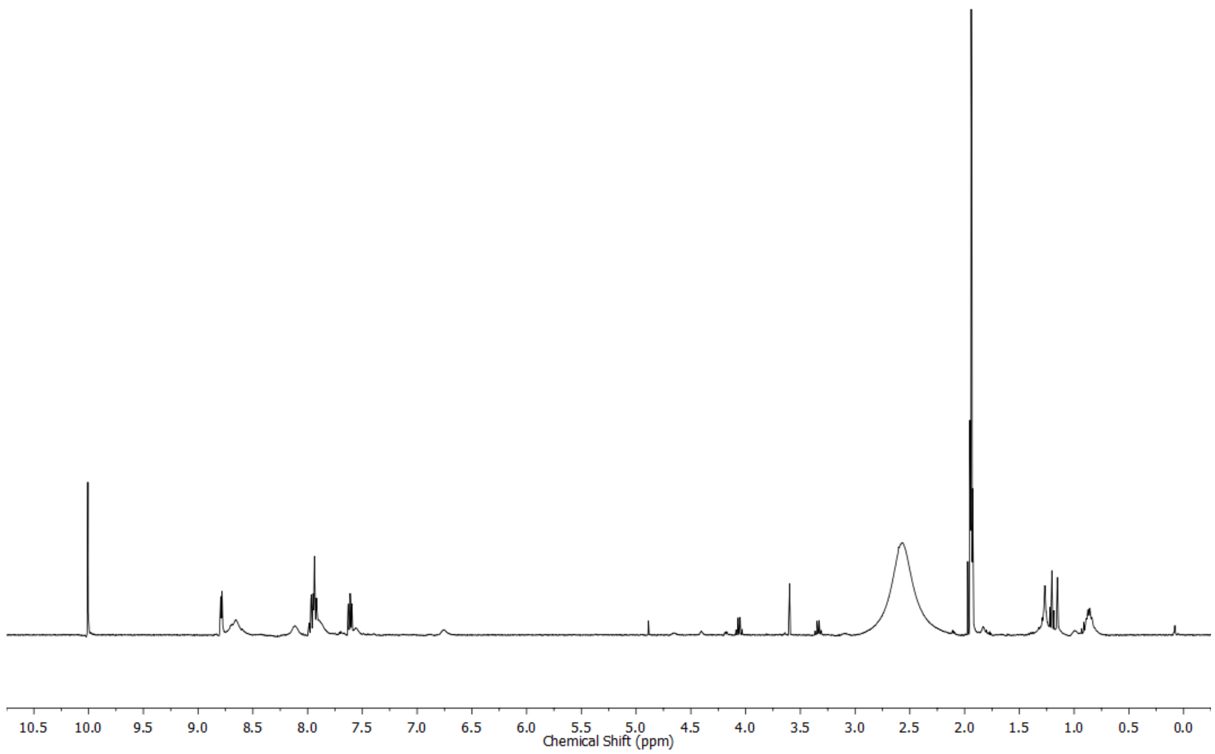

Figure S55. ^{1}H NMR spectrum (400 MHz, CD_3CN, 298 K) of a reaction mixture containing subcomponents **C**, **D**, and 2-formylpyridine with $Mg(NTf_2)_2$ in CD_3CN after heating at 70 °C overnight. No evidence for the formation of a discrete cage species was observed. HR-MS analysis also showed no peaks corresponding to any cage species.

S4 Host-guest studies

S4.1 General procedure

Solutions of cages **1**, **2**, and **3** (1 mM, 0.5 mL each) were prepared and transferred to NMR tubes. Guest molecules (6 equiv.) were then added to the corresponding cages, and the mixtures were stirred at 70 °C for 16 h.

The resulting solutions were analyzed by ^{1}H NMR spectroscopy and compared with the spectra of the corresponding empty cages. Guest binding was indicated by chemical shift changes (fast exchange regime) or the appearance of a new set of signals (slow exchange regime). For fast exchange, a chemical shift change of <0.01 ppm was considered negligible (no binding), whereas a change of >0.02 ppm was taken as evidence of guest binding.

S4.2 Host-guest study of cage **1**

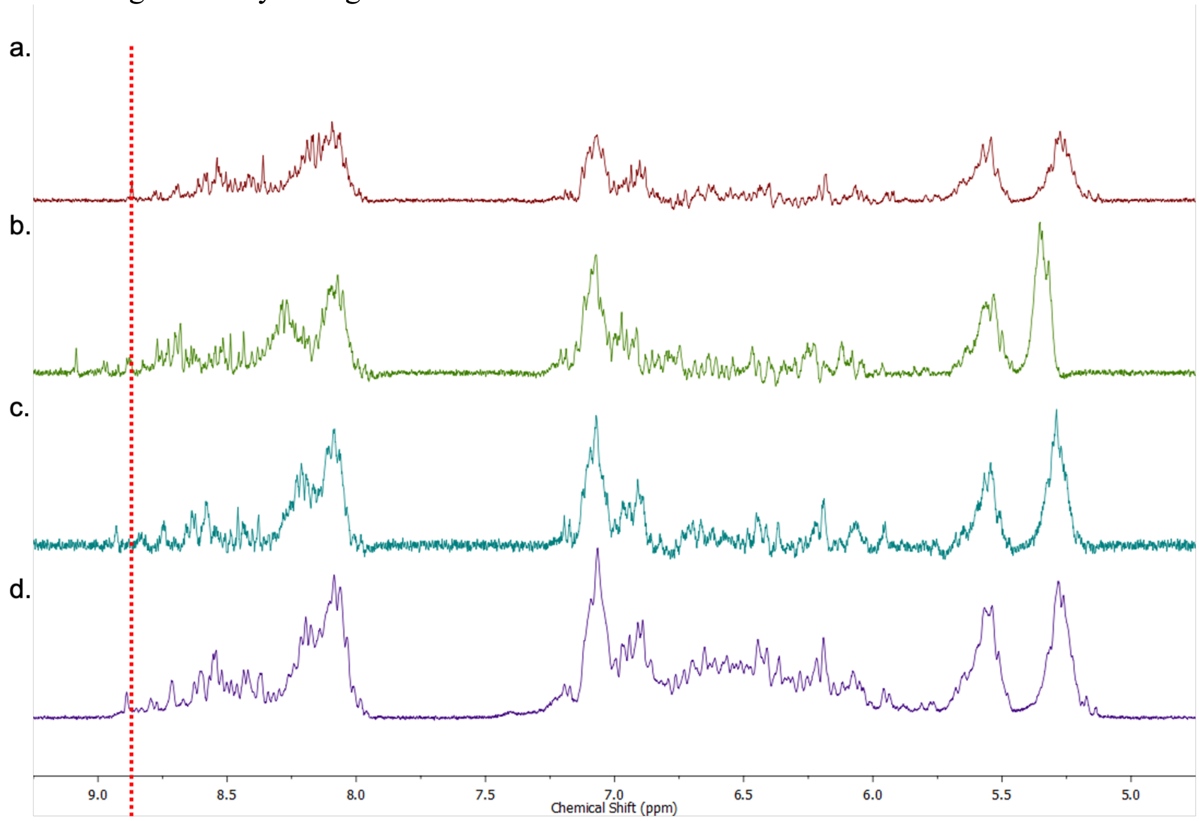

Figure S56. ^{1}H NMR spectra of (a) empty cage **1**; (b) cage **1** + TBAI; (c) cage **1** + TBABr: (d) cage **1** + $TBAClO_4$. Weak fast exchange binding was observed in each case.

S4.3 Host-guest study of cage **2**

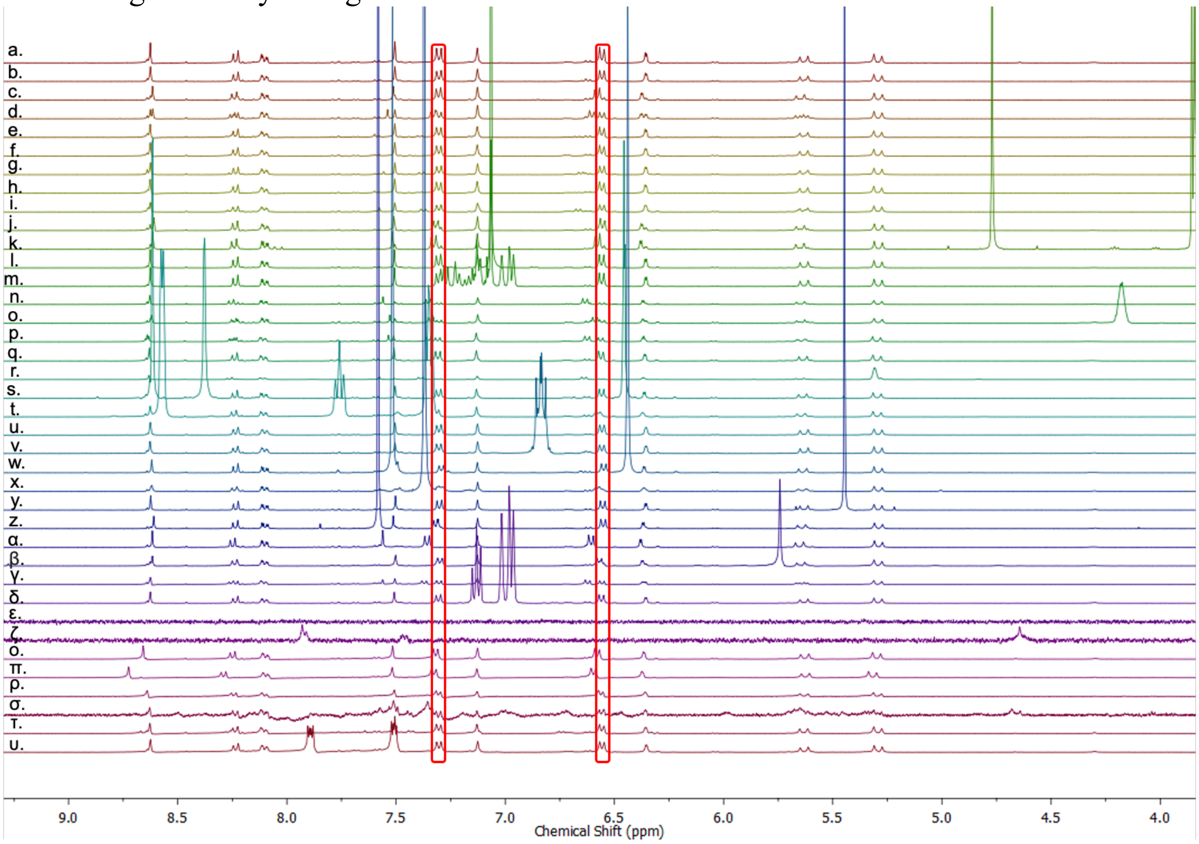

Figure S57. ^{1}H NMR spectra of (a) empty cage 2; (b–γ) upon addition of 6 equiv. of guest (b) *n*-pentane; (c) cyclopentane; (d) cyclohexane; (e) methylcyclohexane; (f) cyclooctane: (g) cyclohexanone; (h) heptane; (i) cycloheptane; (j) 1,4-dioxane; (k) 1,3-dioxane; (l) p-xylene; (m) o-xylene; (n) 1-methylcyclopentanol; (o) 1-methylcyclopentane; (p) cyclopentanol; (q)cyclopentanone; (r) hexane; (s) 1-methylcyclopentanol; (t) isoxazole; (u) pyridine; (v) n-octane; (w) 1,3,5-trifluorobenzene; (x) furan; (y) dichloromethane; (z) chloroform; (α) tetrachlorocarbon; (β) cyclopentene; (γ) cyclohexanol; (δ) *m*-xylene; (ε) $TBAPF_6$; (ζ) $TBABF_4$; (ο) $TBAClO_4$; (π) TBAI; (ρ) TBABr; (σ) TBACl; (τ) adamantane; (υ) naphthalene. The two highlighted peaks correspond to the protons on the peripheral phenyl rings of each tetrahedral face, which are the ones most strongly shifted upon guest encapsulation.

Table S1 Comparison of host-guest properties of cage **2** and its previously reported Fe^{2+} analogue.[4]

Guest	Volume (A3)	Encapsulation in Fe cage?	Encapsulation in Mg cage **2**?
cyclohexane	111.5	Yes	Yes
cyclohexene	107.8	Yes	Yes
cyclopentane	70.7	Yes	Yes
cyclopentene	90.7	Yes	Yes
cyclohexanone	114.3	Yes	Yes
cyclohexanol	119.1	Yes	Yes
1-methylcyclopentanol	120.5	Yes	Yes
Benzene	99.5	Yes	Yes
pyridine	92.8	Yes	No
isoxazole	70.7	Yes	No
n-pentane	106.5	No	No
carbon tetrachloride	88.7	Yes	Yes
chloroform	74.7	Yes	Yes
dichloromethane	60.9	Yes	No
PF_6^-	74.7	No	No
BF_4^-	53.7	No	No
ClO_4^-	54.8	Precipitation	Yes
I^-	34.8	No	Yes
Br^-	28.1	No	Precipitation
Cl^-	23.7	No	No
n-octane	161.9	No	No
1,3,5-trifluorobenzene	113.1	No	No
methylcyclohexane	129.7	No	No
o-xylene	135.1	No	No
m-xylene	135.6	No	No
cyclooctane	145.6	No	No
napthalene	151.0	No	No
adamantane	159.0	No	No

S4.4 Host-guest studies of cage **3**

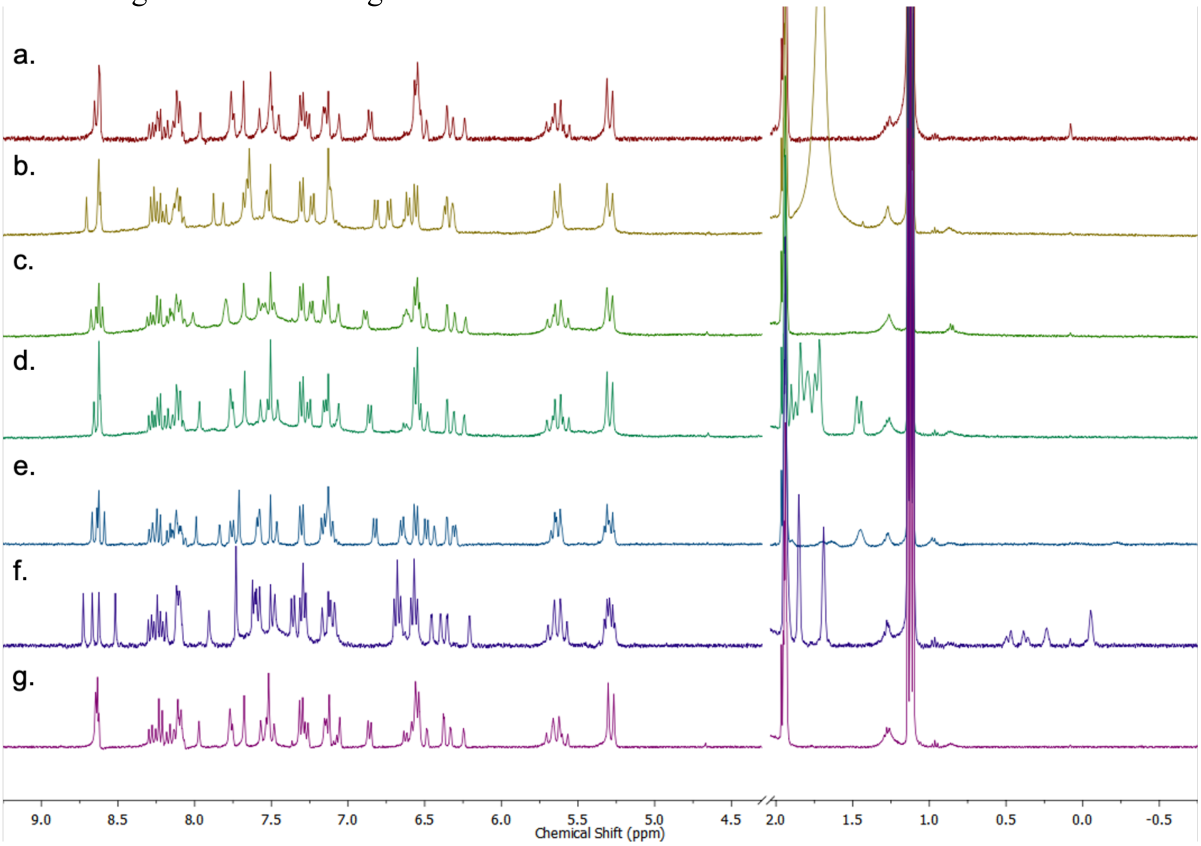

Figure S58. ^1H NMR spectra of (a) a mixture of cages **2** and **3** before guest addition; (b–g) after addition of (b) diamantane (**G1**) added; (c) 1,1'-diadamantyl (**G2**) added; (d) 2,2'-diadamantane (**G3**) added; (e) 1-[2-(1-adamantyl)ethyl]adamantane (**G4**) added; (f) 1-[4-(1-adamantyl)-1,3-butadiynyl]adamantane (**G5**) added; (g) sodium cobalticarborane (**G6**) added. All guest molecules were added at 6 equivalent relative to the cage. Fast exchange was observed in all cases.

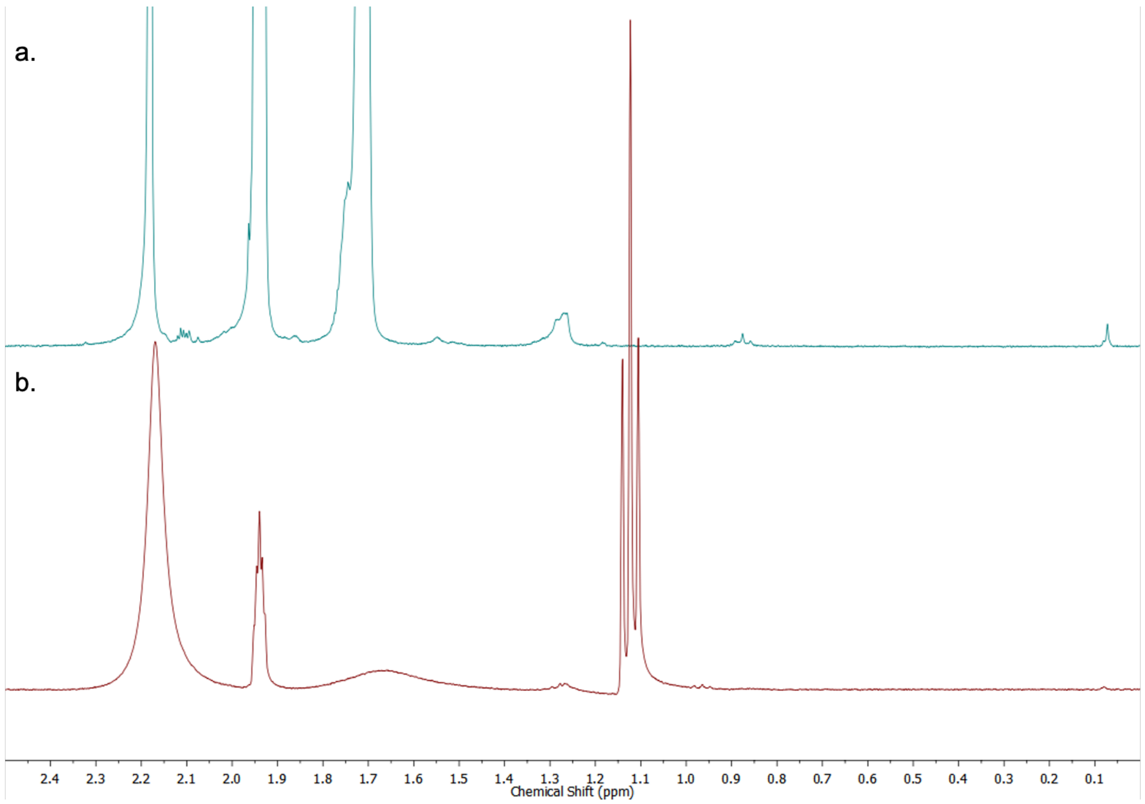

Figure S59. Partial ^1H NMR spectra of (a) diadamantane and (b) the cage mixture + diadamantane. Signals corresponding to the bound guest are not clearly detected, presumably due to overlap.

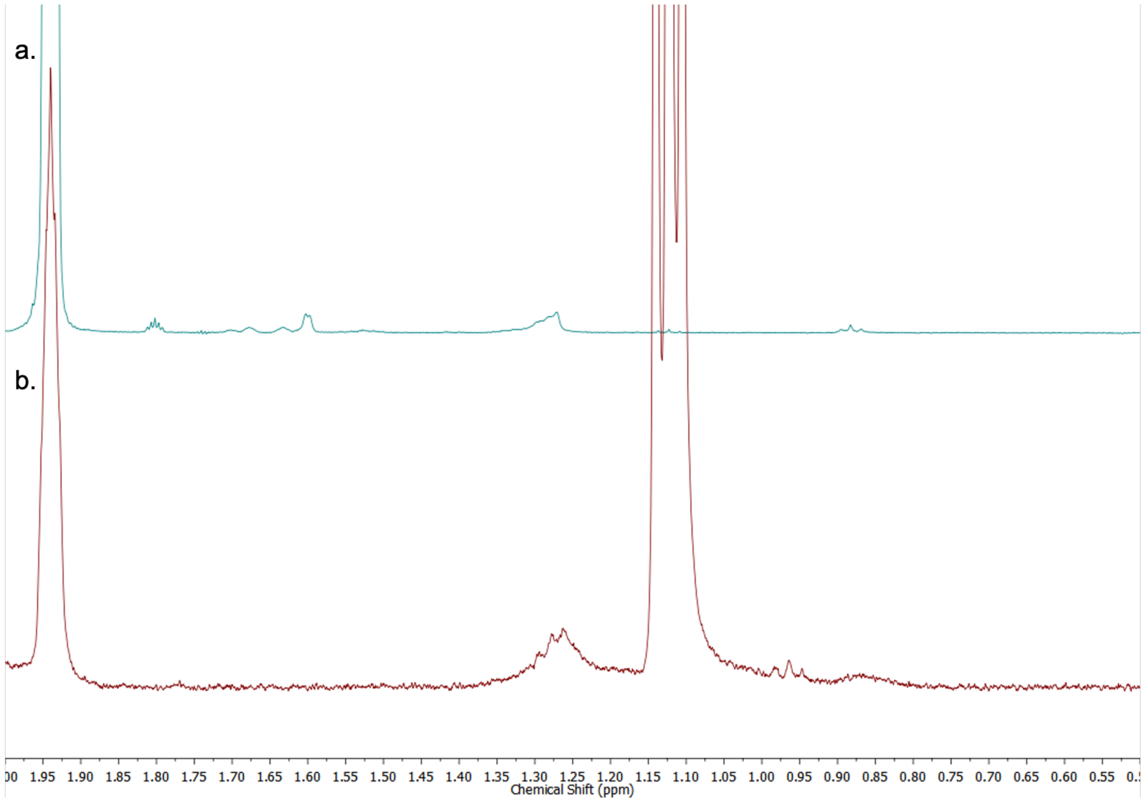

Figure S60. Partial ¹H NMR spectra of (a) 1,1'-biadamantyl and (b) the cage mixture + 1,1'-biadamantyl. Signals corresponding to the bound guest are not clearly detected, presumably due to low signal-to-noise ratio and overlap.

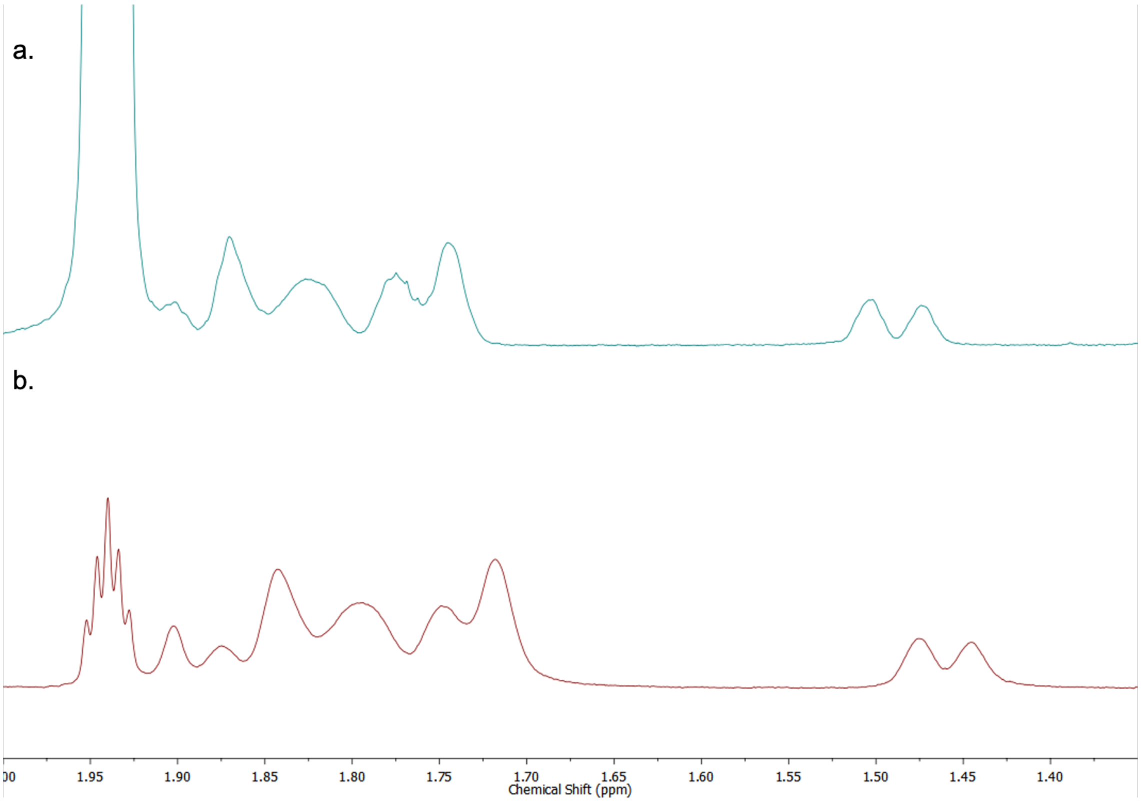

Figure S61. Partial ¹H NMR spectra of (a) 2,2'-biadamantyl and (b) the cage mixture + 2,2'-biadamantyl. Only minor chemical-shift changes (~ 0.03 ppm) were detected.

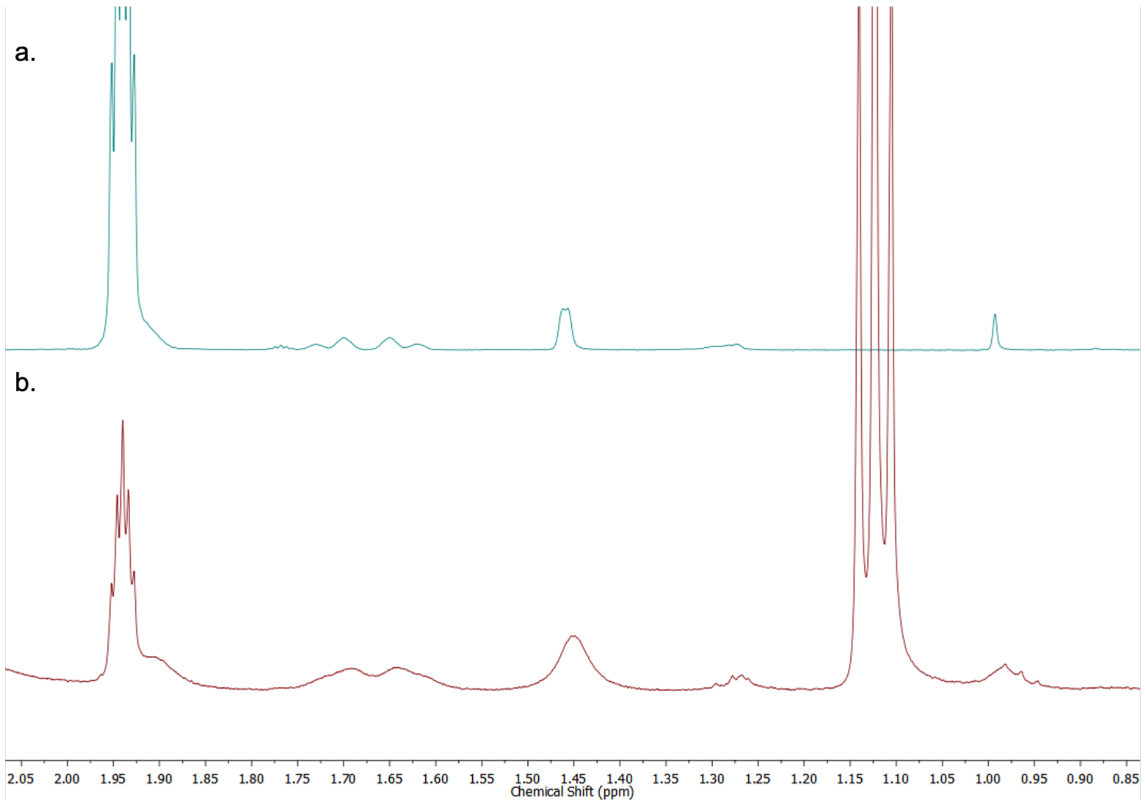

Figure S62. Partial ¹H NMR spectra of (a) 1-[2-(1-adamantyl)ethyl]adamantane and (b) the cage mixture + 1-[2-(1-adamantyl)ethyl]adamantane. Peak broadening was observed.

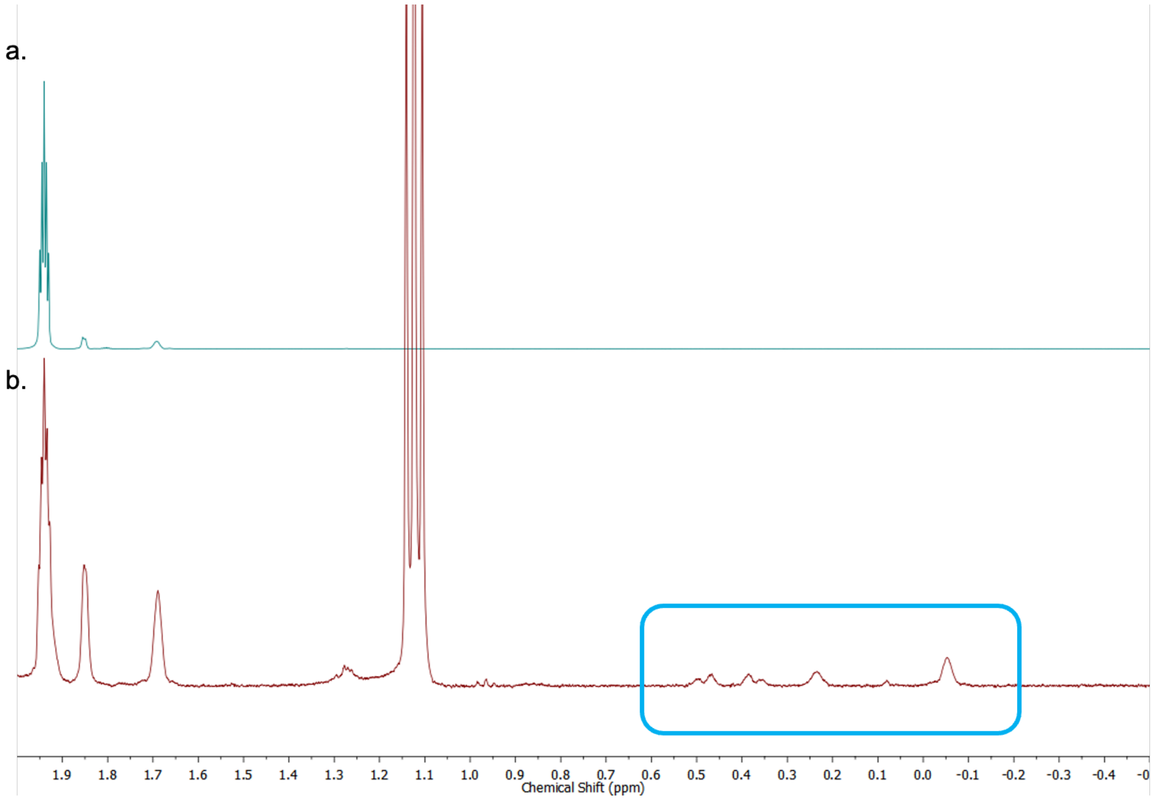

Figure S63. Partial ^1H NMR spectra of (a) 1-[4-(1-adamantyl)-1,3-butadiynyl]adamantane and (b) the cage mixture + 1-[4-(1-adamantyl)-1,3-butadiynyl]adamantane. Peaks assigned to the bound guest are circled.

S5 X-ray crystallography

Data were collected at Beamline I19 of Diamond Light Source employing silicon double crystal monochromated synchrotron radiation (0.6889 Å) with ω and ψ scans at 100(2) K.[5] Data integration and reduction were undertaken with Xia2.[6] Subsequent computations were carried out using the WinGX-32 graphical user interface.[7] A multi-scan empirical absorption correction using spherical harmonics was applied to the data using DIALS.[6b] The structure was solved by intrinsic phasing using SHELXT[8] then refined and extended with SHELXL.[9] Carbon-bound hydrogen atoms were included in idealised positions and refined using a riding model. Disorder was modelled using standard crystallographic methods including constraints and restraints where necessary.

Crystallographic data have been deposited with the CCDC (2499153-2499154).

1·8NTf₂·4MeCN

$C_{216}H_{168}F_{48}Mg_4N_{36}O_{44}S_{16}$, *M* 5494.05, Tetragonal, space group I 41/a (#88), *a* 26.76090(10), *b* 26.76090(10), *c* 35.1086(2) Å, *V* 25142.9(2) Å^3, D_c 1.451 g cm^{-3}, *Z* 4, crystal size 0.200 by 0.200 by 0.050 mm, colour colourless, habit block, temperature 100(2) Kelvin, λ(Synchrotron) 0.6889 Å, μ(Synchrotron) 0.236 mm^{-1}, T(Analytical)$_{min,max}$ 0.811438873397347, 1.0, $2\theta_{max}$ 51.01, *hkl* range -33 32, -32 33, -42 41, *N* 94707, N_{ind} 12751(R_{merge} 0.0340), N_{obs} 6688(I > 2σ(I)), N_{var} 1101, residuals* $R1(F)$ 0.1471, $wR2(F^2)$ 0.4400, GoF(all) 0.993, $\Delta\rho_{min,max}$ -0.479, 0.602 e$^-$ Å^{-3}.

*$R1 = \Sigma||F_o| - |F_c||/\Sigma|F_o|$ for $F_o > 2\sigma(F_o)$; $wR2 = (\Sigma w(F_o^2 - F_c^2)^2/\Sigma(wF_c^2)^2)^{1/2}$ all reflections $w=1/[\sigma^2(F_o^2)+(0.3514P)^2]$ where $P=(F_o^2+2F_c^2)/3$

Specific refinement details:

The crystals of **1**·8NTf₂·4MeCN were grown by diffusion of diisopropyl ether into an acetonitrile solution of the complex. The crystals employed immediately lost solvent after removal from the mother liquor. However rapid handling prior to flash cooling in liquid nitrogen and the use of synchrotron radiation enabled the collection of data to around 0.8 Å. The diffraction pattern was broad and diffuse and the quality of the integration was lower than ideal. As a consequence the values of the R1, wR and wR2 factors are larger than typical small molecule structures.

The asymmetric unit was found to contain ¼ of a cage **1** assembly as well as associated counterions and solvent molecules. Thermal parameter restraints (SIMU, RIGU) were applied to all atoms except for magnesium.
Reflecting the poor diffraction properties of the crystals there is a very high level of disorder within the structure. One coordinating pyridyl-imine group was modelled as disordered over two locations. The anions and solvent molecules within the structure were all highly disordered with the triflimide anions modelled as disordered over a number of overlapping lattice sites. The occupancies of the disordered triflimide anions were allowed to refine freely. Some additional minor occupancy positions of the anions could not be located in the electron density map and were not included in the model resulting in a discrepancy of 0.8 counterions per asymmetric unit (or 3.1 per cage **1** assembly). Some lower occupancy disordered atoms were modelled with isotropic thermal parameters bond length and thermal parameter restraints were applied to facilitate realistic modelling of the disordered triflimide anions. Bond length restraints were also applied to the disordered acetonitrile molecules and their hydrogen atoms were not included in the formula.
CheckCIF gives two A and one B level alert, all resulting from the formula discrepancy discussed above and the poor diffraction properties of the crystals.

2·8NTf$_2$·1.5Et$_2$O·3MeCN

Formula $C_{232}H_{168}F_{48}Mg_4N_{35}O_{45.50}S_{16}$, *M* 5696.20, Trigonal, space group R3c (#161), *a* 32.25080(10), *b* 32.25080(10), *c* 44.0446(3) Å, γ 120°, *V* 39673.8(4) Å^3, D_c 1.430 g cm^{-3}, *Z* 6, crystal size 0.150 by 0.100 by 0.050 mm, colour colourless, habit block, temperature 100(2) Kelvin, λ(Synchrotron) 0.6889 Å, μ(Synchrotron) 0.228 mm^{-1}, *T*(Analytical)$_{min,max}$ 0.8916503938106335, 1.0, $2\theta_{max}$ 42.52, *hkl* range -33 31, -33 33, -42 45, *N* 49762, N_{ind} 10642(R_{merge} 0.0257), N_{obs} 5803(I > 2σ(I)), N_{var} 1193, residuals* $R1(F)$ 0.1363, $wR2(F^2)$ 0.3391, GoF(all) 1.004, $\Delta\rho_{min,max}$ -0.428, 0.751 e$^-$ Å^{-3}.

*$R1 = \Sigma||F_o| - |F_c||/\Sigma|F_o|$ for $F_o > 2\sigma(F_o)$; $wR2 = (\Sigma w(F_o^2 - F_c^2)^2/\Sigma(wF_c^2)^2)^{1/2}$ all reflections $w=1/[\sigma^2(F_o^2)+(0.2471P)^2]$ where $P=(F_o^2+2F_c^2)/3$

Specific refinement details:

The crystals of **2**·$8NTf_2$·$1.5Et_2O$·3MeCN were grown by diffusion of diethyl ether into an acetontrile solution of the complex. The crystals employed immediately lost solvent after removal from the mother liquor and rapid handling prior to flash cooling in liquid nitrogen was required to collect data. Despite these measures and the use of synchrotron radiation few reflections at greater than 0.95 Å resolution were observed and the data were trimmed accordingly. Nevertheless, the quality of the data is far more than sufficient to establish the connectivity of the structure.

The asymmetric unit was found to contain 1/3 of a cage **2** assembly as well as associated counterions and solvent molecules. Thermal parameter restraints (SIMU, RIGU) were applied to all atoms except for magnesium.

Reflecting the poor diffraction properties of the crystals there is disorder and a significant amount of thermal motion within the structure. The capping ligand coordinated to Mg(2) shows evidence of substantial thermal motion. Restraints, using the GRADE Web Server,[10] were applied to its bond lengths and angles in order to obtain a reasonable model. Even with these restraints the thermal parameters in this part of the structure are higher than ideal. Attempts to model this disorder over discrete positions did not improve the model.

The anions within the structure also show evidence of disorder. One triflimide anion was modelled as disordered over two locations and the remaining triflimides show evidence of unresolved disorder. The occupancies of the disordered triflimide anions were allowed to refine freely. Some additional minor occupancy positions of the anions (probably overlapping with the located anions) could not modelled and were not included in the model resulting in a discrepancy of 1.25 counterions per asymmetric unit (or ca. 3.8 per cage **2** assembly). Some lower occupancy disordered atoms were modelled with isotropic thermal parameters bond length and thermal parameter restraints were applied to facilitate realistic modelling of the disordered triflimide anions. The located solvent molecules were modelled with partial occupancy.

The SQUEEZE[11] function of PLATON[12] was employed to remove the contribution of the electron density associated with further highly disordered solvent, which gave a potential solvent accessible void of 1559 $Å^3$ per unit cell (a total of approximately 400 electrons). Diffuse solvent molecules could not be assigned to acetonitrile or diethyl ether and were therefore not included in the formula. Consequently, the molecular weight and density given above are slightly underestimated.

CheckCIF gives one A and four B level alerts, all resulting from the limited resolution of the data and the thermal motion around one of the vertices.

S6 Volume calculations

The volume of the cavity of cages **1**–**3** was calculated from either corresponding crystal structure or modelled structure using MoloVol.[13] The volume calculation for cage **2** failed because the cavity was divided into multiple smaller compartments, caused by some methyl groups protruding into the cavity. The occupied volume was calculated in single-probe mode with the following parameters:

Small probe radius: 0.65 Å for **1**, 2.0 Å for **3**

Grid resolution: 0.2 Å

Optimization depth: 4

Element radii: Mg: 2.510 Å; C: 1.770 Å; H: 1.200 Å; N: 1.660 Å; O: 1.500 Å.

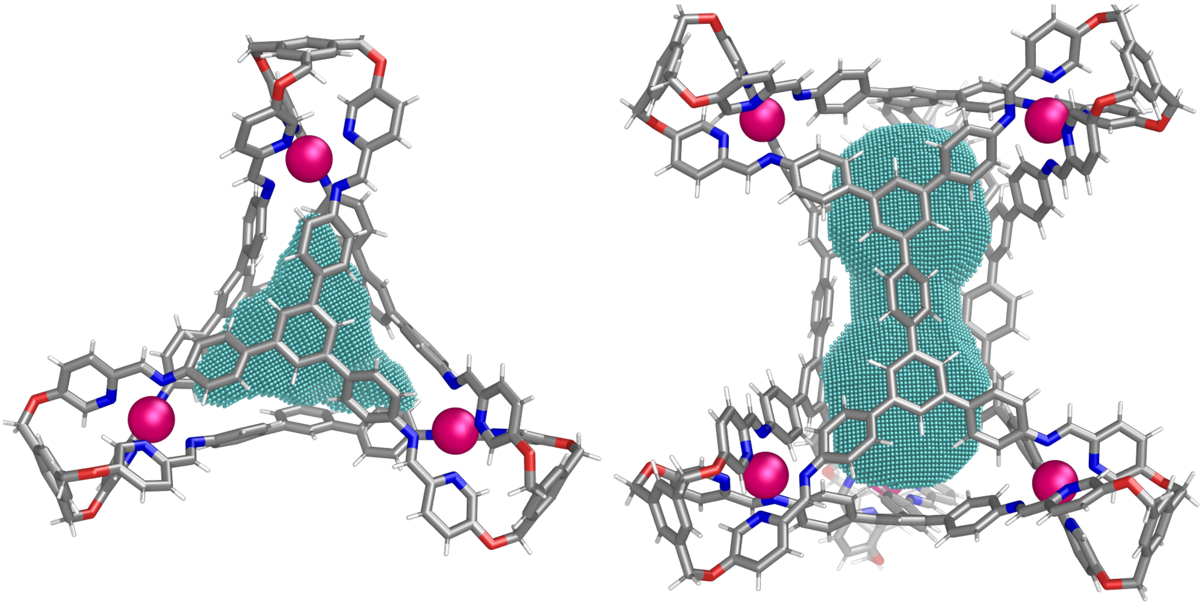

228 Å^3
2

683 Å^3
3

Figure S64. Cavity (shown in mesh) of cages **1** and **3**. Volume calculations were based on crystal structures.

S7 Study of photoluminescent properties

S7.1 General Procedure

All the photoluminescent (PL) spectra were recorded on an Edinburgh FLS1000 instrument with the excitation wavelength of 405 nm. The lifetimes of the cages were also recorded on this setup using a pulsed 405 nm laser.

Photoluminescent quantum yield (PLQY) was determined by the previously reported method[14]on a home-built linear PL setup equipped with a continuous-wave 375 nm laser diode, an Andor Kymera 328i spectrograph, an Andor iDus 420 CCD camera and necessary optical components. The sample in a 1 cm cuvette was excited with 375 nm at 5 mW measured by a power meter. An integral sphere and an optical fibre were also applied.

Emission decay profile of the cage **1** at 490 nm upon pulsed 405 nm excitation.

PL spectra, photoluminescence quantum yield (PLQY) measurements, and emission decay profiles were collected from as-prepared 1 mM cage solutions. UV–vis absorption spectra were recorded using 76.9 μM cage solutions.

S7.2 Other luminescent photos and spectra

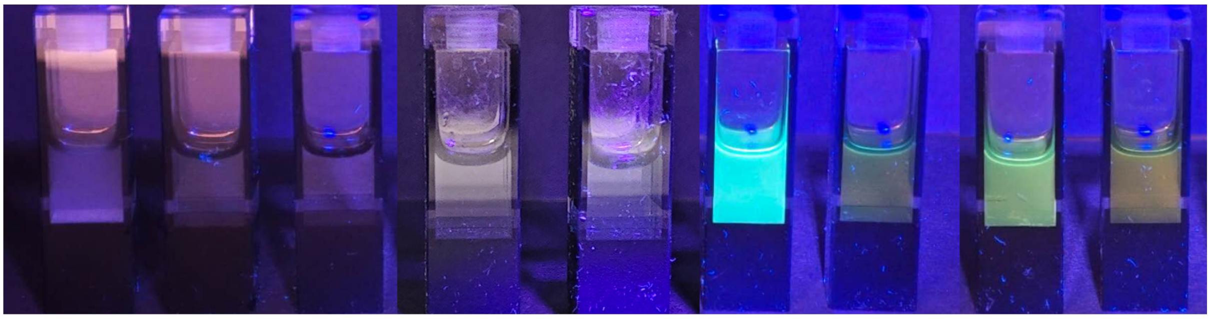

Figure S65. Photo taken under 390 nm UV light. Samples, from left to right: subcomponents **A**, **B**, and **C**; heated mixtures of **A**+**B** and **A**+**C**; and cages **1**, **1’**, **2**, and **2’**.

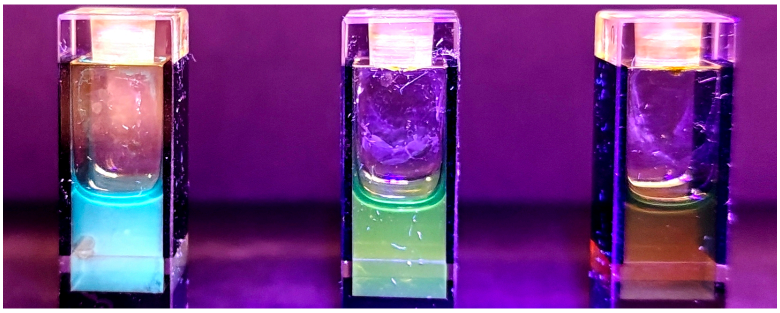

Figure S66. Photograph of the samples under 390 nm UV irradiation. From left to right: cages **1**, **2**, and **3**. Because cage **3** did not exhibit distinctive emission, its emission properties were not further investigated.

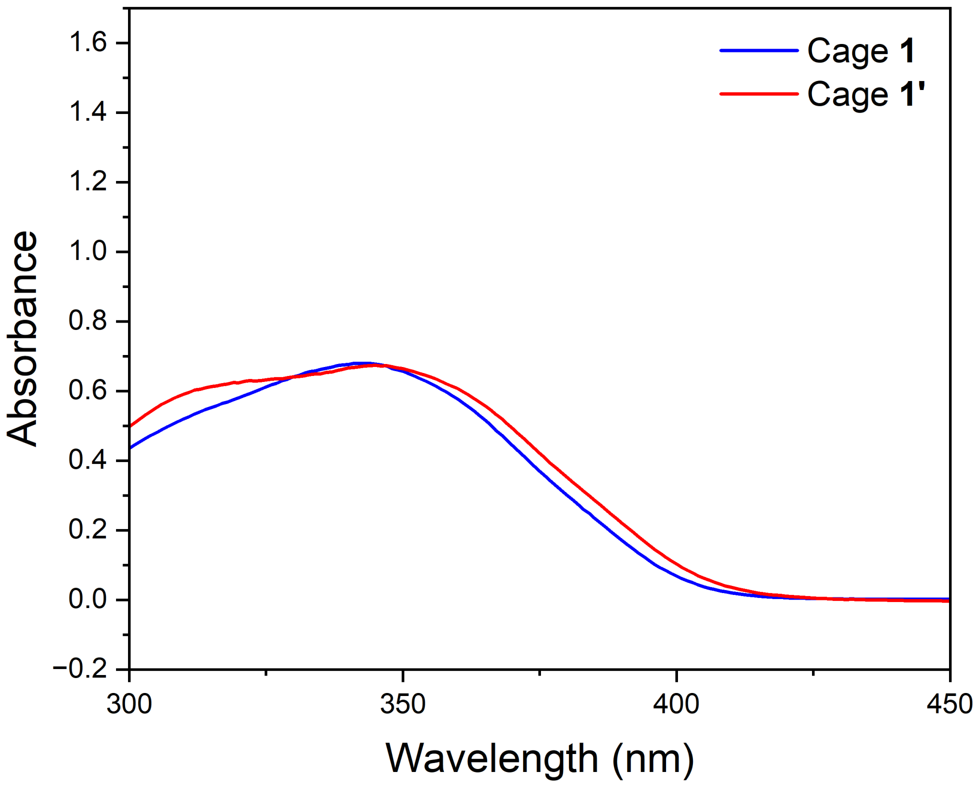

Figure S67. UV–vis spectra of cages **1** and **1'**, zoomed in to the 300–450 nm region. Both absorption maxima (**1**: 344 nm; **1'**: 350 nm) are attributed to imine n → π* transitions.[15]

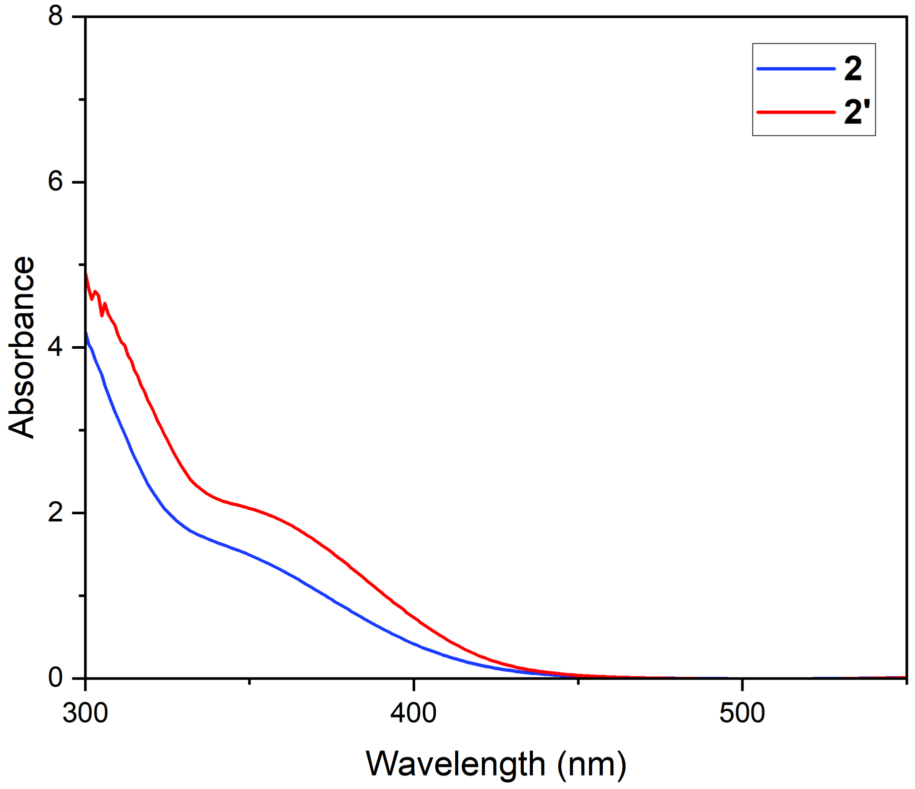

Figure S68. UV-vis spectra of cages **2** and **2’**. Both absorption maxima (**2**: 355 nm; **2’**: 365 nm) are attributed to imine n → π* transitions. [15]

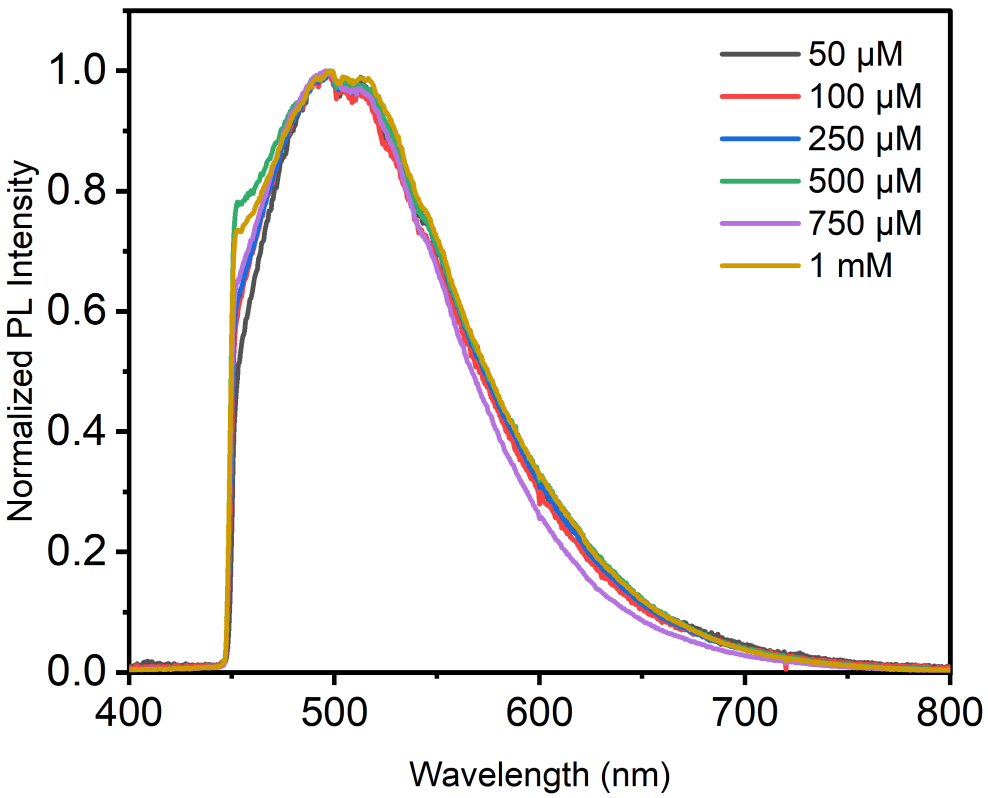

Figure S69. Normalized PL spectra of cage **1** at different concentrations under 405 nm excitation. No obvious shift in emission maximum or change in spectral profile is observed.

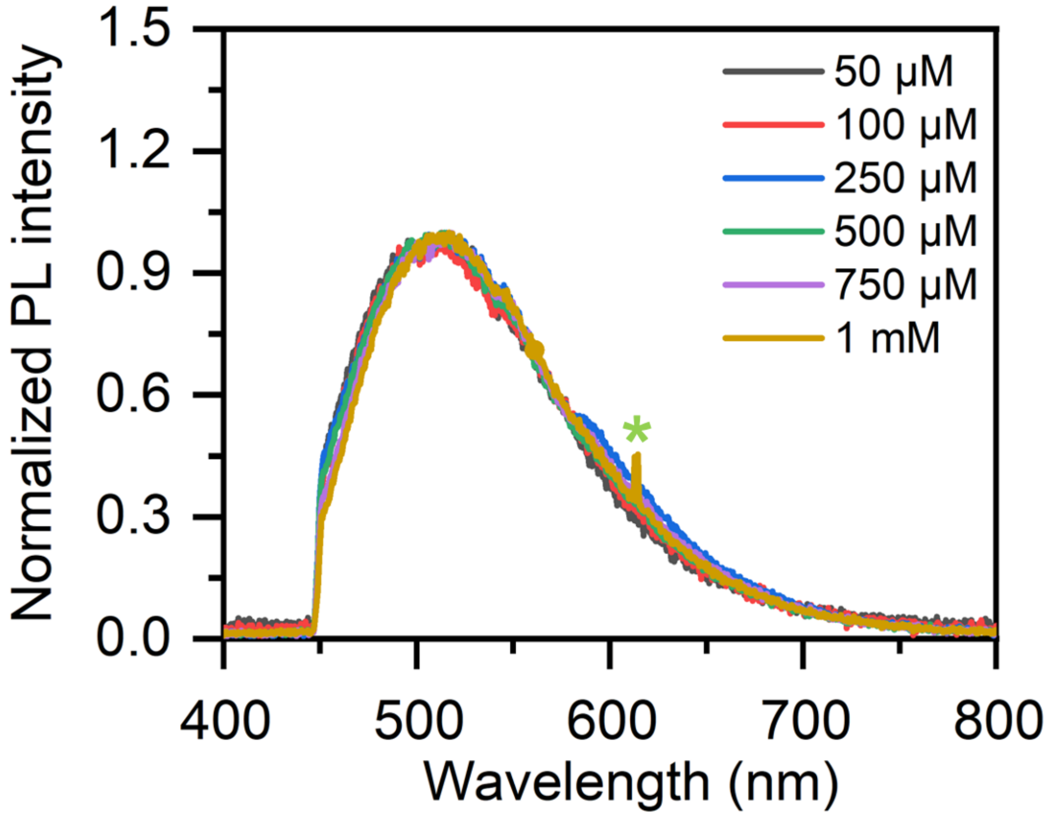

Figure S70. Normalized PL spectra of cage **1’** at different concentrations under 405 nm excitation. No obvious shift in emission maximum or change in spectral profile is observed. * indicates a stray instrumental spike observed in the 1 mM spectrum, unrelated to the intrinsic photoluminescence emission. The emissions are attributed to intraligand π–π* fluorescence emission.[16]

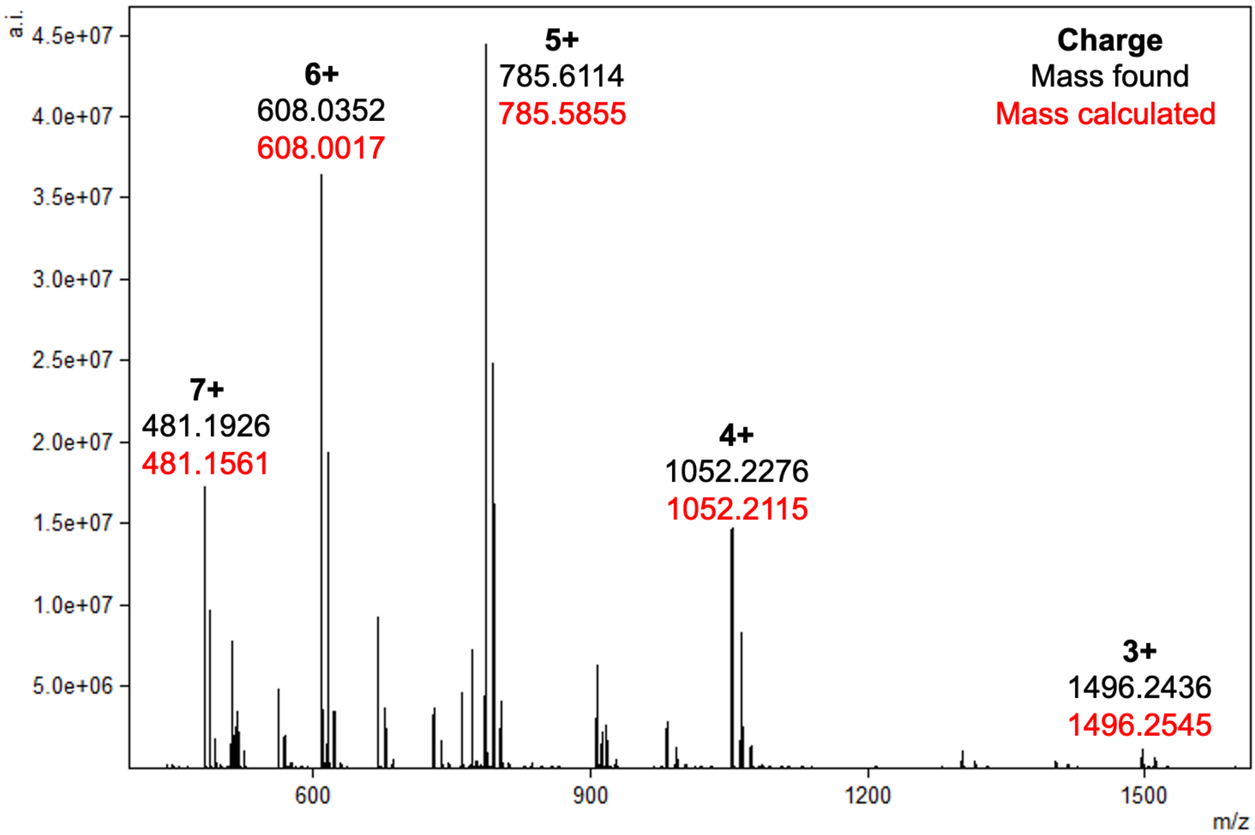

Figure S71. HR-ESI-MS spectrum of cage **1** taken at the 76.9 μM UV-vis measurement concentration.

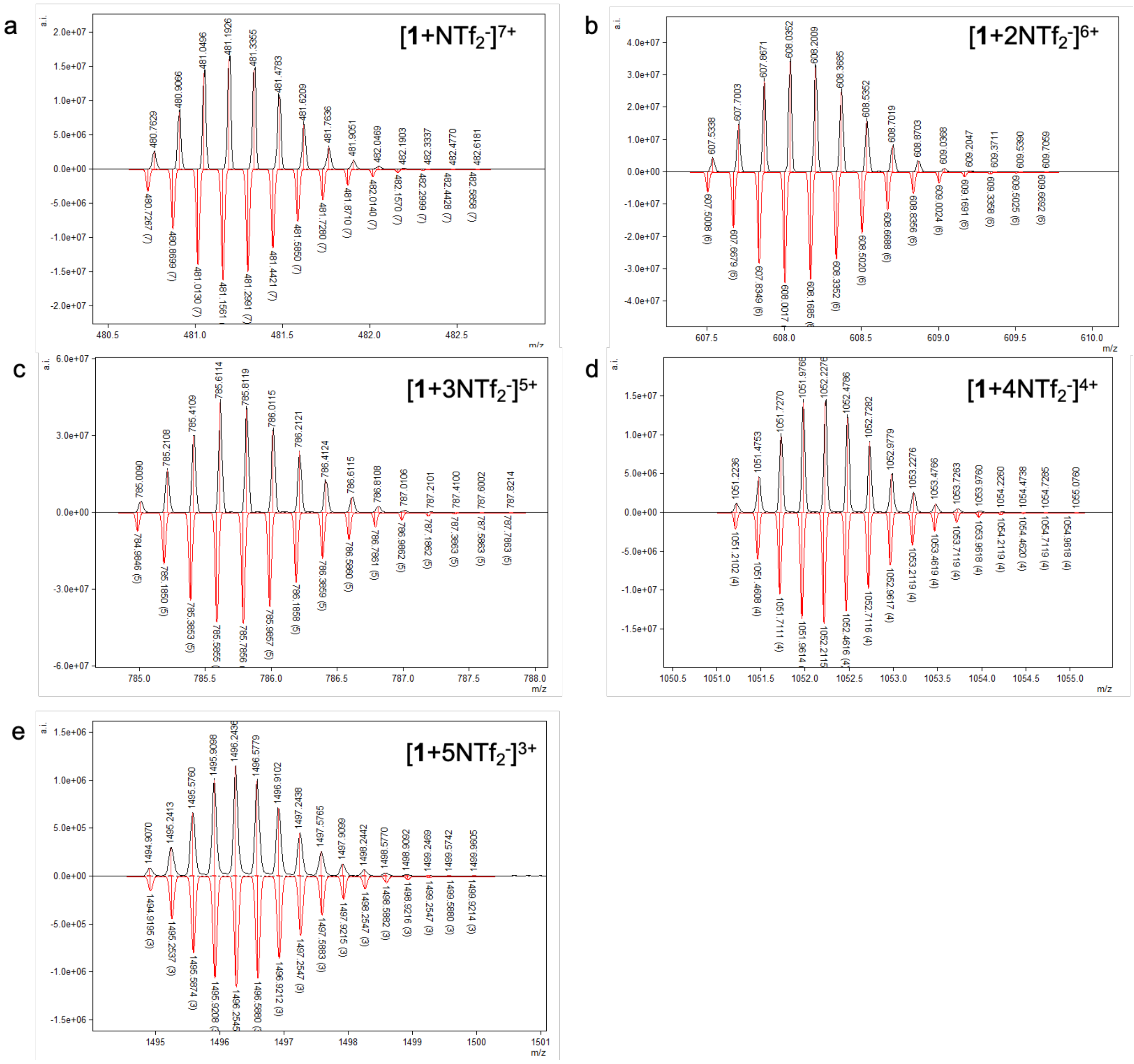

Figure S72. Signals from the HR-ESI-MS spectrum of **1** taken at the 76.9 μM UV-vis measurement concentration. Observed and calculated signals for (a) [**1**+NTf_2^-]$^{7+}$; (b) [**1**+2NTf_2^-]$^{6+}$; (c) [**1**+3NTf_2^-]$^{5+}$; (d) [**1**+4NTf_2^-]$^{4+}$; (e) [**1**+5NTf_2^-]$^{3+}$.

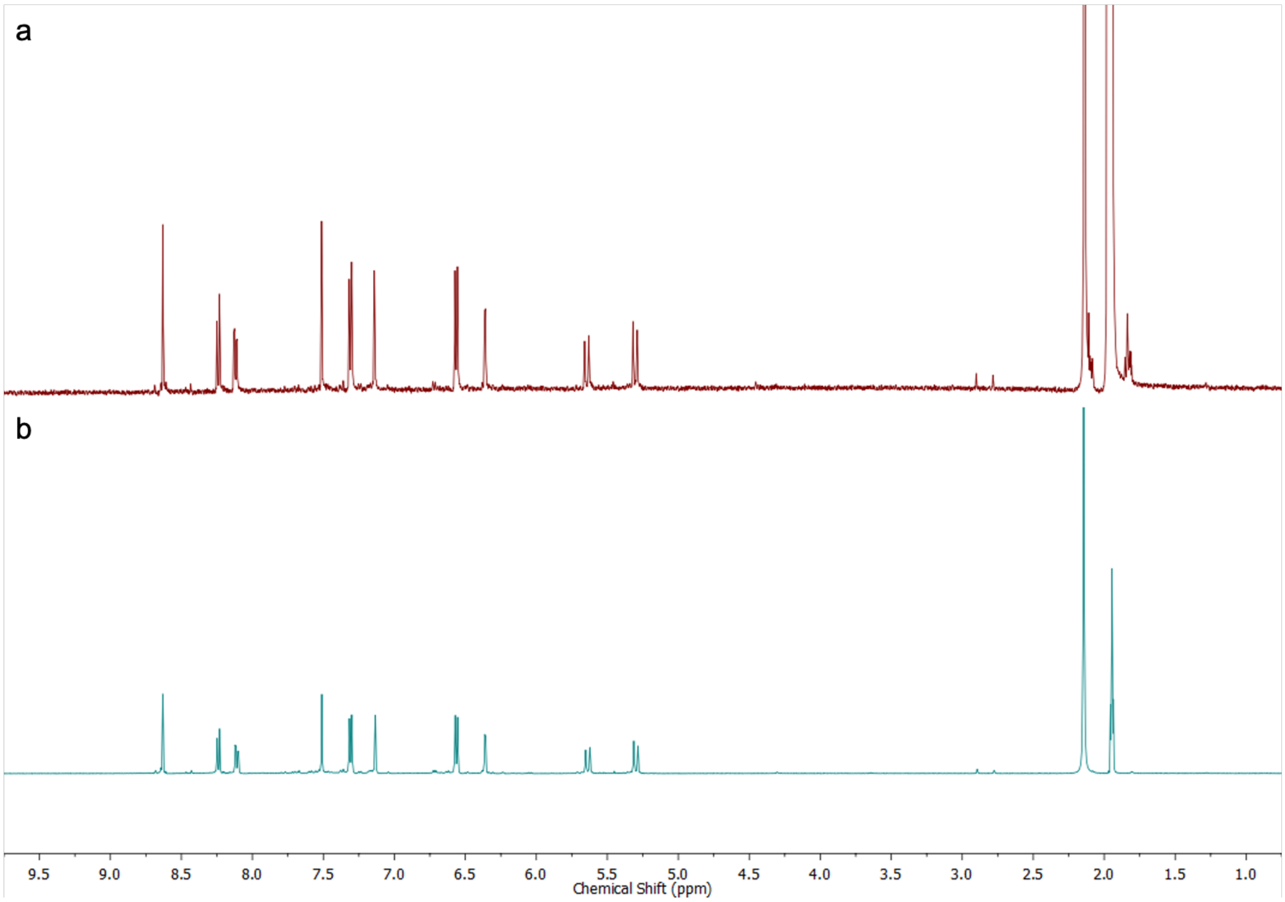

Figure S73. ^{1}H NMR spectra (500 MHz, 298 K, CD_3CN) of cage **2** recorded at (a) the 76.9 μM concentration used for UV–vis measurements and (b) 1 mM (as prepared).

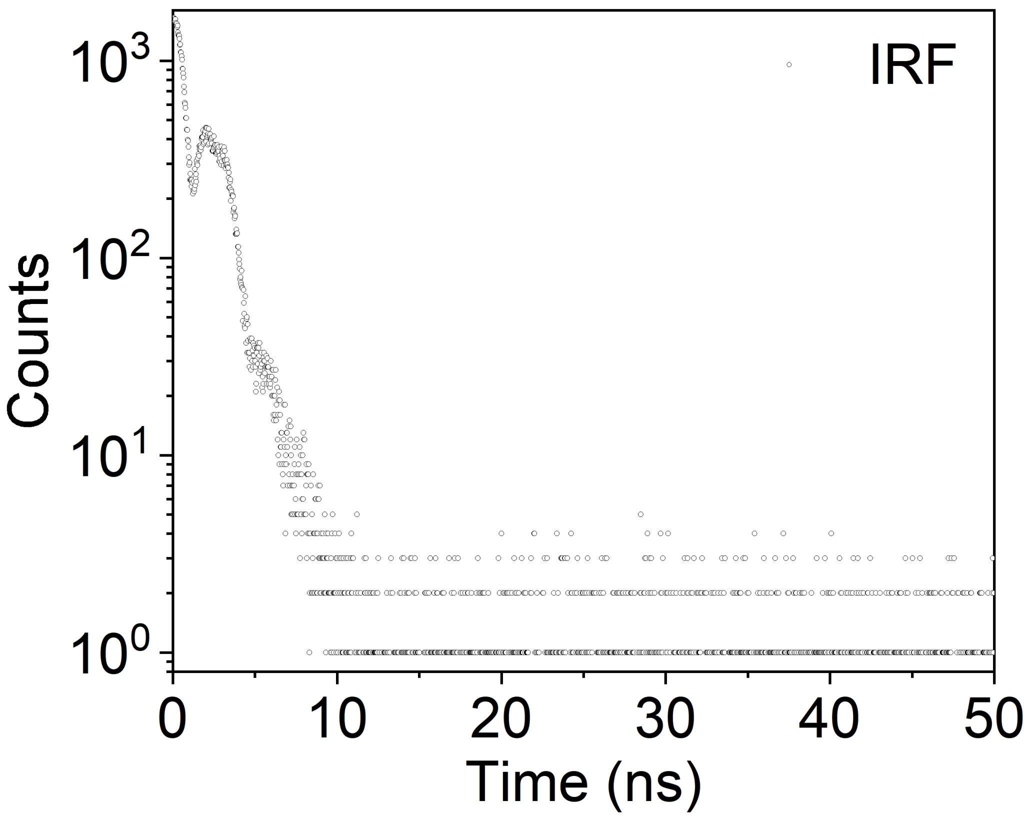

Figure S74. Instrument response function (IRF) recorded under 405 nm pulsed excitation for lifetime measurements.

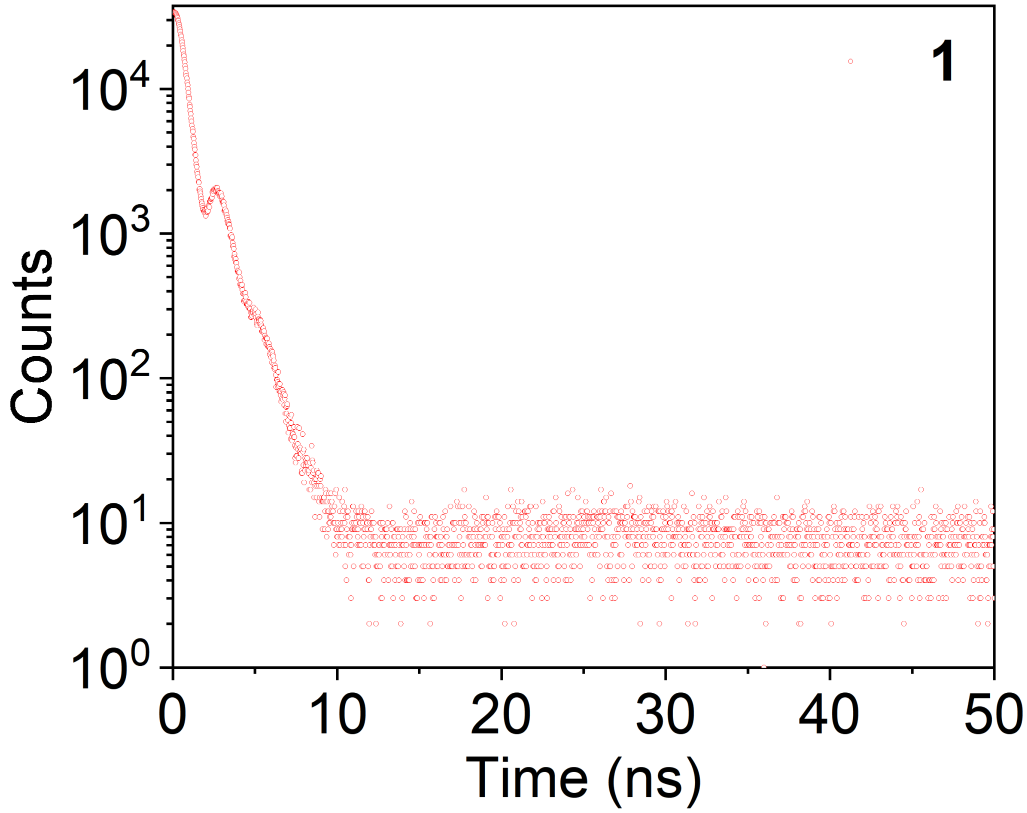

Figure S75. Emission decay profile of the cage **1** at 490 nm upon pulsed 405 nm excitation.

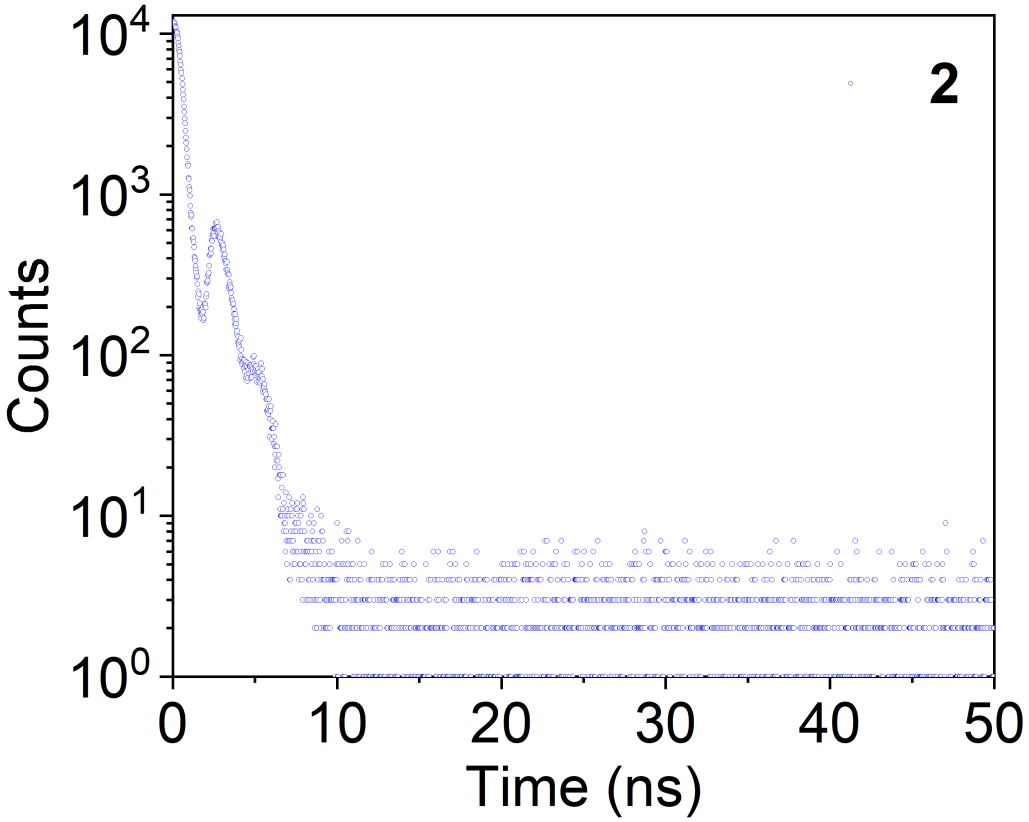

Figure S76. Emission decay profile of the cage **2** at 490 nm upon pulsed 405 nm excitation.

S8 Stability Tests in Various Solvents

S8.1 General Procedure

Solid powders of cages **1** and **2** were used in this study. Cage **1** (2.66 mg) or cage **2** (2.73 mg) was added to 0.5 mL of the corresponding solvent, and the resulting solution was sonicated for 5 min to aid dissolution. The resulting solution was then subjected to ^{1}H NMR measurement at room temperature. Subsequently, for samples in D_2O, CD_3OD, and $CDCl_3$, the solution was left at room temperature for 5 days and then centrifuged to recover the solid material. The recovered solid was re-dissolved in 0.5 mL of CD_3CN and subjected to ^{1}H NMR measurement. For samples in DMSO-d_6, since the ^{1}H NMR spectra showed cage signals with distinctly shifted peaks, a control experiment was performed: 10 drops of DMSO-d_6 were added to each cage solution in CD_3CN, and the resulting solutions were then subjected to ^{1}H NMR measurement.

S8.2 Stability in Water

Both cages appeared insoluble in D_2O, and no signals were observed in the ^{1}H NMR spectra shown below. The recovered solids also showed no cage-related signals after re-dissolution in CD_3CN, suggesting decomposition rather than simple insolubility. The cages are therefore considered unstable in water, presumably due to the high hydration energy of Mg^{2+} and the susceptibility of the imine bonds to hydrolysis.

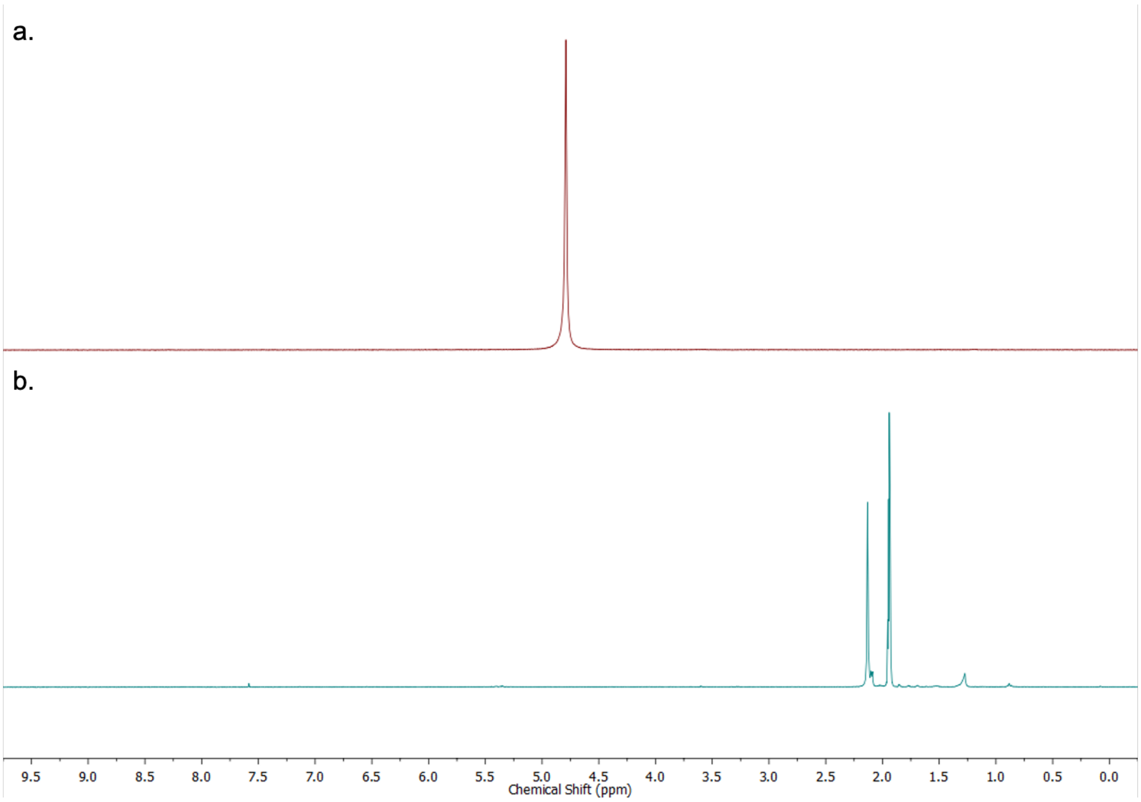

Figure S77. ^{1}H NMR spectra of (a) cage **1** in D_2O and (b) recovered cage **1** material re-dissolved in CD_3CN. No signals corresponding to cage species are observed in either spectrum.

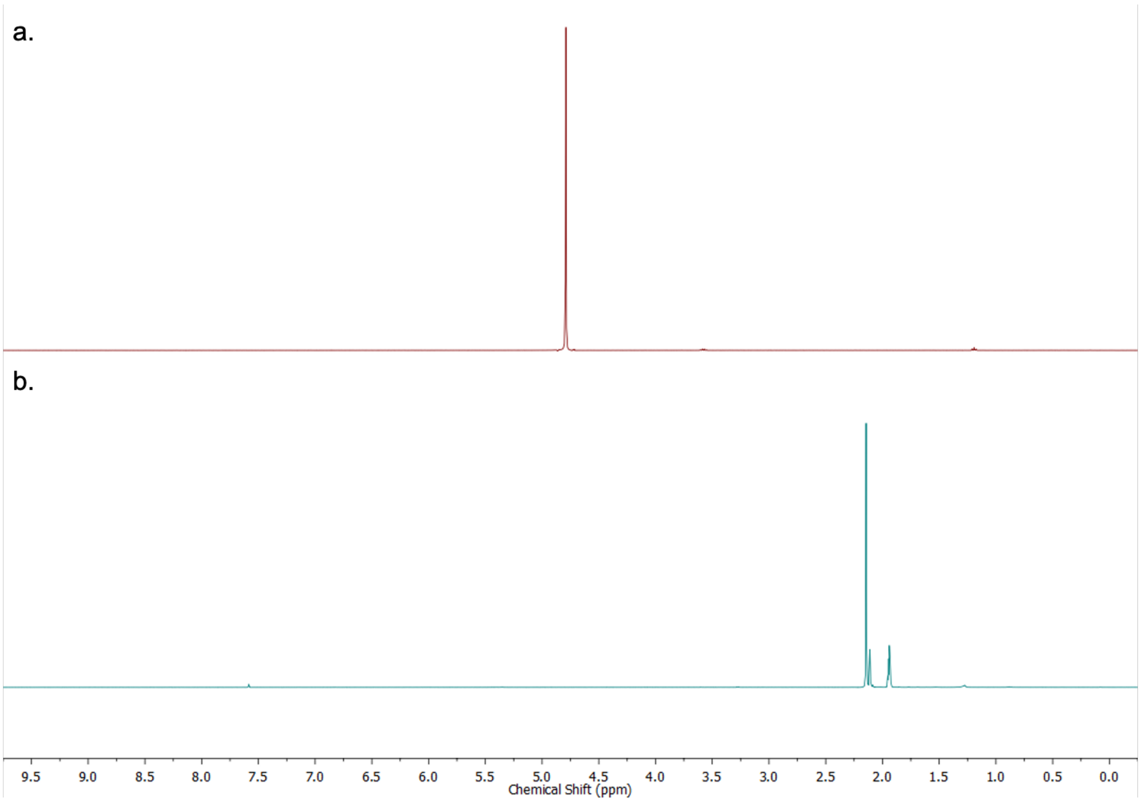

Figure S78. ^{1}H NMR spectra of (a) cage **2** in D_2O and (b) recovered cage **2** material re-dissolved in CD_3CN. No signals corresponding to cage species are observed in either spectrum.

S8.3 Stability in Methanol

Both cages appeared insoluble in CD_3OD, and no signals were observed in the ^{1}H NMR spectra shown below. The recovered solids also showed no cage-related signals after re-dissolution in CD_3CN, suggesting decomposition rather than simple insolubility. The cages are therefore considered unstable in methanol, presumably due to competitive coordination of Mg^{2+} by methanol oxygen donors.

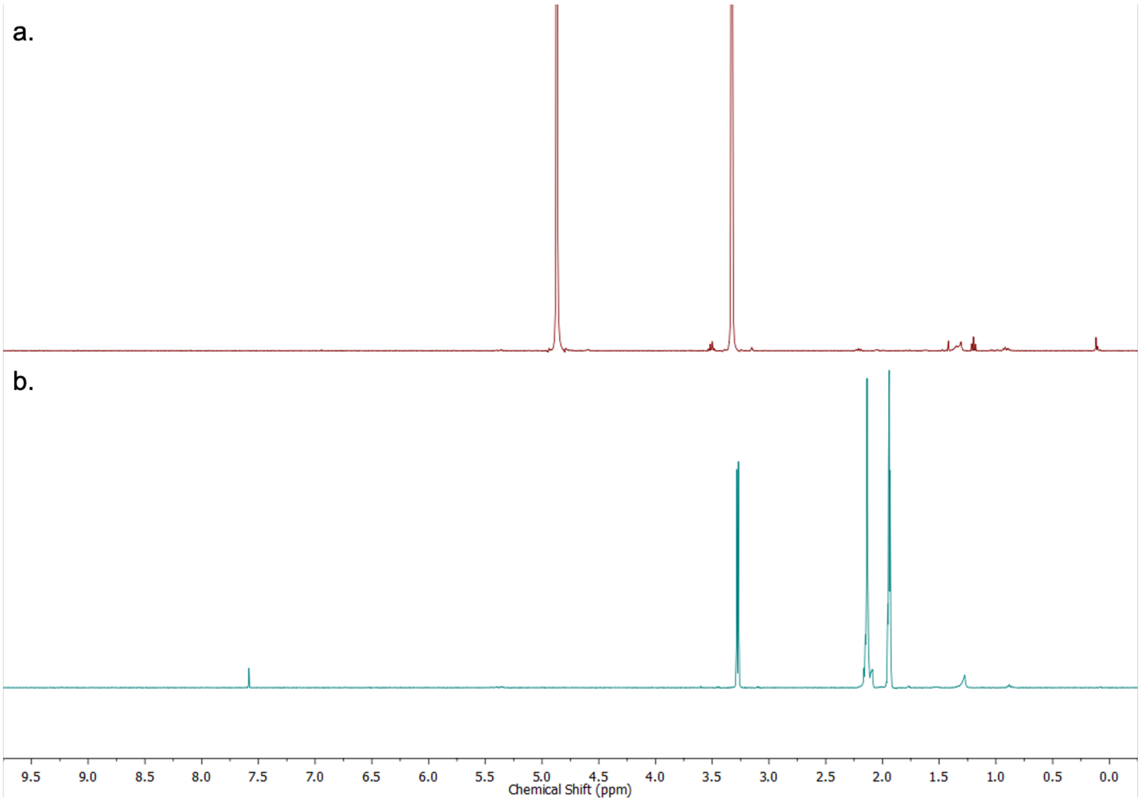

Figure S79. ^{1}H NMR spectra of (a) cage **1** in CD_3OD and (b) recovered cage **1** material re-dissolved in CD_3CN. No signals corresponding to cage species are observed in either spectrum.

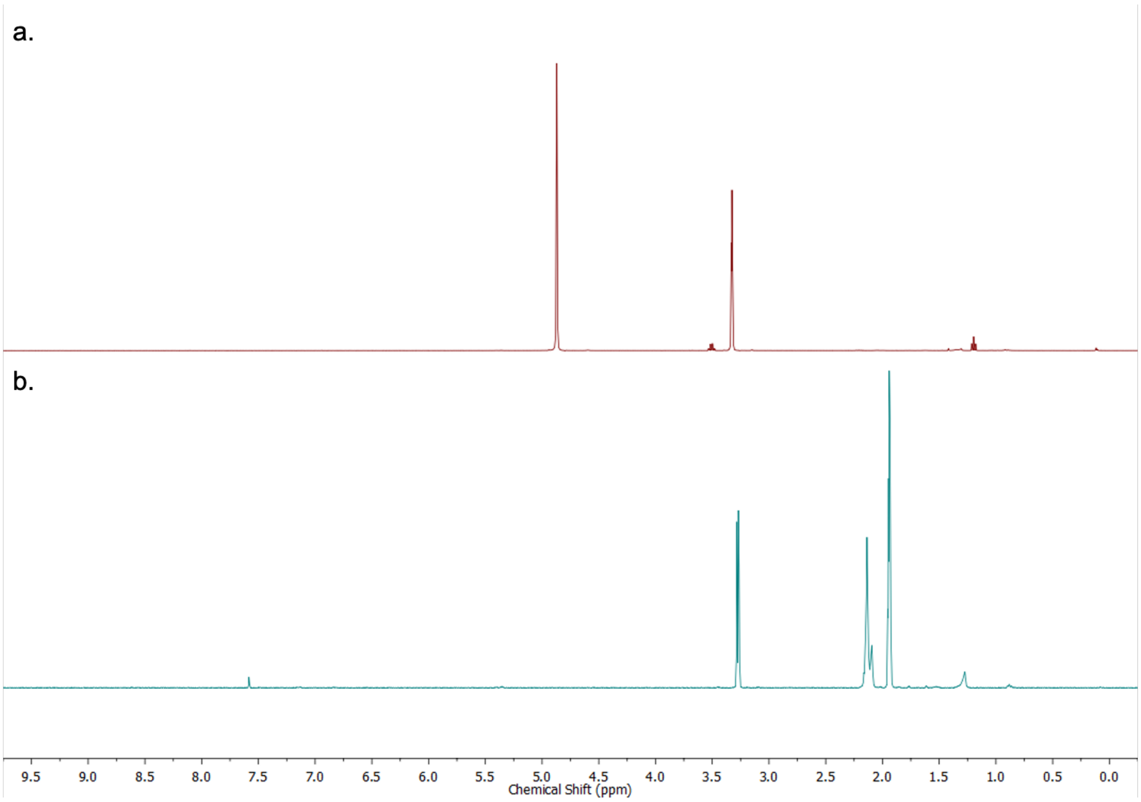

Figure S80. ^{1}H NMR spectra of (a) cage **2** in CD_3OD and (b) recovered cage **2** material re-dissolved in CD_3CN. No signals corresponding to cage species are observed in either spectrum.

S8.4 Stability in Dimethyl Sulfoxide

Both cages were partially soluble in DMSO-d_6, with white flocculent precipitates observed to form. T-symmetric cage-related signals were detected in the ^{1}H NMR spectra, suggesting that cage-like species remained present in solution. We speculated that these might correspond to demetallated organic assemblies. To test this assumption, a control experiment was conducted by adding 10 drops of DMSO-d_6 to each cage solution in CD_3CN. White precipitates formed immediately, and the resulting suspension showed no cage-related signals, consistent with decomposition or demetallation in the presence of DMSO-d_6.

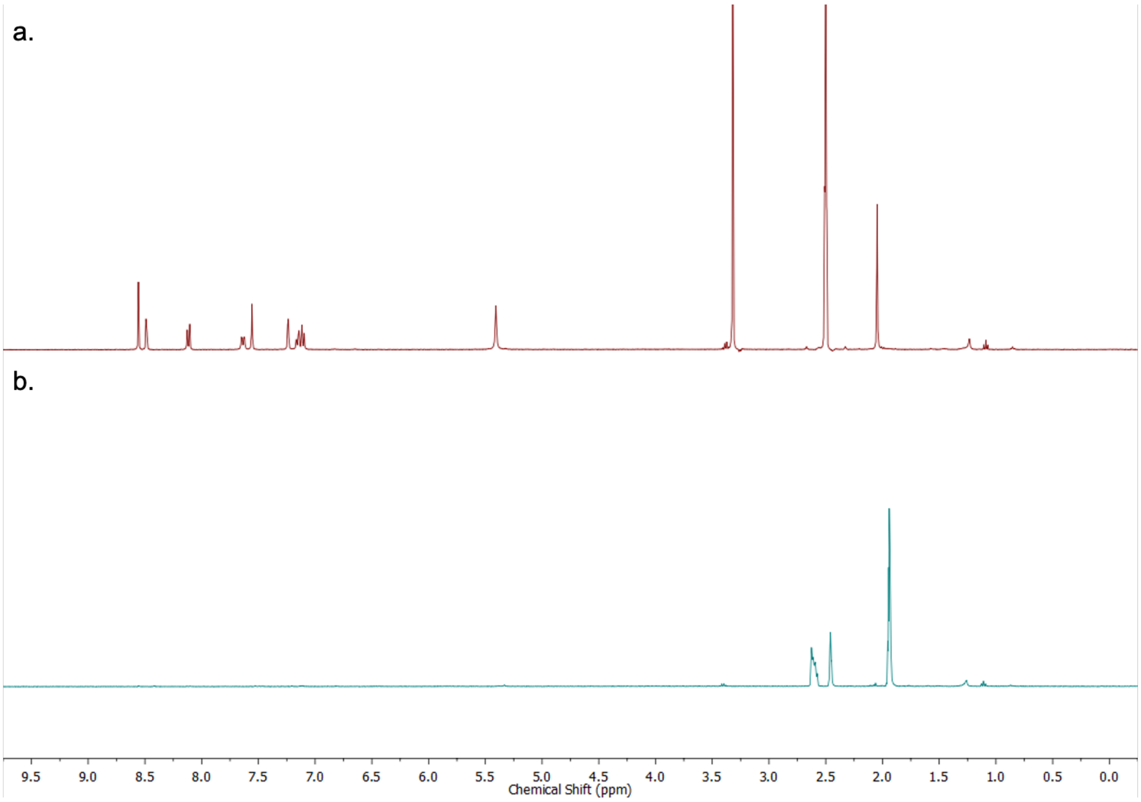

Figure S81. ^{1}H NMR spectra of (a) cage **1** in DMSO-d_6 and (b) cage **1** in CD_3CN with 10 drops of DMSO-d_6.

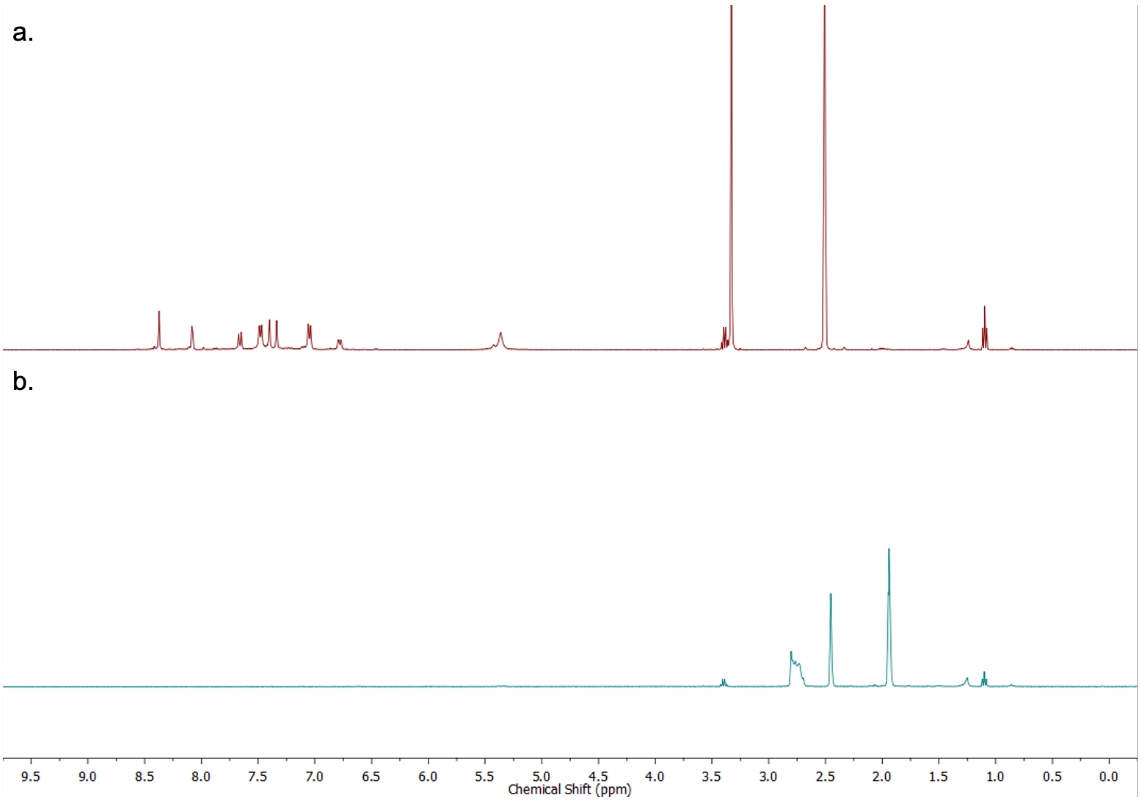

Figure S82. ^{1}H NMR spectra of (a) cage **2** in DMSO-d_6 and (b) cage **2** in CD_3CN with 10 drops of DMSO-d_6.

S8.5 Stability in Chloroform

Both cages were insoluble in $CDCl_3$, resulting in no observable signals in the ^1H NMR spectra shown below. After recovery, the solids exhibited the reappearance of the cage signals in CD_3CN, indicating that the cages remained stable but were simply insoluble in chloroform.

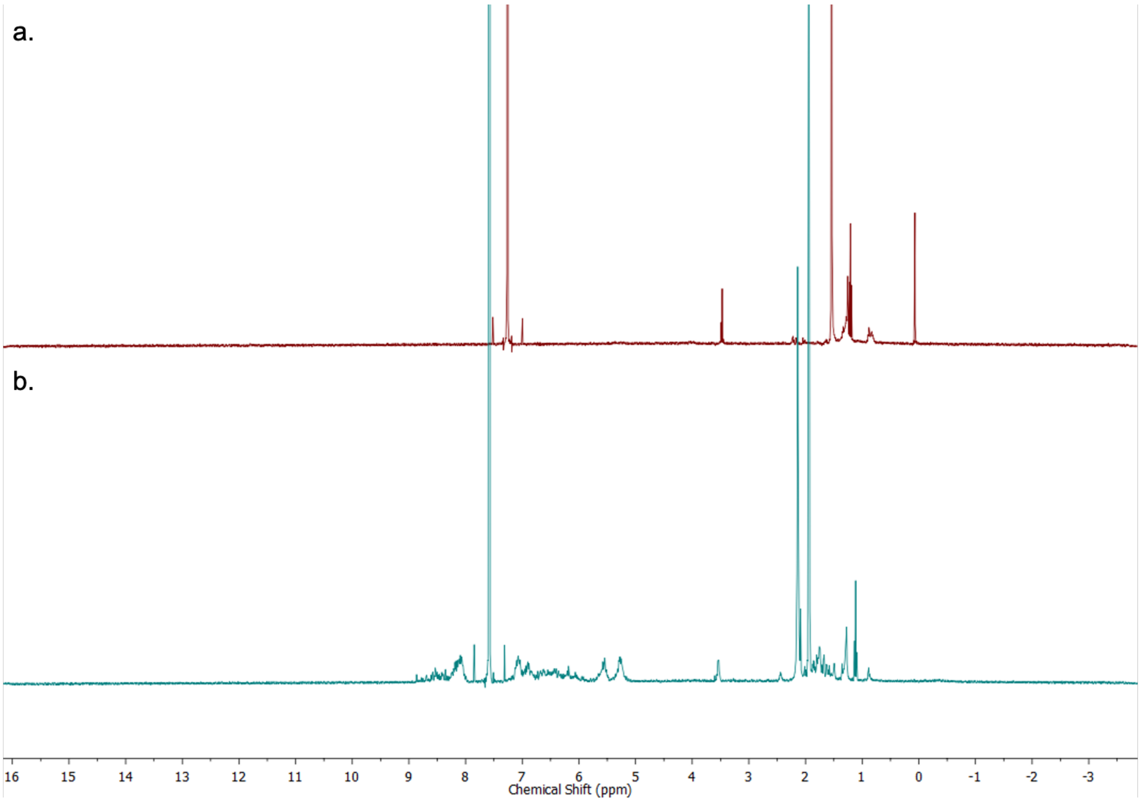

Figure S83. ^{1}H NMR spectra of (a) cage **1** in $CDCl_3$ and (b) recovered cage **1** material re-dissolved in CD_3CN.

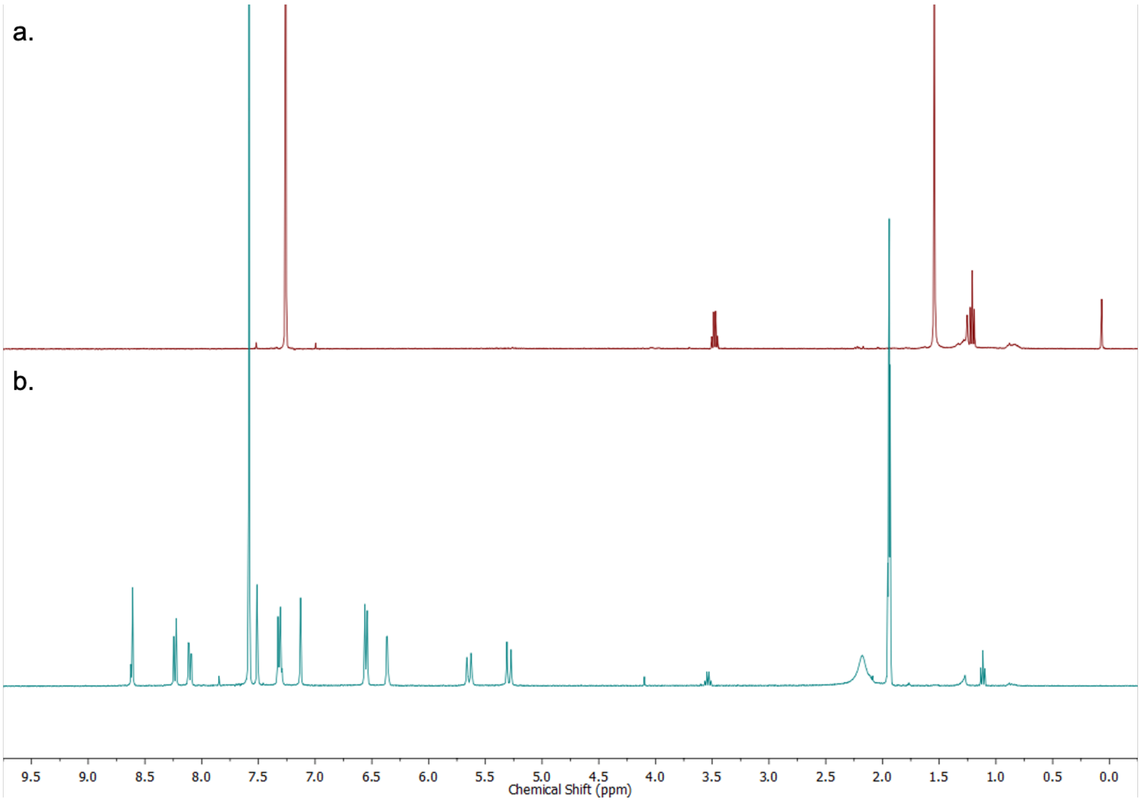

Figure S84. ^{1}H NMR spectra of (a) cage **2** in $CDCl_3$ and (b) recovered cage **2** material re-dissolved in CD_3CN.

S9 References

1. Davies, J. A.; Tarzia, A.; Ronson, T. K.; Auras, F.; Jelfs, K. E.; Nitschke, J. R. Tetramine Aspect Ratio and Flexibility Determine Framework Symmetry for Zn8L6 Self-Assembled Structures. *Angew. Chem. Int. Ed.,* **2023**, e202217987.

2. Lucotti, A.; Tommasini, M.; Fazzi D.; Zoppo, M. D.; Chalifoux, W. A.; Ferguson, M. J.; Zerbi, G.; Tykwinski, R. R. Evidence for Solution-State Nonlinearity of sp-Carbon Chains Based on IR and Raman Spectroscopy: Violation of Mutual Exclusion. *J. Am. Chem. Soc.* **2009**, *131*, 4239−4244.

3. Colomban, C.; Kudrik, E. V.; Afanasiev, P.; Sorokin, A. B. Catalytic Defluorination of Perfluorinated Aromatics under Oxidative Conditions Using N-Bridged Diiron Phthalocyanine. *J. Am. Chem. Soc.* **2014**, *136*, 11321−11330.

4. Bilbeisi, R. A.; Clegg, J. K.; Elgrishi, N.; de Hatten, X.; Devillard, M.; Breiner, B.; Mal, P.; Nitschke, J. R. Subcomponent Self-Assembly and Guest Binding Properties of Face-Capped $Fe_4L_4^{8+}$ Capsules. *J. Am. Chem. Soc*. **2012**, *134*, 5110–5119.

5. L.; Peach, A.; Hooper, M.; Zaja, L.; Patel, S.; Cahill, L.; Marshall, R.; Trimnell, S.; Foster, A.; Bates, T.; Lay, S.; Williams, M.; Hathaway, P.; Winter, G.; Gerstel, M.; Wooley, R., A Novel Dual Air-Bearing Fixed-χ Diffractometer for Small-Molecule Single-Crystal X-ray Diffraction on Beamline I19 at Diamond Light Source. *Crystals* **2017,** *7*, 336.

6. (a) Winter, G., xia2: an expert system for macromolecular crystallography data reduction. *J. Appl. Crystallogr.* **2010,** *43*, 186–190; (b) Winter, G.; Waterman, D. G.; Parkhurst, J. M.; Brewster, A. S.; Gildea, R. J.; Gerstel, M.; Fuentes-Montero, L.; Vollmar, M.; Michels-Clark, T.; Young, I. D.; Sauter, N. K.; Evans, G., DIALS: implementation and evaluation of a new integration package. *Acta Cryst.* **2018,** *D74*, 85–97.

7. Farrugia, L., WinGX and ORTEP for Windows: an update. *J. Appl. Crystallogr.* **2012,** *45*, 849–854.

8. Sheldrick, G., SHELXT - Integrated space-group and crystal-structure determination. *Acta. Cryst.* **2015,** *A71*, 3–8.

9. Sheldrick, G. M., Crystal structure refinement with SHELXL. *Acta. Cryst.* **2015,** *C71*, 3–8.

10. Smart, O. S.; Womack, T. O., *Grade Web Server*. Global Phasing Ltd.: 2014.

11. van der Sluis, P.; Spek, A. L., BYPASS: an effective method for the refinement of crystal structures containing disordered solvent regions. *Acta Cryst.* **1990,** *A46*, 194–201.

12. Spek, A. L., *PLATON: A Multipurpose Crystallographic Tool*. Utrecht University: Utrecht, The Netherlands, 2008.

13. Maglic, J. B.; Lavendomme, R. *MoloVol*: an easy-to-use program for analyzing cavities, volumes and surface areas of chemical structures. *J. Appl. Crystallogr.* **2022**, *55*, 1033–1044.
14. de Mello, J. C.; Wittmann, H. F.; Friend, R. H. An improved experimental determination of external photoluminescence quantum efficiency. *Adv. Mater*. **1997**, *9*, 230–232.
15. Cao, J.; Jia, Z.; Chen, W.; Song, Y.; Yu, Z.; Dong, Y.; Ren, Y. Synthesis, crystal structures, fluorescence properties, theoretical calculations and substitution effect of mono zinc(II) Schiff base complexes. *Inorg. Chim. Acta.* **2025**, *577*, 122494.
16. Basu Baul, T. S.; Kundu, S.; Linden, A.; Raviprakash, N.; Manna, S. K.; Guedes da Silva, M. F. C. Synthesis and characterization of some water soluble Zn(ii) complexes with (E)-N-(pyridin-2-ylmethylene)arylamines that regulate tumour cell death by interacting with DNA. *Dalton Trans.* **2014**, *43*, 1191–1202.
